# Supplementary material for: Cross-sectional online survey of the impact of new tobacco health warnings in Colombia
Source: BMJ Open. 2022 Jun 27;12(6):e056754. doi: 10.1136/bmjopen-2021-056754 (PMC9237901; doi:10.1136/bmjopen-2021-056754)

Supplementary Materials Table 1. Survey Questions

Variable	Items	Response
Smoking behaviour - eligibility	Have you smoked at least 100 cigarettes in your lifetime?	1 = Yes<br>2 = No
	How often do you smoke cigarettes?	1 = Every day<br>2 = Every week<br>3 = Less than every week<br>4 = Not al all
Smoking heaviness – daily smokers	Approximately how many cigarettes do you smoke per day?	Provide a single number
Smoking heaviness -non-daily smokers	Approximately how many days a week do you smoke	
	On the days that you do smoke, approximately how many cigarettes do you smoke	
	Approximately how many cigarettes do you smoke per week?	
Fagerström Test for Nicotine Dependence (FTND)	How soon after you wake do you smoke your first cigarette?	1 = Within 5 minutes<br>2 = 6 to 30 minutes<br>3 = 31 to 60 minutes<br>4 = After 60 minutes
Awareness of new warnings	Have you seen this health warning on a cigarette pack?	1= Yes<br>2 = No<br>3 = Unsure
Responses to warnings seen on tobacco packs		
Negative affect	In the last week, the warning on my cigarette pack made me feel...	
	…scared	0 = Strongly disagree<br>25 = Somewhat disagree<br>50 = Neither agree nor disagree<br>75 = Somewhat agree<br>100 = Strongly agree
	…regretful	
	…disgusted	

Thinking about the warning message	When I notice my cigarette pack, I think about the message that the warning conveys	0 = Strongly disagree<br>25 = Somewhat disagree<br>50 = Neither agree nor disagree<br>75 = Somewhat agree<br>100 = Strongly agree
	When my cigarette pack is not in sight, I think about the message that the warning conveys	
Attitudes towards smoking		
Perceived likelihood of harm	What is the chance that you will one day get the following diseases if you continue to smoke cigarettes?	
	…heart disease?	0 = No chance<br>25 = Low chance<br>50 = Moderate chance<br>75 = High chance<br>100 = Certain
	…cancer?	
	…a permanent breathing problem?	
Perceived severity of harm from smoking	How much would getting the following diseases because of smoking affect your life?	
	…heart disease?	0 = No chance<br>25 = Low chance<br>50 = Moderate chance<br>75 = High chance<br>100 = Certain
	…cancer?	
	…a permanent breathing problem?	
Self-efficacy	I see myself as being capable of quitting smoking in the next 2 months if I wanted to	0 = Strongly disagree<br>25 = Somewhat disagree<br>50 = Neither agree nor disagree<br>75 = Somewhat agree<br>100 = Strongly agree
Response-efficacy	Quitting smoking would lower my chances of getting a disease related to smoking	

Quit intentions	Are you planning to quit smoking within the next 6 months?	0 = Very unlikely<br>25 = Unlikely<br>50 = Maybe, maybe not<br>75 = Likely<br>100 = Very likely
Responses to new warnings		
Negative affect	This warning makes me feel…	
	…scared	0 = Strongly disagree<br>25 = Somewhat disagree<br>50 = Neither agree nor disagree<br>75 = Somewhat agree<br>100 = Strongly agree
	…regretful	
	…disgusted	
Believability	This warning is believable	0 = Strongly disagree<br>25 = Somewhat disagree<br>50 = Neither agree nor disagree<br>75 = Somewhat agree<br>100 = Strongly agree
Thinking about harms	This warning makes me think about the health problems caused by smoking	0 = Strongly disagree<br>25 = Somewhat disagree<br>50 = Neither agree nor disagree<br>75 = Somewhat agree<br>100 = Strongly agree
Reactance	This warning is trying to manipulate me	0 = Strongly disagree<br>25 = Somewhat disagree<br>50 = Neither agree nor disagree<br>75 = Somewhat agree<br>100 = Strongly agree
	The health effect of this warning is overblown	
	This warning annoys me	
Perceived message effectiveness	This warning discourages me from wanting to smoke	0 = Strongly disagree<br>25 = Somewhat disagree<br>50 = Neither agree nor disagree<br>75 = Somewhat agree<br>100 = Strongly agree
	This warning makes smoking seem unpleasant to me	
	This warning makes me concerned about the health effects of smoking	
	If I saw this warning on my	

	cigarette pack, I would...	
	...try to avoid thinking about it	
	…try to avoid looking at it	
	…keep the pack out of sight to avoid looking at the warning	
Knowledge of health risks	Say how much you agree or disagree with each statement below:	0 = Strongly disagree<br>25 = Somewhat disagree<br>50 = Neither agree nor disagree<br>75 = Somewhat agree<br>100 = Strongly agree
	Smoking while pregnant could cause you to lose your baby	
	Smoking causes pancreatic cancer	
	Secondhand smoke is deadly	
	Smoking damages your heart	
	Smoking causes anxiety	
	Smoking causes a slow and painful death	

Supplementary Material – Table 2. Internal consistency of latent constructs

**Latent construct**	**Number of items**	**α**	***r***
Responses to warnings on packs			
Negative affect	3 items	0.79	0.49 – 0.69
Thinking about warning message	2 items	0.66	0.5
Cognitive elaboration	2 items	0.8	0.66
Attitudes to smoking			
Perceived likelihood of harm	3 items	0.84	0.61-0.65
Perceived severity	3 items	0.87	0.66-0.71
Responses to new warnings			
**Warning 1 "Pancreatic cancer"**			
Negative affect	3 items	0.83	0.54 – 0.77
Reactance	3 items	0.68	0.39 – 0.45
Perceived effectiveness	6 items	0.87	0.34 – 0.82
**Warning 2 "Miscarriage"**			
Negative affect	3 items	0.77	0.38 – 0.79
Reactance	3 items	0.69	0.41 – 0.44
Perceived effectiveness	6 items	0.86	0.28 – 0.75
**Warning 3 "Death"**			
Negative affect	3 items	0.8	0.46 – 0.77
Reactance	3 items	0.69	0.43 – 0.44
Perceived effectiveness	6 items	0.86	0.33 – 0.82
**Warning 4 "Secondhand Smoke"**			
Negative affect	3 items	0.81	0.49 – 0.67
Reactance	3 items	0.67	0.37 – 0.44
Perceived effectiveness	6 items	0.86	0.31 – 0.80
**Warning 5 "Heart disease"**			
Negative affect	3 items	0.83	0.53 – 0.80
Reactance	3 items	0.68	0.40 – 0.44
Perceived effectiveness	6 items	0.86	0.33 – 0.80
**Warning 6 "Anxiety"**			
Negative affect	3 items	0.87	0.63 – 0.76
Reactance	3 items	0.64	0.33 – 0.43
Perceived effectiveness	6 items	0.87	0.34 – 0.85

Note. The internal consistency of latent constructs assessed using Cronbach's alpha and associations between the constructs using bivariate correlations.

Supplementary Materials Table 3. – Presence of warning theme between 2010 and 2018.

**Warning theme**	**2010**	**2011**	**2012**	**2013**	**2014**	**2015**	**2016**	**2017**	**2018**
Death							X		X
Secondhand smoke		X			X			X	X
Anxiety									X
Miscarriage		X		X			X	X	X
Heart attack / heart disease					X		X	X	X
Pancreatic cancer									X
Mouth cancer and bad breath	X								
Kidney cancer				X				X	
Breast cancer				X					
Leukemia					X				
Bladder cancer					X				
Laryngeal cancer							X		
Cancer							X		
Damaged arteries					X			X	
Thrombosis					X				
Vascular problems / disease				X					
Cerebral stroke		X							
Lung disease		X					X		
Eye problems / disease		X							
Bone damage				X					
Sexual impotence	X	X		X					
Aging	X								

Data are taken from https://www.tobaccocontrollaws.org/legislation/country/colombia/laws.
Data not available for 2012 or 2015.

All warnings are available from the Colombian Ministry of Health and Social Protection.

Colombia. Ministerio de Salud y Protección Social. (2020). Advertencias sanitarias productos de tabaco y derivados 2010 - 2021. [Piezas publicitarias;] Retrieved From: https://www.minsalud.gov.co/sites/rid/Lists/BibliotecaDigital/RIDE/VS/PP/ENT/advertencias-sanitarias-todas-vigencias.zip

Supplementary Material - Figure 1. Six new tobacco health warnings introduced in Colombia July 2018

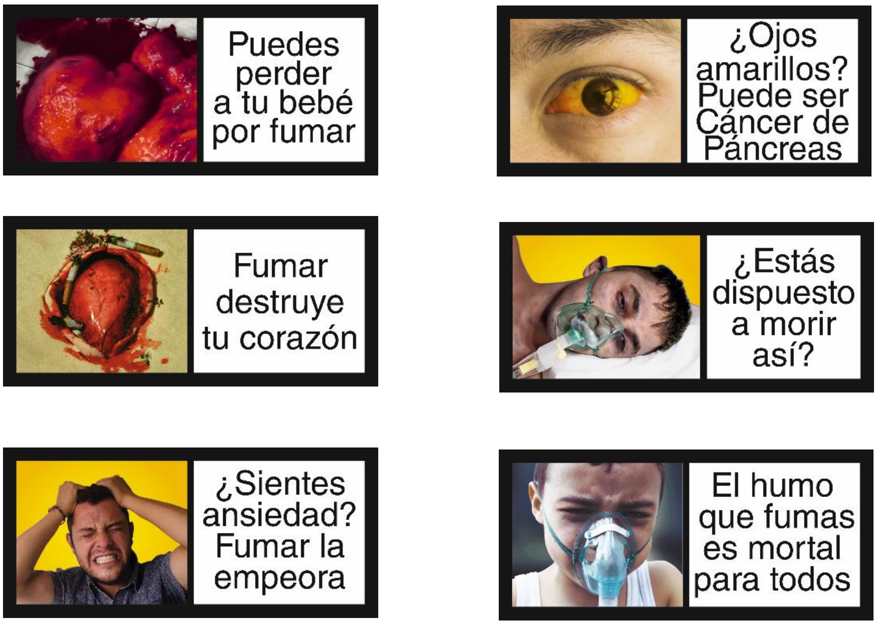

Health warning topics – clockwise *"You can lose your baby by smoking", "Yellow eyes? It could be pancreatic cancer", "Smoking destroys your heart", "Are you willing to die like this?", "Do you feel anxiety? Smoking makes it worse"* and *"The smoke you smoke is deadly for everyone".*

Supplementary Material – Figure 2. Percentage of participants who report having seen the new tobacco health warnings by data collection wave and smoker type (i.e., buys cigarette packs, buys packs and singles, buys singles).

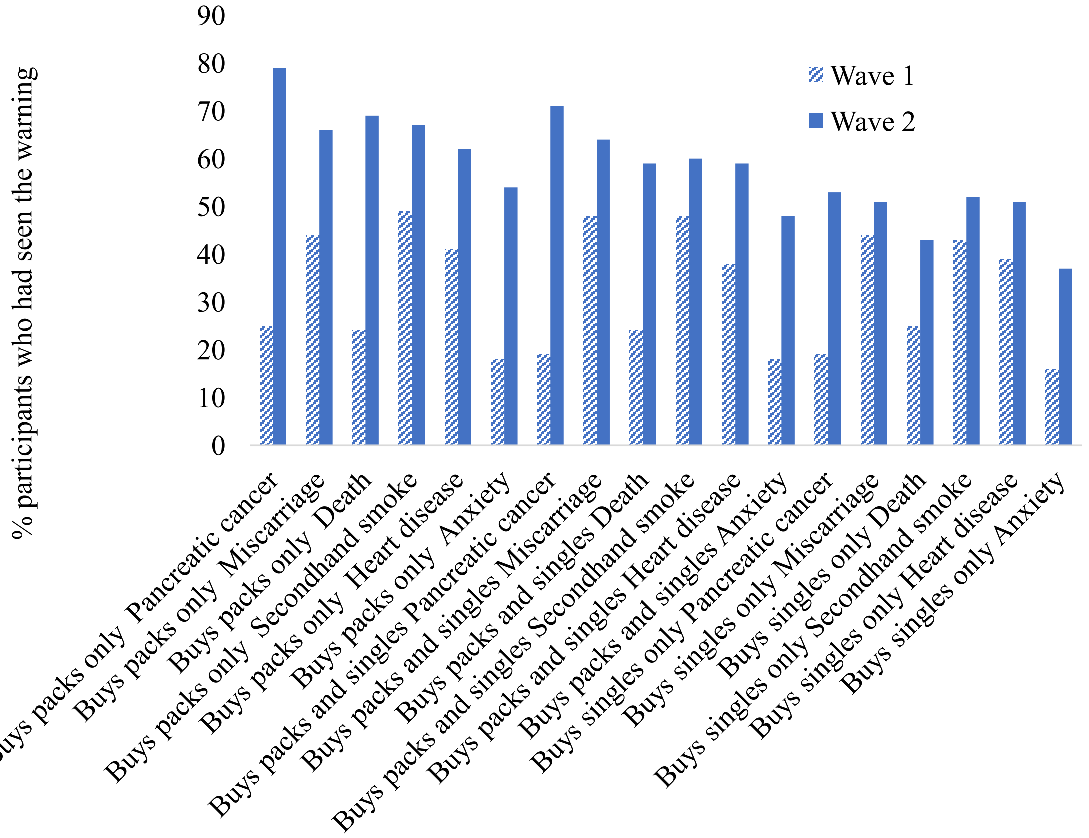

Supplementary Material – Figure 3. Percentage of participants who report having seen the new tobacco health warnings by data collection wave and smoking status (i.e., daily smoker, weekly smoker).

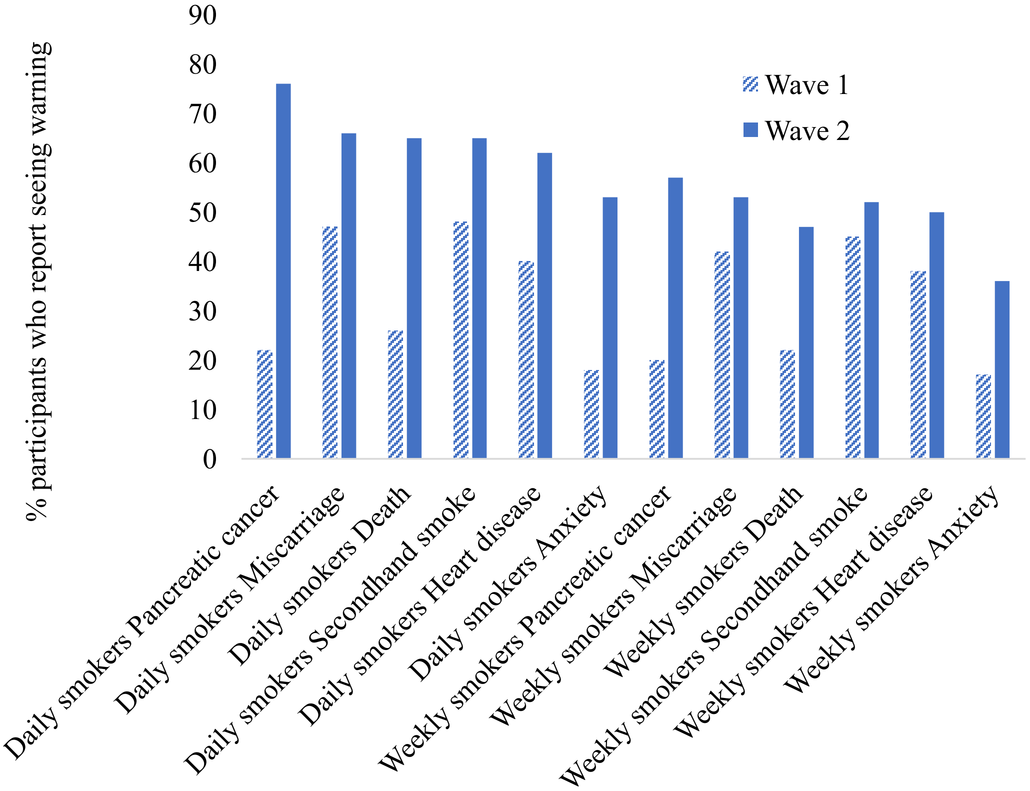

Supplement: Supplementary data [file bmjopen-2021-056754supp001.pdf]
