# Supplementary material for: Cross-sectional online survey of the impact of new tobacco health warnings in Colombia
Source: BMJ Open. 2022 Jun 27;12(6):e056754. doi: 10.1136/bmjopen-2021-056754 (PMC9237901; doi:10.1136/bmjopen-2021-056754)
Supplement: Author's manuscript [file bmjopen-2021-056754.draft_revisions.pdf]

BMJ Open

BMJ Open is committed to open peer review. As part of this commitment we make the peer review history of every article we publish publicly available.

When an article is published we post the peer reviewers' comments and the authors' responses online. We also post the versions of the paper that were used during peer review. These are the versions that the peer review comments apply to.

The versions of the paper that follow are the versions that were submitted during the peer review process. They are not the versions of record or the final published versions. They should not be cited or distributed as the published version of this manuscript.

BMJ Open is an open access journal and the full, final, typeset and author-corrected version of record of the manuscript is available on our site with no access controls, subscription charges or pay-per-view fees (http://bmjopen.bmj.com).

If you have any questions on BMJ Open's open peer review process please email info.bmjopen@bmj.com

BMJ Open

The impact of new tobacco health warnings in Colombia

Journal:	*BMJ Open*
Manuscript ID	bmjopen-2021-056754
Article Type:	Original research
Date Submitted by the Author:	25-Aug-2021
Complete List of Authors:	Adams, Sally; University of Bath, Psychology<br>Clavijo, Arturo; Universidad Nacional de Colombia, Psychology<br>Tamayo, Ricardo ; Universidad Nacional de Colombia, Psychology<br>Maynard, Olivia ; Univ Bristol, School of Experimental Psychology
Keywords:	Health policy < HEALTH SERVICES ADMINISTRATION & MANAGEMENT, Substance misuse < PSYCHIATRY, PUBLIC HEALTH

SCHOLARONE™
Manuscripts

I, the Submitting Author has the right to grant and does grant on behalf of all authors of the Work (as defined in the below author licence), an exclusive licence and/or a non-exclusive licence for contributions from authors who are: i) UK Crown employees; ii) where BMJ has agreed a CC-BY licence shall apply, and/or iii) in accordance with the terms applicable for US Federal Government officers or employees acting as part of their official duties; on a worldwide, perpetual, irrevocable, royalty-free basis to BMJ Publishing Group Ltd ("BMJ") its licensees and where the relevant Journal is co-owned by BMJ to the co-owners of the Journal, to publish the Work in this journal and any other BMJ products and to exploit all rights, as set out in our licence.

The Submitting Author accepts and understands that any supply made under these terms is made by BMJ to the Submitting Author unless you are acting as an employee on behalf of your employer or a postgraduate student of an affiliated institution which is paying any applicable article publishing charge ("APC") for Open Access articles. Where the Submitting Author wishes to make the Work available on an Open Access basis (and intends to pay the relevant APC), the terms of reuse of such Open Access shall be governed by a Creative Commons licence – details of these licences and which Creative Commons licence will apply to this Work are set out in our licence referred to above.

Other than as permitted in any relevant BMJ Author's Self Archiving Policies, I confirm this Work has not been accepted for publication elsewhere, is not being considered for publication elsewhere and does not duplicate material already published. I confirm all authors consent to publication of this Work and authorise the granting of this licence.

For peer review only

The impact of new tobacco health warnings in Colombia

Sally Adams[1]; Alvaro Arturo Clavijo-Alvarez[2]; Ricardo M Tamayo[2]; Olivia M Maynard[3,4]

[1] Addiction and Mental Health Group (AIM), Department of Psychology, University of Bath, UK

[2] Departamento de Psicología, Universidad Nacional de Colombia, Colombia

[3] School of Psychological Science, University of Bristol, UK

[4] MRC Integrative Epidemiology Unit (IEU), University of Bristol, Bristol, UK

Corresponding author: Sally Adams, Addiction and Mental Health Group, Department of Psychology, University of Bath, 10 West, Bath BA2 7AY, United Kingdom. T: +44. 1225. 384004; F: +44. 1225. 386752; E:S.Adams@bath.ac.uk

Word count: 3,763

For peer review only

ABSTRACT

Objectives This study aimed to explore the impact of a new set of six pictorial warnings introduced in 2018.

Design and Setting Using a longitudinal design, we examined awareness of the new warnings among Colombian smokers across two waves of data collection.

Participants Adult smokers (≥ 18 years of age), defined as having smoked at least 100 cigarettes in their lifetime and currently smoking at least one cigarette per week participated at Wave 1, prior to the introduction of the new health warnings (*n*=1985, 72% male), and at Wave 2, twelve-months post-introduction (*n*=1572, 69% male).

Primary Outcomes At each wave we examined smokers' responses to warnings on packs (negative affect, thinking about warning messages, cognitive elaboration), attitudes towards smoking (perceived likelihood and severity of harm, self-efficacy, response-efficacy and quit intentions), knowledge of the health risks of smoking and responses to the new warnings (negative affect, believability, thinking about the harms, reactance and perceived message effectiveness).

Results Awareness of the warnings was low, with only 59% of smokers reporting having seen them at Wave 2. Between waves, we observed a reduction in negative affect towards current warnings, reduced thinking about and cognitive elaboration of the warning message and an increase in perceived severity of warnings. When asked about the six new health warnings, we found a reduction in negative affect, cognitions related to harm, believability, reactance and perceived message effectiveness between waves.

Conclusions Our data indicate a pattern of "wear-out" in warning effectiveness. Tobacco control policy should seek to improve exposure to tobacco health warnings in Colombia and to reduce wear-out effects.

Keywords: tobacco health warnings; tobacco control; policy; longitudinal survey

For peer review only

For peer review only

STRENGTHS AND LIMITATIONS

- Our study includes a large sample size, representing smokers from over 100 different areas in Colombia.
- Our work represents some of the first empirical data examining the impact of tobacco control measures such as warnings in Colombia.
- Our study is limited by the use of self-reported data on the perceived effectiveness of health warnings, where it is difficult to determine whether these responses accurately reflect behaviour
- We were unable to make a direct comparison between participants' responses between study waves, as different samples of smokers were recruited. We also observed that many participants failed our attention check, highlighting the limitation of using online self-report data.

For peer review only

INTRODUCTION

Every year, over 20,900 Colombian adults die from tobacco-related diseases and despite this, 10% of the population continue to use tobacco each day.[1] Colombia is an emerging market economy, where tobacco use is a growing public health challenge.[2] In response, Colombia has introduced tobacco control strategies including restrictions on labelling and packaging of tobacco products, smoke-free places, restrictions to advertising and sales.[3]

Warnings on tobacco products are the most cost-effective tool for educating smokers and non-smokers about the health risks of tobacco use.[4] Pictorial warnings (PWs) are more likely to be noticed than text-only warnings,[5-7] are more effective at educating smokers about the health risk of smoking, increasing cognitions about health risks,[5,8] and at increasing motivation to quit smoking,[5,7,9], whilst being less vulnerable to the effects of "wear-out".[8,10] Evidence from low- and middle-income counties indicates that PWs may be more effective in these countries, being one of the few sources of information about tobacco's risks.[4,11,12] Despite this evidence there is high variability in the implementation of warnings in low- and middle-income countries.[13]

PWs were first introduced in Colombia in 2009 and since July 2010 all tobacco products have been required by law to display PWs that cover 30% of the package and are rotated annually. These requirements fall short of the World Health Organisation Framework Convention on Tobacco Control (FCTC) recommendations that warnings cover 50% of the package and that branding is replaced by plain packaging [4]. However, the Colombian Ministry of Health has called for "local evidence" on the effectiveness of larger warnings and plain packaging, before the implementation of these tobacco control measures. In Colombia, smokers' exposure to warnings may be limited. First, implementation is likely to be slow, particularly in rural areas. Each year when new warnings are introduced, retailers have 12 months to stop selling tobacco products with older warnings (referred to here as the 'phase-in period'). Second, there is a high proportion of Colombian smokers who purchase single sticks, rather than packs, who are therefore not exposed to any warnings. Given this uncertainty over the extent to

For peer review only - http://bmjopen.bmj.com/site/about/guidelines.xhtml

which Colombian smokers are exposed to new warnings, this one-year longitudinal study explores smokers' responses to a new set of six pictorial warnings covering 30% of the pack which were introduced in Colombia in 2018.

First, we examined the rate of market saturation and the noticeability of the new warnings among smokers over the implementation period. Given that there is no requirement for retailers to stop selling cigarette packs with the old warnings during the 12-month phase-in period and that many Colombian smokers do not purchase cigarette packs (instead buying single cigarettes), we hypothesised that even a year after implementation, awareness of warnings (a proxy marker of market saturation) will be low (less than 75% of respondents will have seen the warnings). Second, we examined smokers' responses to warnings on packs (negative affect, thinking about warning measures, cognitive elaboration), attitudes towards smoking (perceived likelihood and severity of harm, self-efficacy, response-efficacy and quit intentions), knowledge of the health risks of smoking and responses to the new warnings (negative affect, believability, thinking about the harms, reactance and perceived message effectiveness) across the survey waves (baseline and 12-months post introduction). Given relatively small changes between the new and old warnings and a low rate of market saturation, we hypothesised that over our assessment period there would be either a small, or no increase in negative affect, cognitions related to smoking, knowledge regarding health risks of smoking, perceived severity and likelihood of harm from smoking and quit intentions. Third, we examined smokers' responses to each of the new warnings in terms of negative affect, believability, cognitions related to harm, avoidance, reactance and perceived message effectiveness. Due to wear-out (as a result of repeated, albeit potentially limited exposure) of the warnings, we hypothesised that each of these outcomes would be greatest at baseline as compared with 12-months post exposure. Our hypotheses, methods and analysis plan were pre-registered in our study protocol (osf.io/qbr5v).

METHODS

Design

A longitudinal online survey of Colombian smokers with two waves of data collection: one prior to the introduction of the new health warnings, and one 12-months post-introduction. The study was approved by the Universidad Nacional de Colombia, Faculty of Human Sciences Ethics Committee (reference: B.VDIECH-024-18).

Participants

We recruited adult smokers (≥ 18 years of age), defined as having smoked at least 100 cigarettes in their lifetime and currently smoking at least one cigarette per week, from the staff and students at the Universidad Nacional de Colombia and from the general public. The research team used existing mailing lists to contact members of the university across eight cities in Colombia and attended student classes to encourage students to complete the surveys. We recruited participants from the general population through social media, word of mouth and posters in public spaces.

Participants provided their email addresses at baseline. For the second wave participants who completed the baseline wave were re-contacted to ask if they would like to participate again. We also sent the survey to individuals who may not have participated in the previous waves using the methods described above. At each survey wave, participants provided their email address and could choose to enter a prize draw to win one of three iPads.

Measures and Materials

The study team translated all questionnaire measures into Spanish. A professional translator also completed a process of 'back-translation'. Following this, we discussed discrepancies and optimal wording and the original Spanish translations were changed. All wording and questions were checked by native Spanish speakers in pre-study piloting.

Health warnings

For peer review only

The six warnings introduced on 21st July 2018 depicted six different health-related consequences of tobacco smoking (miscarriage, pancreatic cancer, heart disease, death, anxiety and secondhand smoke), (see Supplementary Materials - Figure 1).

Smoking behaviour

Participants completed smoking behaviour questions to determine eligibility. Demographic measures also included questions on smoker type and smoking heaviness and the first item of the Fagerström Test for Nicotine Dependence (FTND) [14] (see Supplementary Materials – Table 1).

Awareness of new warnings

Awareness of the new pictorial warnings among Colombian smokers was assessed across two waves of data collection.

Responses to warnings seen on tobacco packs

Responses to warnings seen on packs were assessed using measures of negative affect, thinking about warning message and cognitive elaboration.

Attitudes to smoking

Attitudes to smoking were assessed using measures of perceived likelihood of harm, perceived severity of harm from smoking, self-efficacy, response efficacy and quit intentions.

Responses to new warnings

In response to viewing the six new health warnings, participants were asked to complete measures of negative affect, believability, thinking about harms, reactance, perceived message effectiveness.

For peer review only

Knowledge of health risks

We assessed knowledge of the six specific health risks presented on the new health warnings.

Procedure

The first wave of data collection occurred between 21st May and 12th July 2018, prior to 21st July 2018, the date at which new warnings could start appearing on the market. The second wave of data collection took place between 5th June and 1st August 2019. All procedures were identical in the two waves.

Participants accessed the study via Qualtrics and were first shown an information statement before completing a tick-box consent page. All text and questions were presented in Spanish. Participants completed screening questions and those who were ineligible were taken to the end of the study. Eligible participants completed demographic questions, responses to warnings on packs, knowledge of health risks and attitudes towards smoking. An attention check item was included in this section. Each of the six new warnings were presented individually on screen in a randomised order, and participants completed questions. After reading a debriefing page, participants who wished to be entered into the prize draw were directed to a second survey. Participants who participated in the first wave were re-contacted at the second wave to ask if they would like to participate again.

Data analysis

To examine market spread of new warnings over the implementation period we calculated percentages of participants in the two waves who reported being aware of seeing each of the six new warnings. In exploratory analyses we examined how warning awareness differs between smoking status (daily, weekly smokers) and smoker type (buys cigarette packs, buys singles, buys both).

To examine the impact of the new warnings, we compared responses to warnings on packs, knowledge of health risks and attitudes to smoking between Wave 1 and Wave 2 using independent samples t-tests. We ran these analyses both including and excluding participants who were defined as

For peer review only

unexposed to the new warnings at Wave 2. We defined participants as 'unexposed' if they reported not seeing (or being unsure whether they had seen) three or more of the six new warnings at Wave 2.

To examine smokers' responses to each of the new warnings, we compared negative affect, believability, thinking about the harms, reactance and perceived message effectiveness for each of the six warnings at Wave 1 and Wave 2 using independent samples t-tests.

The internal consistency of the latent constructs (e.g. negative affect, cognitive elaboration) were assessed using Cronbach's alpha. All latent constructs achieved acceptable to good internal consistency and latent variables were calculated by taking a mean of the individual items (see Supplementary Materials - Table 2).

We conducted analyses using IBM SPSS Statistics 22. The data that form the basis of the results presented here are available from the University of Bath Research Data Repository (TBC).

RESULTS

Data screening and participant characteristics

Data were examined prior to analysis and participants who completed the survey after the closing date (n=6 in Wave 1) or failed the attention check (n=1866 in Wave 1 and n=988 in Wave 2) were removed from further analyses. Participant characteristics are presented by data collection wave in Table 1.

[Insert Table 1 about here]

Awareness of new warnings

As expected, participants' awareness of new tobacco health warnings (i.e., participants reporting that they had seen the warning) increased from Wave 1 (33%) to Wave 2 (59%; see Figure 1). Note that the new warnings had not been introduced on packs by Wave 1, so the 33% who reported seeing them were mistaken. Participants mistakenly reporting that they had seen the new warnings at Wave 1 was highest

For peer review only

for the secondhand smoke (47%), miscarriage (45%) and heart disease (46%) warnings and lowest for the death (25%), pancreatic cancer (21%) and anxiety (18%) warnings.

[Insert Figure 1 about here]

Awareness of the new health warnings at Wave 2 was higher for smokers who reported buying only cigarette packs or both pack and single cigarettes, compared with those who bought only single cigarettes. Awareness of the new health warnings at Wave 2 was also higher for daily smokers compared with weekly smokers (see Supplementary Materials Figures 2 and 3).

Responses to warnings seen on tobacco packs

As shown in Table 2, there was evidence of a decrease in negative affect, thinking about warning messages and cognitive elaboration between Wave 1 and 2. These findings did not differ when including only participants defined as 'exposed' at Wave 2.

Attitudes to smoking

There was strong evidence of an increase in perceived severity between Wave 1 and 2 (Table 2), although this difference was not evidence after including only participants defined as exposed to the warnings at Wave 2. The analysis including only participants exposed at Wave 2 also indicated strong evidence of a decrease in self-efficacy and quit intention between waves. There was no evidence for any other differences.

Knowledge of health risks

There was strong evidence of an increase in mean knowledge of health risks between waves across all warnings, when including only participants defined as exposed at Wave 2 (see Table 2). For individual

For peer review only - http://bmjopen.bmj.com/site/about/guidelines.xhtml

warnings, there was evidence of a small, but statistically meaningful increase in knowledge for the health risks “Smoking causes pancreatic cancer” and “Smoking causes anxiety” between waves. The analysis including only participants exposed to the warnings at Wave 2 indicated that there was also evidence of an increase between waves in knowledge for the health risk “Smoking while pregnant could cause you to lose your baby”. These data were highly skewed, however, with high rates of participants giving scores of either 0 (strongly disagree) or 100 (strongly agree) for each knowledge question (See Table 3).

[Insert Tables 2 and 3 about here]

For peer review only

Responses to new warnings

Differences in responses to the six new health warnings are shown in Table 4.

For the health warning “Miscarriage” there was strong evidence of a decrease in negative affect, cognitions related to harm, believability and perceived message effectiveness between waves. For the health warning “Pancreatic cancer” there was strong evidence of a decrease in cognitions related to harm and reactance and an increase in believability between waves. For the health warning “Anxiety” there was no evidence of any differences in response to the warning. For the health warning “Heart disease” there was strong evidence of a decrease in negative affect, cognitions related to harm and perceived message effectiveness between wavs. For the health warning “Second-hand smoke” there was strong evidence of a decrease in negative affect, cognitions related to harm, believability and perceived message effectiveness between waves. Finally, for the health warning “Death” there was strong evidence of a decrease in negative affect, cognitions related to harm and perceived message effectiveness between waves. There was no evidence for any other differences.

[Insert Table 4 about here]

DISCUSSION

Every year since 2010, Colombian smokers have been faced with new health warnings covering 30% of their cigarette packs. Using a longitudinal design, we examined how smokers responded to the new set of warnings introduced in 2018 and find that they had relatively limited impacts on knowledge of risk and attitudes towards smoking. We do however find some interesting differences between the warnings themselves, and together these findings have important implications for health warning design and implementation in Colombia and worldwide.

For peer review only

As hypothesised, we found that awareness of the warnings was low. Across the six warnings, the average percentage of participants reporting having seen them at Wave 2 was 59%. This is particularly low, given that even at Wave 1, 33% of participants incorrectly reported having seen the warnings. Given this low level of awareness, it is unsurprising that we see either no changes, or relatively small changes in our measures of responses to warnings on packs, overall knowledge of health risks and attitudes to smoking between Waves 1 and 2 – these are outcomes we would expect to improve, had the new warnings made an impact on smokers. Although the differences were small, contrary to our hypothesis, participants reported *lower* negative affect, thinking about the warnings and cognitive elaboration of the warnings on their cigarette packs at Wave 2. Even when we only include those participants we defined as 'exposed' to the new warnings at Wave 2, these unexpected effects persisted. We saw little to no change in attitudes to smoking between waves, although there was some evidence that participants at Wave 2 thought that the severity of smoking harms were greater, and they had greater knowledge of the health risks of smoking. However, again, contrary to expectations, when we only examined these effects among participants who we defined as exposed to the new warnings at Wave 2, self-efficacy and quit intentions were lower at Wave 2.

Together, these findings suggest that the new warnings had relatively little impact on smokers. We consider there to be a number of complementary explanations for this. First, although Wave 2 was conducted 12 months after the new warnings were permitted for sale, it is likely that they did not become commonplace for some time after that, as there is no legal requirement for retailers to stop selling packs with older tobacco warnings until 12-months after the implementation period. It is possible that this slow phase-in diminished warnings' impact and meant that even a year after they (in theory) started being available on packs for sale, few participants recalled having seen them. Second, low awareness of warnings is also

For peer review only

likely related to the extent to which smokers engage with cigarette packs. Despite being prohibited by law, many Colombian smokers purchase single cigarettes, [15] and therefore may not be exposed to health warnings at all. Indeed, we find that awareness of the new warnings is greatest for those who smoke daily and those who buy packs.

We do however find some interesting differences between the warnings themselves. As might be expected, we see the highest levels of participants incorrectly reporting at Wave 1 that they had seen the new warnings for those with themes used in previous years (‘miscarriage’, ‘heart disease’, ‘secondhand smoke’ were used most recently in 2017 and ‘death’ was used in 2015; see Supplementary Materials -Table 3). For these four ‘familiar’ warnings (similar theme, but different wording and pictorial), we see high levels of believability and knowledge of these health risks at both waves and only a small proportion of participants reporting that they ‘strongly disagreed’ that smoking caused these health risks.

However, for all four of these familiar warnings, we observe reductions between waves in almost all specific responses to them (e.g., perceived message effectiveness and negative affect). These data provide support for previous work which has observed a declining impact of warnings (i.e., “wear out”) over time. [7,16-19] In contrast to previous work, we found no evidence that cognitive elaboration of risks (e.g., thinking about smoking health risks and quitting) is more resilient to wear-out than other responses towards warnings,[10] or that believability is sustained over time. [20-22] Our data suggest that wear-out can occur within just 12-months. It has previously been suggested that wear-out may be reduced by the introduction of larger pictorial health warnings. [20,23] Currently, warning labels in Colombia cover 30% of the pack, with the FCTC recommending that warning labels should cover at least 50%. Although our study was not designed to assess this, the current warning size in Colombia may limit the effectiveness of health warnings and facilitate wear-out, especially on smaller packs of 10 cigarettes.

For peer review only - http://bmjopen.bmj.com/site/about/guidelines.xhtml

There are two warning themes which were new on Colombian cigarette packs in 2018, ‘pancreatic cancer’ and ‘anxiety’. As expected, for these novel warnings, we saw a much smaller percentage of participants incorrectly reporting that they had seen them at Wave 1 than the four familiar warnings. However, interestingly, at Wave 2, we saw divergent levels of awareness for these two warnings. The largest percentage of participants reported having seen the pancreatic cancer warning of all warnings at Wave 2, but the lowest percentage of all participants reported having seen the anxiety warning. We suggest this may be due to the pictorial used, with the pancreatic cancer warning displaying a close-up of a yellow eye, which is arguably novel and memorable, while the anxiety warning depicted a man pulling at his hair. Previous research has suggested that more “severe” or grotesque warnings increase smokers’ intentions to quit and are rated as more believable and effective than less severe warnings. [24,25]

Knowledge of the risk of smoking for both pancreatic cancer and anxiety was low at Wave 1 and at this time-point we saw the highest percentages of participants reporting that they ‘strongly disagreed’ that smoking caused these conditions. The inclusion of these new warning messages reflects the priority for the Colombian Ministry of Health to inform the public about “lesser known” smoking health harms. We saw increased knowledge of these two health risks at Wave 2, and fewer participants selecting ‘strongly disagree’ particularly for anxiety, although knowledge was still lower than for most other health risks. Despite this, these increases suggest that novel warnings can impact smokers’ knowledge of smoking risks. We also saw that the anxiety warning had the lowest response scores at both waves compared with all other warnings (particularly negative affect, believability, perceived message effectiveness and cognitions related to harm). We suggest that these low scores, combined with low ‘knowledge’ scores, are in line with the inconsistent longitudinal evidence regarding the relationship between smoking and anxiety,[26] and evidence from

For peer review only

Mendelian randomisation studies which find no causal relationship between smoking and the onset of anxiety.[27] We suggest that it is important to consider the veracity of the health risk claims depicted in health warnings and we raise the important question of whether including health risks which are not supported by the evidence might reduce the overall impact of warnings.

Warnings have repeatedly been shown to be a cost-effective tool for communicating the health risks of smoking to smokers' and non-smokers.[4] For low- and middle-income countries, warnings are proposed to be even more effective.[4] However, for this to occur, smokers must be exposed to warnings and increased noticeability may prevent wear-out effects.[20] Our data suggest that strategies for increasing exposure to warnings are required in Colombia, particularly as a large proportion of smokers purchase single cigarettes rather than packs. One method for achieving this is to include health warning messages on individual cigarette sticks and emerging evidence indicates that this represents a novel and effective method for reducing tobacco use.[28-30]

Our study has numerous strengths, including a large sample size, representing smokers from over 100 different areas in Colombia. Additionally, our work represents some of the first empirical data examining the impact of tobacco control measures such as warnings in Colombia. However, our findings should also be considered in light of the following limitations. Our study is limited by the use of self-reported data on the perceived effectiveness of health warnings, where it is difficult to determine whether these responses accurately reflect behaviour. We were unable to make a direct comparison between participants' responses between study waves, as different samples of smokers were recruited. We also observed that many participants failed our attention check, highlighting the limitation of using online self-report data.

For peer review only

CONCLUSIONS

Future tobacco control policy should seek to improve the noticeability of and exposure to tobacco health warnings in Colombia and to reduce the impact of wear-out effects. Updating and rotating tobacco warnings is a major challenge facing regulators in maintaining the effectiveness of health messages.[31] Rotation should include considering what similar warning themes have been used in previous years as well as ensuring that new warnings are well supported by scientific evidence. It is also likely that larger health warnings may prevent wear-out effects. Tobacco control policy may also seek to implement a requirement of retailers to stop selling cigarette packs with older warnings to reduce the phase-in period for new warnings and improve the availability and impact of new warnings.

For peer review only

CONTRIBUTORSHIP

Conception and design of the work: SA, RMT, AACA, OMM. Analysis: SA and OMM. Interpretation of data for the work: all authors. Drafting the work: SA and OMM. Revising it critically: all authors. Final approval of the version to be published: all authors.

FUNDING

This research was funded by an ESRC New Investigator's Award, awarded to Dr Olivia Maynard (ES/R003424/1) and a University of Bristol Global Challenges Research Ad Hoc Fund.

COMPETING INTERESTS

RMTO and AACA were previously hired by the Colombian Ministry of Health to design the six warnings used here. The Ministry of Health had no role in the design of the study.

PATIENT AND PUBLIC INVOLVEMENT

It was not appropriate or possible to involve patients or the public in the design, or conduct, or reporting, or dissemination plans of our research

DATA AVAILABILITY

The data will be made available for sharing via a University of Bristol online data repository

For peer review only

TABLES

Table 1. Participant characteristics by data collection wave

	**Wave 1** *n* = 1985	**Wave 2** *n* = 1572	***p* - value**
Sex (% male)	72	69	N/A
Age (years)	26 (9)	26 (8)	0.03
Location (% Bogota)	49	67	< 0.001
Smoking frequency			
Daily smokers (%)	65	62	0.06
Cigarettes per day (daily smokers)	7 (6)	7 (5)	N/A
Weekly smokers (%)	35	38	0.06
Cigarettes per week (weekly smokers)	10 (10)	9 (9)	0.09
How many days a week do you smoke? (weekly smokers)	3 (1)	3 (1)	0.42
Cigarettes per day (weekly smokers)	3 (3)	3 (3)	0.38
Type of packs bought			
Buys cigarette packs (%)	38	31	< 0.001
Buys single cigarettes (%)	22	26	N/A
Buys both packs and single cigarettes (%)	39	41	0.21
Dependence			
Smokes within first 60 minutes of waking (%)	27	24	0.02

Note. Values are %, except for age, cigarettes per day, cigarettes per week, smoking days per week -mean (standard deviation). P-values represent differences in variables between waves (t-tests/chi-square).

For peer review only

Table 2. Responses to tobacco health warnings and attitudes to smoking by data collection wave (for all participants and excluding participants unexposed to new warnings at Wave 2).

		**Wave 1 vs Wave 2 - including all participants at Wave 2**			**Wave 1 vs Wave 2 - only including participants exposed to new warnings at Wave 2**				
	**Wave 1**	**Wave 2**	***t***	***df***[b]	***p* - value**	**Wave 2**	***t***	***df***[b]	***p* - value**
	(*n* = 1985)	(*n* = 1572)				(n = 910)			
Responses to warnings on packs									
Negative affect	41(27)	38 (26)	3.03	3411	<0.001	38 (26)	3.35	2893	<0.001
Thinking about warning messages	31 (26)	28 (25)	3.28	3454	<0.001	27 (25)	3.28	1880	<0.001
Cognitive elaboration	46 (31)	42 (29)	3.54	3432	< 0.001	42 (29)	3.59	1867	< 0.001
Knowledge of health risks (overall)	73 (17)	74 (16)	-1.78	3443	0.08	76 (17)	-3.91	2893	< 0.001
Lose your baby[a]	85 (22)	85 (21)	-0.59	3470	0.56	87 (20)	-2.24	1979	0.03
Smoking causes pancreatic cancer	68 (29)	70 (27)	-2.2	3555	0.03	72 (28)	-3.53	2893	< 0.001
Secondhand smoke is deadly	74 (27)	73 (26)	1.2	3555	0.23	74 (26)	0.41	2893	0.68
Smoking damages your heart	83 (21)	83 (20)	-0.03	3555	0.98	84 (20)	-0.9	2893	0.37
Smoking causes anxiety	60 (36)	64 (34)	-3.41	3471	<0.001	67 (33)	-5.67	1948	< 0.001
Smoking causes a slow and painful death	68 (30)	68 (28)	-0.59	3460	0.56	70 (28)	-1.93	1873	0.05
Attitudes to smoking									
Perceived likelihood of harm	78 (19)	77 (17)	0.61	3467	0.54	78 (17)	-0.3	1887	0.76
Perceived severity of smoking harms	87 (17)	89 (15)	-3.97	3501	< 0.001	88 (16)	-1.73	1815	0.08
Self-efficacy	69 (32)	67 (32)	1.65	3555	0.1	64 (33)	3.73	1708	< 0.001
Response-efficacy	83 (24)	84 (22)	-0.54	3487	0.59	84 (22)	-0.32	1899	0.75
Quit intention	60 (30)	59 (30)	1.61	3554	0.11	56 (31)	3.9	2892	< 0.001

For peer review only

Note. Vales are mean (standard deviation), *t*-score, degrees of freedom and *p*-values

[a] Full wording is 'Smoking while pregnant could cause you to lose your baby'

[b] Degrees of freedom differ according to equality of variances of variables

For peer review only

Table 3. Knowledge of health risks (percentage of participants giving a score of 0 or 100) by data collection wave (for all participants and excluding participants unexposed to new warnings at Wave 2).

			**Wave 1 vs Wave 2 including all participants at Wave 2**		**Wave 1 vs Wave 2 - only including participants exposed to new warnings at Wave 2**	
	**Wave 1**		**Wave 2**		**Wave 2**	
	(*n* = 1985)		(*n* = 1572)		(n = 910)	
	% giving a score of 0	% giving a score of 100	% giving a score of 0	% giving a score of 100	% giving a score of 0	% giving a score of 100
**Warning 1 "Pancreatic cancer"**	4	24	3	25	3	28
**Warning 2 "Miscarriage"**	1	50	1	48	1	51
**Warning 3 "Death"**	5	25	3	23	3	25
**Warning 4 "Secondhand smoke"**	2	31	1	27	1	30
**Warning 5 "Heart disease"**	1	41	1	39	1	42
**Warning 6 "Anxiety"**	11	25	7	25	6	28

Note. Values are % of participants giving a score of 0 ‘strongly disagree’ or 100 “strongly agree” for the six new warnings

For peer review only

Table 4. Responses to the new tobacco health warnings by data collection wave

	**Wave 1**	**Wave 2**	***t***	***df*** [a]	***p* - value**
	(*n* = 1985)	(*n* = 1572)			
**Warning 1 "Pancreatic cancer"**					
Negative affect	47 (30)	45 (28)	1.79	3093	0.07
Cognitions related to harm	59 (32)	57 (32)	2.55	3134	0.01
Believability	57 (32)	60 (31)	- 2.97	3076	< 0.001
Reactance	41 (25)	39 (25)	2.52	3134	0.01
Perceived message effectiveness	48 (27)	47 (26)	1.52	3134	0.13
**Warning 2 "Miscarriage"**					
Negative affect	50 (30)	44 (29)	5.57	3128	<0.001
Cognitions related to harm	59 (34)	51 (35)	6.16	2984	<0.001
Believability	67 (31)	64 (31)	2.92	3128	< 0.001
Reactance	43 (27)	42 (27)	0.84	3128	0.4
Perceived message effectiveness	51 (27)	47 (27)	4.1	3128	<0.001
**Warning 3 "Death"**					
Negative affect	55 (30)	51 (29)	3.52	3136	<0.001
Cognitions related to harm	67 (31)	64 (31)	2.75	3136	0.01
Believability	68 (30)	66 (30)	1.5	3136	0.13
Reactance	42 (27)	41 (25)	0.4	3096	0.69
Perceived message effectiveness	54 (26)	52 (27)	2.31	3136	0.02
**Warning 4 "Secondhand smoke"**					
Negative affect	46 (29)	42 (28)	3.52	3123	<0.001
Cognitions related to harm	61 (33)	55 (33)	4.95	3123	<0.001
Believability	69 (30)	66 (30)	2.19	3123	0.03
Reactance	40 (25)	40 (25)	- 0.19	3123	0.85
Perceived message effectiveness	48 (26)	45 (26)	3.39	3123	< 0.001
**Warning 5 "Heart disease"**					
Negative affect	49 (30)	45 (28)	3.33	3068	< 0.001
Cognitions related to harm	64 (31)	60 (31)	3.41	3112	< 0.001
Believability	66 (30)	65 (30)	1.6	3112	0.11
Reactance	38 (25)	37 (24)	1.51	3058	0.13

For peer review only

Perceived message effectiveness	49 (26)	46 (25)	3.47	3112	< 0.001
**Warning 6 "Anxiety"**					
Negative affect	30 (28)	29 (26)	1.2	3089	0.23
Cognitions related to harm	42 (35)	41 (33)	1.04	3084	0.3
Believability	51 (34)	53 (34)	-1.4	3131	0.16
Reactance	41 (25)	40 (25)	1.14	3131	0.26
Perceived message effectiveness	36 (25)	35 (25)	0.93	3131	0.35

Note. Values are mean (standard deviation), *t*-score, degrees of freedom and *p*-values

[a] Degrees of freedom differ according to equality of variances of variables

For peer review only

REFERENCES

1 American Cancer Society (2021, April 28). Tobacco Atlas: Colombia https://tobaccoatlas.org/country/colombia/

2 Posada-Villa, J., Cheng, H. G., Martins, S. S., Storr, C. L., Aguilar-Gaxiola, S. A., & Anthony, J. C. (2011). The tobacco dependence dimension in Colombia. *Revista panamericana de salud publica = Pan American journal of public health*, *29*(1), 52–56.

3 Campaign for Tobacco Free Kids (2021, April 28) Legislation by Country: Colombia https://www.tobaccocontrollaws.org/legislation/country/colombia/pl-health-warnings

4 Fong, G. T., Hammond, D., & Hitchman, S. C. (2009). The impact of pictures on the effectiveness of tobacco warnings. *Bulletin of the World Health Organization*, *87*(8), 640–643. https://doi.org/10.2471/blt.09.069575

5 Hammond, D., Fong, G. T., McNeill, A., Borland, R., & Cummings, K. M. (2006). Effectiveness of cigarette warning labels in informing smokers about the risks of smoking: findings from the International Tobacco Control (ITC) Four Country Survey. *Tobacco control*, *15 Suppl 3*(Suppl 3), iii19–iii25. https://doi.org/10.1136/tc.2005.012294

6 Thrasher, J. F., Hammond, D., Fong, G. T., & Arillo-Santillán, E. (2007). Smokers' reactions to cigarette package warnings with graphic imagery and with only text: a comparison between Mexico and Canada. *Salud publica de Mexico*, *49 Suppl 2*, S233–S240. https://doi.org/10.1590/s0036-36342007000800013

7 Borland, R., Wilson, N., Fong, G. T., Hammond, D., Cummings, K. M., Yong, H. H., Hosking, W., Hastings, G., Thrasher, J., & McNeill, A. (2009a). Impact of graphic and text warnings on cigarette packs: findings from four countries over five years. *Tobacco control*, *18*(5), 358–364. https://doi.org/10.1136/tc.2008.028043

8 Li, J., & Grigg, M. (2009). New Zealand: new graphic warnings encourage registrations with the quitline. *Tobacco control*, *18*(1), 72. https://doi.org/10.1136/tc.2008.027649

9 Borland, R., Yong, H. H., Wilson, N., Fong, G. T., Hammond, D., Cummings, K. M., Hosking, W., & McNeill, A. (2009b). How reactions to cigarette packet health warnings influence quitting: findings from the ITC Four-Country survey. *Addiction (Abingdon, England)*, *104*(4), 669–675. https://doi.org/10.1111/j.1360-0443.2009.02508.x

10 Hammond, D., Fong, G. T., Borland, R., Cummings, K. M., McNeill, A., & Driezen, P. (2007). Text and graphic warnings on cigarette packages: findings from the international tobacco control four country study. *American journal of preventive medicine*, *32*(3), 202–209. https://doi.org/10.1016/j.amepre.2006.11.011

11 Swayampakala, K., Thrasher, J. F., Yong, H. H., Nagelhout, G. E., Li, L., Borland, R., Hammond, D., O'Connor, R. J., & Hardin, J. W. (2018). Over-Time Impacts of Pictorial Health Warning Labels and their Differences across Smoker Subgroups: Results from Adult Smokers in Canada and Australia. *Nicotine & tobacco research: official journal of the Society for Research on Nicotine and Tobacco*, *20*(7), 888–896. https://doi.org/10.1093/ntr/ntx134

For peer review only

12 FCTC (2009) article 11: tobacco warning labels: evidence and recommendations from the ITC project Waterloo, ON: International Tobacco Control Policy Evaluation Project. http://www.itcproject.org

13 Wipfli, H. L., Fujimoto, K., & Valente, T. W. (2010). Global tobacco control diffusion: the case of the framework convention on tobacco control. *American journal of public health*, *100*(7), 1260–1266. https://doi.org/10.2105/AJPH.2009.167833

14 Heatherton, T. F., Kozlowski, L. T., Frecker, R. C., & Fagerström, K. O. (1991). The Fagerström Test for Nicotine Dependence: a revision of the Fagerström Tolerance Questionnaire. *British journal of addiction*, *86*(9), 1119–1127. https://doi.org/10.1111/j.1360-0443.1991.tb01879.x

15 Maldonado, N., Llorente, B., Escobar, D., et al (2020). Smoke signals: monitoring illicit cigarettes and smoking behaviour in Colombia to support tobacco taxes. *Tobacco Control,* 29, 243-248.

16 Woelbert, E., d'Hombres, B. (2019). Pictorial health warnings and wear-out effects: evidence from a web experiment in 10 European countries. *Tobacco Control,* 28, 71-76.

17 Environics Research Group (1999) Canadian adult and youth opinions on the sizing of health warning messages. Ottawa, Canada.

18 Environics Research Group (2000) Testing new health warning messages for cigarette packages: A summary of three phases of focus group research: Final report. Ottawa, Canada.

19 Brubaker, R. G., & Mitby, S. K. (1990). Health-risk warning labels on smokeless tobacco products: are they effective?. *Addictive behaviors*, *15*(2), 115–118. https://doi.org/10.1016/0306-4603(90)90014-o

20 Anshari, D., Yong, H., Borland, R., et al (2018). Which type of tobacco product warning imagery is more effective and sustainable over time? A longitudinal assessment of smokers in Canada, Australia and Mexico. *BMJ Open, 8*. doi: 10.1136/bmjopen-2018-021983

21 Shanahan, P., & Elliott, D. (2008) Evaluation of the Effectiveness of the Graphic Health Warnings on Tobacco Product Packaging. Canberra, Australian Government Department of Health and Aging. Evaluation of effectiveness of graphic health warnings on tobacco product packaging

22 Environics Research Group (2002) The health effects of tobacco and health warning messages on cigarette packages: Survey of adults and adult smokers. Toronto, Canada.

23 Li, L., Borland, R., Yong, H., Cummings, K. M., Thrasher, J. F., Hitchman, S. C., Fong, G. T., Hammond, D., & Bansal-Travers, M. (2015). Longer term impact of cigarette package warnings in Australia compared with the United Kingdom and Canada. *Health education research*, *30*(1), 67–80. https://doi.org/10.1093/her/cyu074

For peer review only

24 Maynard, O.M., Gove, H., Skinner, A.L. *et al.* (2018) Severity and susceptibility: measuring the perceived effectiveness and believability of tobacco health warnings. *BMC Public Health* 18**,** 468. https://doi.org/10.1186/s12889-018-5385-x

25 Kees, J., Burton, S., Andrews, J. C., & Kozup, J. (2010). Understanding how Graphic Pictorial Warnings Work on Cigarette Packaging. *Journal of Public Policy & Marketing*, *29*(2), 265–276. https://doi.org/10.1509/jppm.29.2.265

26 Fluharty, M., Taylor, A. E., Grabski, M., & Munafò, M. R. (2017). The Association of Cigarette Smoking with Depression and Anxiety: A Systematic Review. *Nicotine & tobacco research: official journal of the Society for Research on Nicotine and Tobacco*, *19*(1), 3–13. https://doi.org/10.1093/ntr/ntw140

27 Taylor, A. E., Fluharty, M. E., Bjørngaard, J. H., Gabrielsen, M. E., Skorpen, F., Marioni, R. E., Campbell, A., Engmann, J., Mirza, S. S., Loukola, A., Laatikainen, T., Partonen, T., Kaakinen, M., Ducci, F., Cavadino, A., Husemoen, L., Ahluwalia, T. S., Jacobsen, R. K., Skaaby, T., Ebstrup, J. F., … Munafò, M. R. (2014). Investigating the possible causal association of smoking with depression and anxiety using Mendelian randomisation meta-analysis: the CARTA consortium. *BMJ open*, *4*(10), e006141. https://doi.org/10.1136/bmjopen-2014-006141

28 Drovandi, A., Teague, P.A., Glass, B., Malau-Aduli, B. (2019) Do health warnings on cigarette sticks dissuade smokers and non-smokers? A focus group and interview study of Australian university students. *Psychol Res Behav Manag*. 12:361-373 https://doi.org/10.2147/PRBM.S193754

For peer review only

29 Moodie, C.S., Hiscock, R,. Thrasher, J. *et al.* (2018) Perceptions of cigarette pack inserts promoting cessation and dissuasive cigarettes among young adult smokers in the UK: a cross-sectional online survey. *BMJ Open* 8:e019662. doi: 10.1136/bmjopen-2017-019662

30 Moodie, C., Gendall, P., Hoek, J., MacKintosh, A. M., Best, C., & Murray, S. (2019). The Response of Young Adult Smokers and Nonsmokers in the United Kingdom to Dissuasive Cigarettes: An Online Survey. *Nicotine & tobacco research : official journal of the Society for Research on Nicotine and Tobacco*, *21*(2), 227–233. https://doi.org/10.1093/ntr/ntx261

31 Hammond, D. & Reid, J.L. (2012) Health warnings on tobacco products: International practices. Salud publica de Mexico, *54* (3), 270-80. Doi:10.1590/S0036-36342012000300009

Figure 1. Percentage of participants who report being aware of having seen the new warnings at Wave 1 and 2

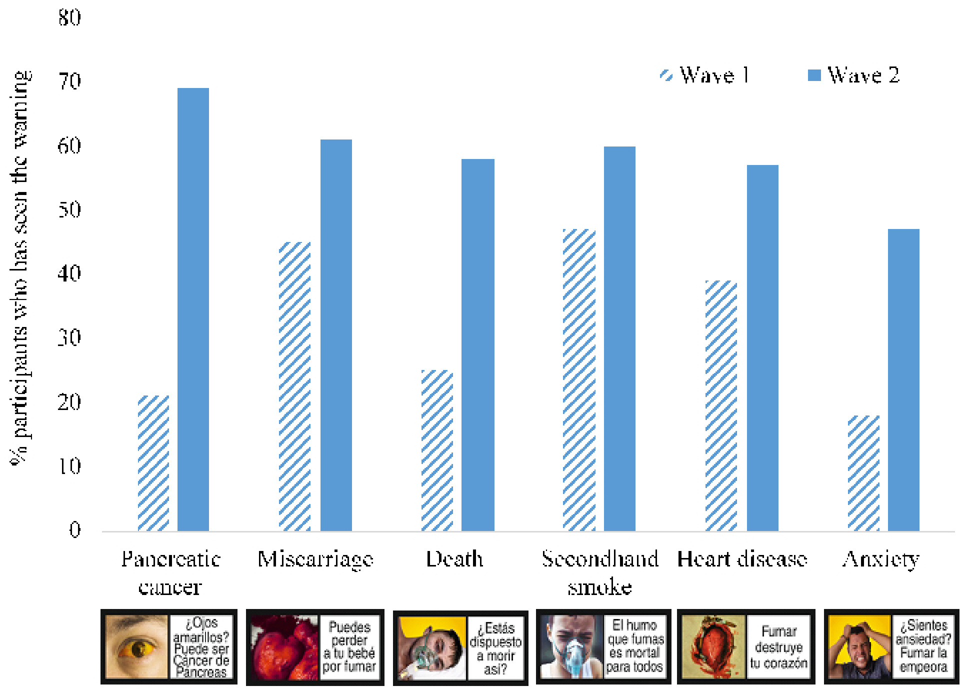

185x153mm (96 x 96 DPI)

Supplementary Materials Table 1. Survey Questions

Variable	Items	Response
Smoking behaviour - eligibility	Have you smoked at least 100 cigarettes in your lifetime?	1 = Yes<br>2 = No
	How often do you smoke cigarettes?	1 = Every day<br>2 = Every week<br>3 = Less than every week<br>4 = Not al all
Smoking heaviness – daily smokers	Approximately how many cigarettes do you smoke per day?	Provide a single number
Smoking heaviness -non-daily smokers	Approximately how many days a week do you smoke	
	On the days that you do smoke, approximately how many cigarettes do you smoke	
	Approximately how many cigarettes do you smoke per week?	
Fagerström Test for Nicotine Dependence (FTND)	How soon after you wake do you smoke your first cigarette?	1 = Within 5 minutes<br>2 = 6 to 30 minutes<br>3 = 31 to 60 minutes<br>4 = After 60 minutes
Awareness of new warnings	Have you seen this health warning on a cigarette pack?	1= Yes<br>2 = No<br>3 = Unsure
Responses to warnings seen on tobacco packs		
Negative affect	In the last week, the warning on my cigarette pack made me feel...	
	…scared	0 = Strongly disagree<br>25 = Somewhat disagree<br>50 = Neither agree nor disagree<br>75 = Somewhat agree<br>100 = Strongly agree
	…regretful	
	…disgusted	

Thinking about the warning message	When I notice my cigarette pack, I think about the message that the warning conveys	0 = Strongly disagree<br>25 = Somewhat disagree<br>50 = Neither agree nor disagree<br>75 = Somewhat agree<br>100 = Strongly agree
	When my cigarette pack is not<br>in sight, I think about the message that the warning conveys	
Attitudes towards smoking		
Perceived<br>likelihood of harm	What is the chance that you will one day get the following diseases if you continue to smoke cigarettes?	
	…heart disease?	0 = No chance<br>25 = Low chance<br>50 = Moderate chance<br>75 = High chance<br>100 = Certain
	…cancer?	
	…a permanent breathing problem?	
Perceived<br>severity of harm<br>from smoking	How much would getting the following diseases because of smoking affect your life?	
	…heart disease?	0 = No chance<br>25 = Low chance<br>50 = Moderate chance<br>75 = High chance<br>100 = Certain
	…cancer?	
	…a permanent breathing problem?	
Self-efficacy	I see myself as being capable of quitting smoking in the next 2 months if I wanted to	0 = Strongly disagree<br>25 = Somewhat disagree<br>50 = Neither agree nor disagree<br>75 = Somewhat agree<br>100 = Strongly agree
Response-efficacy	Quitting smoking would lower my chances of getting a<br>disease related<br>to smoking	

Quit intentions	Are you planning to quit smoking within the next 6 months?	0 = Very unlikely<br>25 = Unlikely<br>50 = Maybe, maybe not<br>75 = Likely<br>100 = Very likely
Responses to new warnings		
Negative affect	This warning makes me feel…	
	…scared	0 = Strongly disagree<br>25 = Somewhat disagree<br>50 = Neither agree nor disagree<br>75 = Somewhat agree<br>100 = Strongly agree
	…regretful	
	…disgusted	
Believability	This warning is believable	0 = Strongly disagree<br>25 = Somewhat disagree<br>50 = Neither agree nor disagree<br>75 = Somewhat agree<br>100 = Strongly agree
Thinking about harms	This warning makes me think about the health problems caused by smoking	0 = Strongly disagree<br>25 = Somewhat disagree<br>50 = Neither agree nor disagree<br>75 = Somewhat agree<br>100 = Strongly agree
Reactance	This warning is trying to manipulate me	0 = Strongly disagree<br>25 = Somewhat disagree<br>50 = Neither agree nor disagree<br>75 = Somewhat agree<br>100 = Strongly agree
	The health effect of this warning is overblown	
	This warning annoys me	
Perceived message effectiveness	This warning discourages me from wanting to smoke	0 = Strongly disagree<br>25 = Somewhat disagree<br>50 = Neither agree nor disagree<br>75 = Somewhat agree<br>100 = Strongly agree
	This warning makes smoking seem unpleasant to me	
	This warning makes me concerned about the health effects of smoking	
	If I saw this warning on my	

For peer review only - http://bmjopen.bmj.com/site/about/guidelines.xhtml

	cigarette pack, I would...	
	...try to avoid thinking about it	
	…try to avoid looking at it	
	…keep the pack out of sight to avoid looking at the warning	
Knowledge of health risks	Say how much you agree or disagree with each statement below:	0 = Strongly disagree<br>25 = Somewhat disagree<br>50 = Neither agree nor disagree<br>75 = Somewhat agree<br>100 = Strongly agree
	Smoking while pregnant could cause you to lose your baby	
	Smoking causes pancreatic cancer	
	Secondhand smoke is deadly	
	Smoking damages your heart	
	Smoking causes anxiety	
	Smoking causes a slow and painful death	

For peer review only

Supplementary Material – Table 2. Internal consistency of latent constructs

**Latent construct**	**Number of items**	**α**	***r***
Responses to warnings on packs			
Negative affect	3 items	0.79	0.49 – 0.69
Thinking about warning message	2 items	0.66	0.5
Cognitive elaboration	2 items	0.8	0.66
Attitudes to smoking			
Perceived likelihood of harm	3 items	0.84	0.61-0.65
Perceived severity	3 items	0.87	0.66-0.71
Responses to new warnings			
**Warning 1 "Pancreatic cancer"**			
Negative affect	3 items	0.83	0.54 – 0.77
Reactance	3 items	0.68	0.39 – 0.45
Perceived effectiveness	6 items	0.87	0.34 – 0.82
**Warning 2 "Miscarriage"**			
Negative affect	3 items	0.77	0.38 – 0.79
Reactance	3 items	0.69	0.41 – 0.44
Perceived effectiveness	6 items	0.86	0.28 – 0.75
**Warning 3 "Death"**			
Negative affect	3 items	0.8	0.46 – 0.77
Reactance	3 items	0.69	0.43 – 0.44
Perceived effectiveness	6 items	0.86	0.33 – 0.82
**Warning 4 "Secondhand Smoke"**			
Negative affect	3 items	0.81	0.49 – 0.67
Reactance	3 items	0.67	0.37 – 0.44
Perceived effectiveness	6 items	0.86	0.31 – 0.80
**Warning 5 "Heart disease"**			
Negative affect	3 items	0.83	0.53 – 0.80
Reactance	3 items	0.68	0.40 – 0.44
Perceived effectiveness	6 items	0.86	0.33 – 0.80
**Warning 6 "Anxiety"**			
Negative affect	3 items	0.87	0.63 – 0.76
Reactance	3 items	0.64	0.33 – 0.43
Perceived effectiveness	6 items	0.87	0.34 – 0.85

For peer review only

Note. The internal consistency of latent constructs assessed using Cronbach's alpha and associations between the constructs using bivariate correlations.

For peer review only

Supplementary Materials Table 3. – Presence of warning theme between 2010 and 2018.

**Warning theme**	**2010**	**2011**	**2012**	**2013**	**2014**	**2015**	**2016**	**2017**	**2018**
Death							X		X
Secondhand smoke		X			X			X	X
Anxiety									X
Miscarriage		X		X			X	X	X
Heart attack / heart disease					X		X	X	X
Pancreatic cancer									X
Mouth cancer and bad breath	X								
Kidney cancer				X				X	
Breast cancer				X					
Leukemia					X				
Bladder cancer					X				
Laryngeal cancer							X		
Cancer							X		
Damaged arteries					X			X	
Thrombosis					X				
Vascular problems / disease				X					
Cerebral stroke		X							
Lung disease		X					X		
Eye problems / disease		X							
Bone damage				X					
Sexual impotence	X	X		X					
Aging	X								

Data are taken from https://www.tobaccocontrollaws.org/legislation/country/colombia/laws. Data not available for 2012 or 2015.

All warnings are available from the Colombian Ministry of Health and Social Protection.

Colombia. Ministerio de Salud y Protección Social. (2020). Advertencias sanitarias productos de tabaco y derivados 2010 - 2021. [Piezas publicitarias;] Retrieved From: https://www.minsalud.gov.co/sites/rid/Lists/BibliotecaDigital/RIDE/VS/PP/ENT/advertencias-sanitarias-todas-vigencias.zip

For peer review only

Supplementary Material - Figure 1. Six new tobacco health warnings introduced in Colombia July 2018

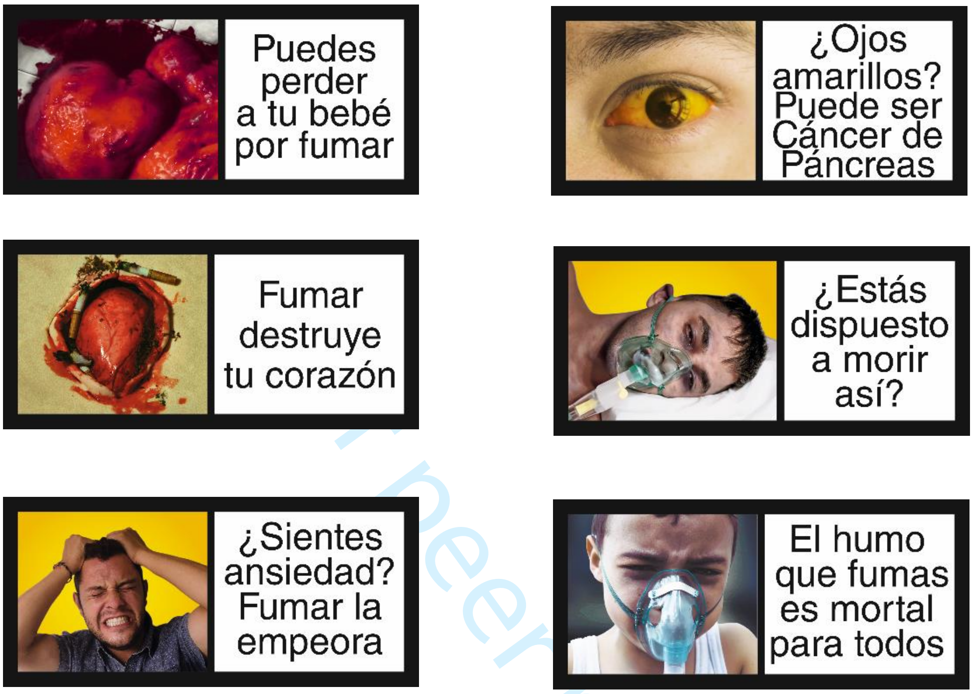

Health warning topics – clockwise *"You can lose your baby by smoking", "Yellow eyes? It could be pancreatic cancer", "Smoking destroys your heart", "Are you willing to die like this?", "Do you feel anxiety? Smoking makes it worse"* and *"The smoke you smoke is deadly for everyone".*

For peer review only

Supplementary Material – Figure 2. Percentage of participants who report having seen the new tobacco health warnings by data collection wave and smoker type (i.e., buys cigarette packs, buys packs and singles, buys singles).

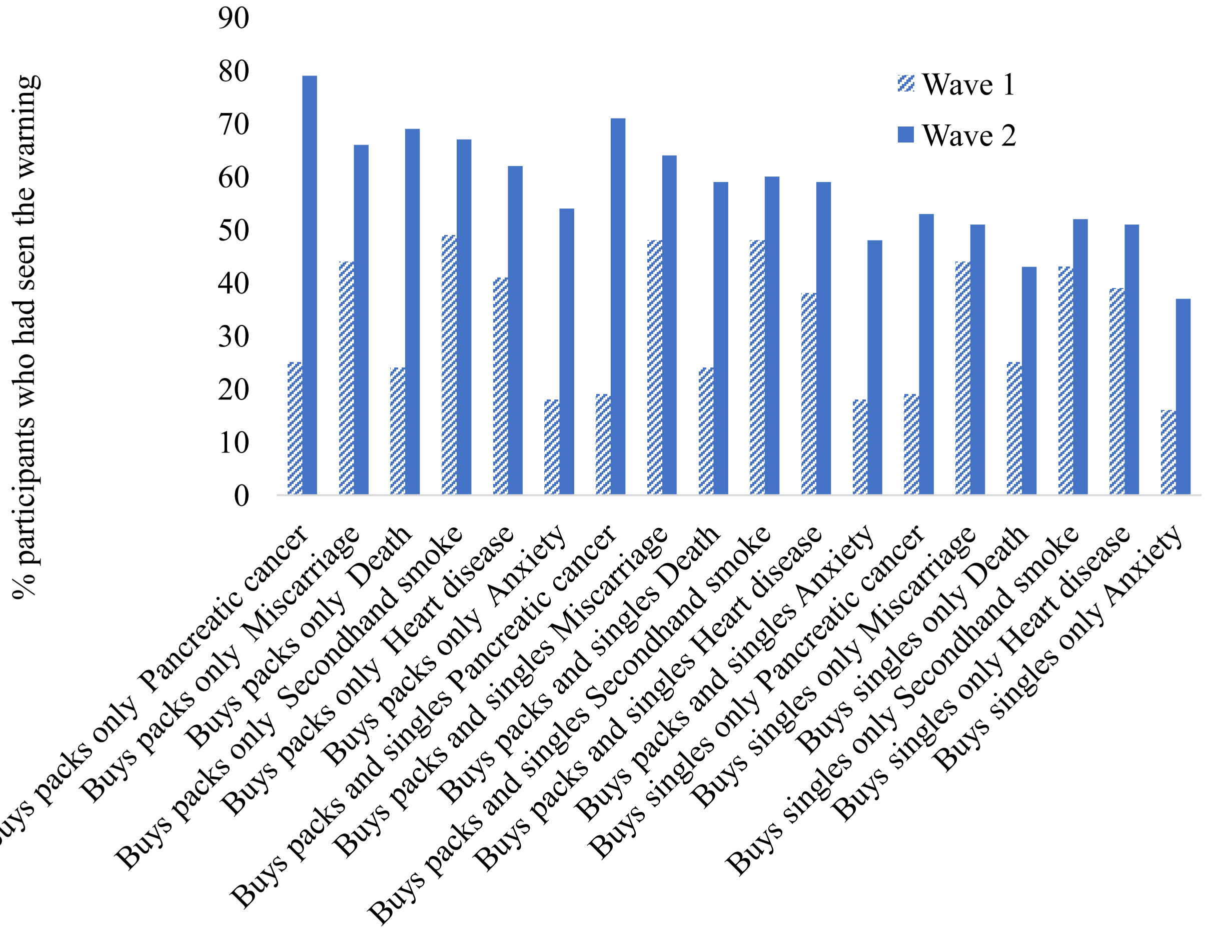

only

Supplementary Material – Figure 3. Percentage of participants who report having seen the new tobacco health warnings by data collection wave and smoking status (i.e., daily smoker, weekly smoker).

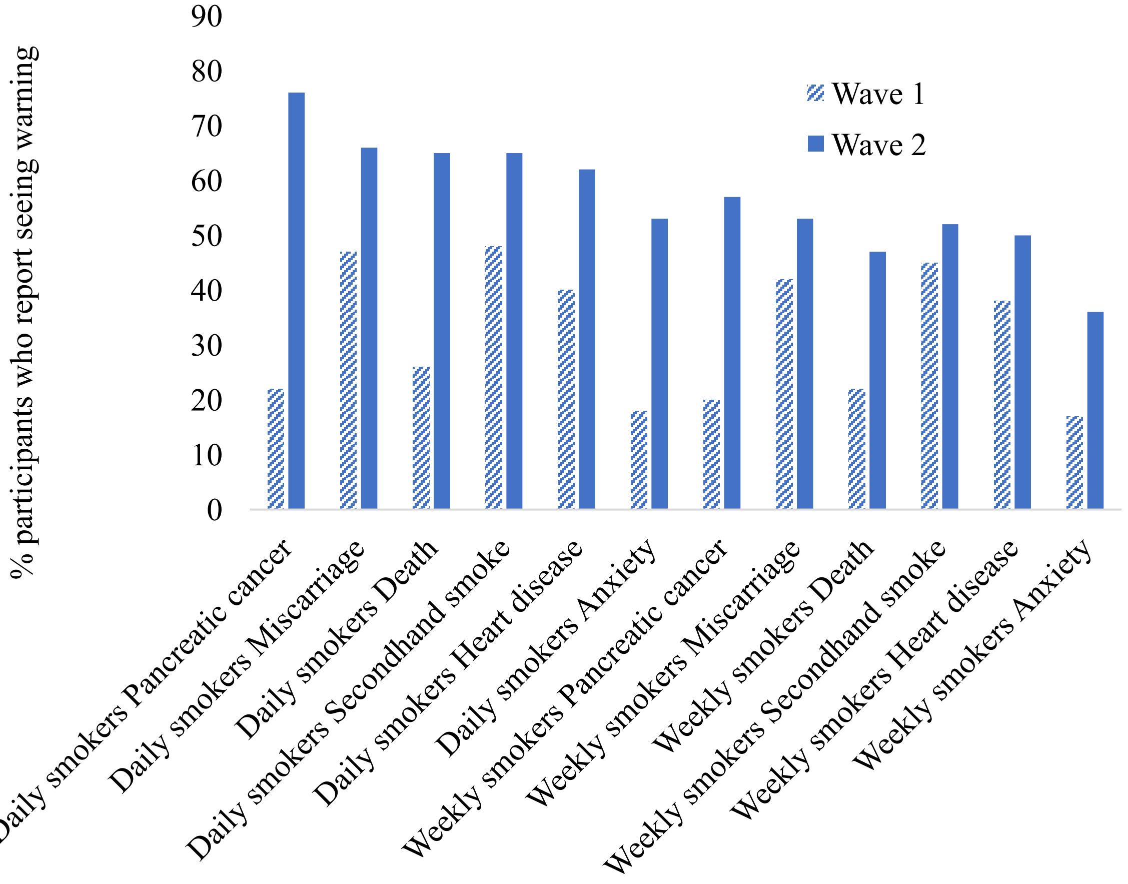

ew only

BMJ Open

A cross-sectional online survey of the impact of new tobacco health warnings in Colombia.

Journal:	*BMJ Open*
Manuscript ID	bmjopen-2021-056754.R1
Article Type:	Original research
Date Submitted by the Author:	15-Feb-2022

Complete List of Authors:	Adams, Sally; University of Bath, Psychology<br>Clavijo, Arturo; Universidad Nacional de Colombia, Psychology<br>Tamayo, Ricardo ; Universidad Nacional de Colombia, Psychology<br>Maynard, Olivia ; Univ Bristol, School of Experimental Psychology

**Primary Subject Heading**:	Public health
Secondary Subject Heading:	Global health
Keywords:	Health policy < HEALTH SERVICES ADMINISTRATION & MANAGEMENT, Substance misuse < PSYCHIATRY, PUBLIC HEALTH

SCHOLARONE™ Manuscripts

I, the Submitting Author has the right to grant and does grant on behalf of all authors of the Work (as defined in the below author licence), an exclusive licence and/or a non-exclusive licence for contributions from authors who are: i) UK Crown employees; ii) where BMJ has agreed a CC-BY licence shall apply, and/or iii) in accordance with the terms applicable for US Federal Government officers or employees acting as part of their official duties; on a worldwide, perpetual, irrevocable, royalty-free basis to BMJ Publishing Group Ltd ("BMJ") its licensees and where the relevant Journal is co-owned by BMJ to the co-owners of the Journal, to publish the Work in this journal and any other BMJ products and to exploit all rights, as set out in our licence.

The Submitting Author accepts and understands that any supply made under these terms is made by BMJ to the Submitting Author unless you are acting as an employee on behalf of your employer or a postgraduate student of an affiliated institution which is paying any applicable article publishing charge ("APC") for Open Access articles. Where the Submitting Author wishes to make the Work available on an Open Access basis (and intends to pay the relevant APC), the terms of reuse of such Open Access shall be governed by a Creative Commons licence – details of these licences and which Creative Commons licence will apply to this Work are set out in our licence referred to above.

Other than as permitted in any relevant BMJ Author's Self Archiving Policies, I confirm this Work has not been accepted for publication elsewhere, is not being considered for publication elsewhere and does not duplicate material already published. I confirm all authors consent to publication of this Work and authorise the granting of this licence.

For peer review only

A cross-sectional online survey of the impact of new tobacco health warnings in Colombia.

Sally Adams[1]; Alvaro Arturo Clavijo-Alvarez[2]; Ricardo M Tamayo[2]; Olivia M Maynard[3,4]

[1] Addiction and Mental Health Group (AIM), Department of Psychology, University of Bath, UK

[2] Departamento de Psicología, Universidad Nacional de Colombia, Colombia

[3] School of Psychological Science, University of Bristol, UK

[4] MRC Integrative Epidemiology Unit (IEU), University of Bristol, Bristol, UK

Corresponding author: Sally Adams, Addiction and Mental Health Group, Department of Psychology, University of Bath, 10 West, Bath BA2 7AY, United Kingdom. T: +44. 1225. 384004; F: +44. 1225. 386752; E:S.Adams@bath.ac.uk

Word count: 3,763

For peer review only

ABSTRACT

Objectives This study aimed to explore the impact of a new set of six pictorial warnings introduced in 2018.

Design and Setting Using a cross-sectional design, we examined awareness of the new warnings among Colombian smokers across two time points of data collection.

Participants Adult smokers (≥ 18 years of age), defined as having smoked at least 100 cigarettes in their lifetime and currently smoking at least one cigarette per week participated at Time 1, prior to the introduction of the new health warnings in Colombia in 2018 (n=1985, 72% male), and at Time 2, twelve-months post-introduction (n=1572, 69% male).

Primary Outcomes At each time we examined smokers' responses to warnings on packs (negative affect, thinking about warning messages, cognitive elaboration), attitudes towards smoking (perceived likelihood and severity of harm, self-efficacy, response-efficacy and quit intentions), knowledge of the health risks of smoking and responses to the new warnings (negative affect, believability, thinking about the harms, reactance and perceived message effectiveness).

Results Awareness of the warnings was low, with only 59% of smokers reporting having seen them at Time 2. Between times, we observed a reduction in negative affect towards current warnings ($p < 0.001$), reduced thinking about ($p < 0.001$) and cognitive elaboration of the warning message ($p < 0.001$), and an increase in perceived severity of warnings ($p < 0.001$). When asked about the six new health warnings, we found a reduction in negative affect (ps < 0.07), cognitions related to harm (ps < 0.01), believability (ps < 0.03), reactance (ps < 0.01) and perceived message effectiveness (ps < 0.02) between times.

Conclusions Our data indicate that effectiveness was low prior to the introduction of the new health warnings and at twelve-months post-introduction. Tobacco control policy should seek to improve exposure to tobacco health warnings in Colombia and to improve their effectiveness.

For peer review only

Keywords: tobacco health warnings; tobacco control; policy; cross-sectional survey

For peer review only

STRENGTHS AND LIMITATIONS

- Our study includes a large sample size, representing smokers from over 100 different areas in Colombia.
- Our work represents some of the first empirical data examining the impact of tobacco control measures such as warnings in Colombia.
- Our study is limited by the use of self-reported data on the perceived effectiveness of health warnings, where it is difficult to determine whether these responses accurately reflect behaviour
- We were unable to make a direct comparison between participants' responses between study times, as different samples of smokers were recruited. We also observed that many participants failed our attention check, highlighting the limitation of using online self-report data.

For peer review only

INTRODUCTION

Every year, over 20,900 Colombian adults die from tobacco-related diseases and despite this, 10% of the population continue to use tobacco each day.[1] Colombia is an emerging market economy, where tobacco use is a growing public health challenge.[2] In response, Colombia has introduced tobacco control strategies including restrictions on labelling and packaging of tobacco products, smoke-free places, restrictions to advertising and sales.[3]

Warnings on tobacco products are the most cost-effective tool for educating smokers and non-smokers about the health risks of tobacco use.[4] Pictorial warnings (PWs) are more likely to be noticed than text-only warnings,[5-7] are more effective at educating smokers about the health risk of smoking, increasing cognitions about health risks,[5,8] and at increasing motivation to quit smoking,[5,7,9], whilst being less vulnerable to the effects of "wear-out".[8,10] Evidence from low- and middle-income countries indicates that PWs may be more effective in these countries, being one of the few sources of information about tobacco's risks.[4,11,12] Despite this evidence there is high variability in the implementation of warnings in low- and middle-income countries.[13]

PWs were first introduced in Colombia in 2009 and since July 2010 all tobacco products have been required by law to display PWs that cover 30% of the package and are rotated annually. These requirements fall short of the World Health Organisation Framework Convention on Tobacco Control (FCTC) recommendations that warnings cover 50% of the package and that branding is replaced by plain packaging [4]. However, the Colombian Ministry of Health has called for "local evidence" on the effectiveness of larger warnings and plain packaging, before the implementation of these tobacco control measures. In Colombia, smokers' exposure to warnings may be limited. First, implementation is likely to be slow, particularly in rural areas. Each year when new warnings are introduced, retailers have 12 months to stop selling tobacco products with older warnings (referred to here as the 'phase-in period'). Second, there is a high proportion of Colombian smokers who purchase single sticks, rather than packs, who are therefore not exposed to any warnings. Given this uncertainty over the extent to

For peer review only

which Colombian smokers are exposed to new warnings, this one-year cross-sectional study explores smokers' responses to a new set of six pictorial warnings covering 30% of the pack which were introduced in Colombia in 2018.

First, we examined the rate of market saturation and the noticeability of the new warnings among smokers over the implementation period. Given that there is no requirement for retailers to stop selling cigarette packs with the old warnings during the 12-month phase-in period and that many Colombian smokers do not purchase cigarette packs (instead buying single cigarettes), we hypothesised that even a year after implementation, awareness of warnings (a proxy marker of market saturation) will be low (less than 75% of respondents will have seen the warnings). Second, we examined smokers' responses to warnings on packs (negative affect, thinking about warning measures, cognitive elaboration), attitudes towards smoking (perceived likelihood and severity of harm, self-efficacy, response-efficacy and quit intentions), knowledge of the health risks of smoking and responses to the new warnings (negative affect, believability, thinking about the harms, reactance and perceived message effectiveness) across the survey times (baseline and 12-months post introduction). Given relatively small changes between the new and old warnings and a low rate of market saturation, we hypothesised that over our assessment period there would be either a small, or no increase in negative affect, cognitions related to smoking, knowledge regarding health risks of smoking, perceived severity and likelihood of harm from smoking and quit intentions. Third, we examined smokers' responses to each of the new warnings in terms of negative affect, believability, cognitions related to harm, avoidance, reactance and perceived message effectiveness. Due to wear-out (as a result of repeated, albeit potentially limited exposure) of the warnings, we hypothesised that each of these outcomes would be greatest at baseline as compared with 12-months post exposure. Our hypotheses, methods and analysis plan were pre-registered in our study protocol (osf.io/qbr5v).

METHODS

For peer review only

Design

A cross-sectional online survey of Colombian smokers with two times of data collection: one prior to the introduction of the new health warnings, and one 12-months post-introduction. The study was approved by the Universidad Nacional de Colombia, Faculty of Human Sciences Ethics Committee (reference: B.VDIECH-024-18).

Participants

We recruited adult smokers (≥ 18 years of age), defined as having smoked at least 100 cigarettes in their lifetime and currently smoking at least one cigarette per week, from the staff and students at the Universidad Nacional de Colombia and from the general public. The research team used existing mailing lists to contact members of the university across eight cities in Colombia and attended student classes to encourage students to complete the surveys. We recruited participants from the general population through social media, word of mouth and posters in public spaces.

Participants provided their email addresses at baseline. For the second time participants who completed the baseline time were re-contacted to ask if they would like to participate again. We also sent the survey to individuals who may not have participated in the previous times using the methods described above. At each survey time, participants provided their email address and could choose to enter a prize draw to win one of three iPads.

Measures and Materials

The study team translated all questionnaire measures into Spanish. A professional translator also completed a process of 'back-translation'. Following this, we discussed discrepancies and optimal wording and the original Spanish translations were changed. All wording and questions were checked by native Spanish speakers in pre-study piloting.

Health warnings

For peer review only

The six warnings introduced on 21st July 2018 depicted six different health-related consequences of tobacco smoking (miscarriage, pancreatic cancer, heart disease, death, anxiety and secondhand smoke), (see Supplementary Materials - Figure 1).

Smoking behaviour

Participants completed smoking behaviour questions to determine eligibility. Demographic measures also included questions on smoker type and smoking heaviness and the first item of the Fagerström Test for Nicotine Dependence (FTND) [14] (see Supplementary Materials – Table 1).

Awareness of new warnings

Awareness of the new pictorial warnings among Colombian smokers was assessed across two times of data collection.

Responses to warnings seen on tobacco packs

Responses to warnings seen on packs were assessed using measures of negative affect, thinking about warning message and cognitive elaboration.

Attitudes to smoking

Attitudes to smoking were assessed using measures of perceived likelihood of harm, perceived severity of harm from smoking, self-efficacy, response efficacy and quit intentions.

Responses to new warnings

In response to viewing the six new health warnings, participants were asked to complete measures of negative affect, believability, thinking about harms, reactance, perceived message effectiveness.

For peer review only

Knowledge of health risks

We assessed knowledge of the six specific health risks presented on the new health warnings.

Procedure

The first time of data collection occurred between 21st May and 12th July 2018, prior to 21st July 2018, the date at which new warnings could start appearing on the market. The second time of data collection took place between 5th June and 1st August 2019. All procedures were identical at the two times.

Participants accessed the study via Qualtrics and were first shown an information statement before completing a tick-box consent page. All text and questions were presented in Spanish. Participants completed screening questions and those who were ineligible were taken to the end of the study. Eligible participants completed demographic questions, responses to warnings on packs, knowledge of health risks and attitudes towards smoking. An attention check item was included in this section (Please select the strongly agree option to let us know that you read all of the survey instructions). Each of the six new warnings were presented individually on screen in a randomised order, and participants completed questions. After reading a debriefing page, participants who wished to be entered into the prize draw were directed to a second survey. Participants who participated in the first time were re-contacted at the second time to ask if they would like to participate again.

Data analysis

To examine market spread of new warnings over the implementation period we calculated percentages of participants in the two times who reported being aware of seeing each of the six new warnings. In exploratory analyses we examined how warning awareness differs between smoking status (daily, weekly smokers) and smoker type (buys cigarette packs, buys singles, buys both).

To examine the impact of the new warnings, we compared responses to warnings on packs, knowledge of health risks and attitudes to smoking between Time 1 and Time 2 using independent

For peer review only

samples t-tests. We ran these analyses both including and excluding participants who were defined as unexposed to the new warnings at Time 2. We defined participants as 'unexposed' if they reported not seeing (or being unsure whether they had seen) three or more of the six new warnings at Time 2.

To examine smokers' responses to each of the new warnings, we compared negative affect, believability, thinking about the harms, reactance and perceived message effectiveness for each of the six warnings at Time 1 and Time 2 using independent samples t-tests.

The internal consistency of the latent constructs (e.g. negative affect, cognitive elaboration) were assessed using Cronbach's alpha. All latent constructs achieved acceptable to good internal consistency and latent variables were calculated by taking a mean of the individual items (see Supplementary Materials - Table 2).

We conducted analyses using IBM SPSS Statistics 22. The data that form the basis of the results presented here are available from the University of Bath Research Data Repository (TBC).

RESULTS

Data screening and participant characteristics

Data were examined prior to analysis and participants who completed the survey after the closing date (*n*=6 in Time 1) or failed the attention check (*n*=1866 in Time 1 and *n*=988 in Time 2) were removed from further analyses. Participant characteristics are presented by data collection time in Table 1.

[Insert Table 1 about here]

Awareness of new warnings

As expected, participants' awareness of new tobacco health warnings (i.e., participants reporting that they had seen the warning) increased from Time 1 (33%) to Time 2 (59%; see Figure 1). Note that the new warnings had not been introduced on packs by Time 1, so the 33% who reported seeing them were

For peer review only

mistaken. Participants mistakenly reporting that they had seen the new warnings at Time 1 was highest for the secondhand smoke (47%), miscarriage (45%) and heart disease (46%) warnings and lowest for the death (25%), pancreatic cancer (21%) and anxiety (18%) warnings.

[Insert Figure 1 about here]

Awareness of the new health warnings at Time 2 was higher for smokers who reported buying only cigarette packs or both pack and single cigarettes, compared with those who bought only single cigarettes. Awareness of the new health warnings at Time 2 was also higher for daily smokers compared with weekly smokers (see Supplementary Materials Figures 2 and 3).

Responses to warnings seen on tobacco packs

As shown in Table 2, there was evidence of a decrease in negative affect, thinking about warning messages and cognitive elaboration between Time 1 and 2. These findings did not differ when including only participants defined as 'exposed' at Time 2.

Attitudes to smoking

There was strong evidence of an increase in perceived severity between Time 1 and 2 (Table 2), although this difference was not evident after including only participants defined as exposed to the warnings at Time 2. The analysis including only participants exposed at Time 2 also indicated strong evidence of a decrease in self-efficacy and quit intention between times. There was no evidence for any other differences.

Knowledge of health risks

There was strong evidence of an increase in mean knowledge of health risks between times across all

For peer review only

warnings, when including only participants defined as exposed at Time 2 (see Table 2). For individual warnings, there was evidence of a small, but statistically meaningful increase in knowledge for the health risks “Smoking causes pancreatic cancer” and “Smoking causes anxiety” between times. The analysis including only participants exposed to the warnings at Time 2 indicated that there was also evidence of an increase between times in knowledge for the health risk “Smoking while pregnant could cause you to lose your baby”. These data were highly skewed, however, with high rates of participants giving scores of either 0 (strongly disagree) or 100 (strongly agree) for each knowledge question (See Table 3).

[Insert Tables 2 and 3 about here]

For peer review only

Responses to new warnings

Differences in responses to the six new health warnings are shown in Table 4.

For the health warning "Miscarriage" there was strong evidence of a decrease in negative affect, cognitions related to harm, believability and perceived message effectiveness between times. For the health warning "Pancreatic cancer" there was strong evidence of a decrease in cognitions related to harm and reactance and an increase in believability between times. For the health warning "Anxiety" there was no evidence of any differences in response to the warning. For the health warning "Heart disease" there was strong evidence of a decrease in negative affect, cognitions related to harm and perceived message effectiveness between wavs. For the health warning "Second-hand smoke" there was strong evidence of a decrease in negative affect, cognitions related to harm, believability and perceived message effectiveness between times. Finally, for the health warning "Death" there was strong evidence of a decrease in negative affect, cognitions related to harm and perceived message effectiveness between times. There was no evidence for any other differences.

[Insert Table 4 about here]

DISCUSSION

Every year since 2010, Colombian smokers have been faced with new health warnings covering 30% of their cigarette packs. Using a cross-sectional design, we examined how smokers responded to the new set of warnings introduced in 2018 and find that they had relatively limited impacts on knowledge of risk and attitudes towards smoking. We do however find some interesting differences between the warnings themselves, and together these findings have important implications for health warning design and implementation in Colombia and worldwide.

For peer review only

As hypothesised, we found that awareness of the warnings was low. Across the six warnings, the average percentage of participants reporting having seen them at Time 2 was 59%. This is particularly low, given that even at Time 1, 33% of participants incorrectly reported having seen the warnings. Given this low level of awareness, it is unsurprising that we see either no changes, or relatively small changes in our measures of responses to warnings on packs, overall knowledge of health risks and attitudes to smoking between Times 1 and 2 – these are outcomes we would expect to improve, had the new warnings made an impact on smokers. Although the differences were small, contrary to our hypothesis, participants reported *lower* negative affect, thinking about the warnings and cognitive elaboration of the warnings on their cigarette packs at Time 2. Even when we only include those participants we defined as 'exposed' to the new warnings at Time 2, these unexpected effects persisted. We saw little to no change in attitudes to smoking between times, although there was some evidence that participants at Time 2 thought that the severity of smoking harms were greater, and they had greater knowledge of the health risks of smoking. However, again, contrary to expectations, when we only examined these effects among participants who we defined as exposed to the new warnings at Time 2, self-efficacy and quit intentions were lower at Time 2.

Together, these findings suggest that the new warnings had relatively little impact on smokers. We consider there to be a number of complementary explanations for this. First, although Time 2 was conducted 12 months after the new warnings were permitted for sale, it is likely that they did not become commonplace for some time after that, as there is no legal requirement for retailers to stop selling packs with older tobacco warnings until 12 months after the implementation period. It is possible that this slow phase-in diminished warnings' impact and meant that even a year after they (in theory) started being available on packs for sale, few participants recalled having seen them. Second, low awareness of warnings is also

For peer review only

likely related to the extent to which smokers engage with cigarette packs. Despite being prohibited by law, many Colombian smokers purchase single cigarettes, [15] and therefore may not be exposed to health warnings at all. Indeed, we find that awareness of the new warnings is greatest for those who smoke daily and those who buy packs.

We do however find some interesting differences between the warnings themselves. As might be expected, we see the highest levels of participants incorrectly reporting at Time 1 that they had seen the new warnings for those with themes used in previous years ('miscarriage', 'heart disease', 'secondhand smoke' were used most recently in 2017 and 'death' was used in 2015; see Supplementary Materials -Table 3). For these four 'familiar' warnings (similar theme, but different wording and pictorial), we see high levels of believability and knowledge of these health risks at both times and only a small proportion of participants reporting that they 'strongly disagreed' that smoking caused these health risks.

However, for all four of these familiar warnings, we observe reductions between times in almost all specific responses to them (e.g., perceived message effectiveness and negative affect). These data may provide support for previous work which has observed a declining impact of warnings (i.e., "wear out") over time. [7,16-19] In contrast to previous work, we found no evidence that cognitive elaboration of risks (e.g., thinking about smoking health risks and quitting) is more resilient to this effect than other responses towards warnings,[10] or that believability is sustained over time. [20-22]. However, wear out assumes that the warnings were initially effective, but become less effective with time. The six warnings tested here received relatively low scores on all measures at Time 1 and although statistically significant, the observed declining impact of warnings at Time 2 was small and may not reflect a meaningful change. These low scores for the responses to the warnings may suggest that the warnings are not effective at influencing responses such as negative affect, cognitions related to harm, believability, and perceived message effectiveness and it is possible that their

For peer review only

effectiveness may be limited by their size. Currently, warnings in Colombia cover 30% of the pack, with the FCTC recommending that warnings should cover at least 50%. It has previously been suggested that sustained effectiveness of warnings may be improved by the introduction of larger pictorial health warnings. [20,23] Although our study was not designed to assess this, our other research in Colombia [24] suggests that larger warnings reduce positive cigarette pack perceptions and reduce demand for cigarettes and future research should investigate the sustained effects of larger Colombian warnings.

There are two warning themes which were new on Colombian cigarette packs in 2018, 'pancreatic cancer' and 'anxiety'. As expected, for these novel warnings, we saw a much smaller percentage of participants incorrectly reporting that they had seen them at Time 1 than the four familiar warnings. However, interestingly, at Time 2, we saw divergent levels of awareness for these two warnings. The largest percentage of participants reported having seen the pancreatic cancer warning of all warnings at Time 2, but the lowest percentage of all participants reported having seen the anxiety warning. We suggest this may be due to the pictorial used, with the pancreatic cancer warning displaying a close-up of a yellow eye, which is arguably novel and memorable, while the anxiety warning depicted a man pulling at his hair. Previous research has suggested that more "severe" or grotesque warnings increase smokers' intentions to quit and are rated as more believable and effective than less severe warnings. [25,26]

Knowledge of the risk of smoking for both pancreatic cancer and anxiety was low at Time 1 and at this time-point we saw the highest percentages of participants reporting that they 'strongly disagreed' that smoking caused these conditions. The inclusion of these new warning messages reflects the priority for the Colombian Ministry of Health to inform the public about "lesser known" smoking health harms. We saw increased knowledge of these two health risks at Time 2, and fewer participants selecting 'strongly disagree' particularly

For peer review only

for anxiety, although knowledge was still lower than for most other health risks. Despite this, these increases suggest that novel warnings can impact smokers' knowledge of smoking risks. We also saw that the anxiety warning had the lowest response scores at both times compared with all other warnings (particularly negative affect, believability, perceived message effectiveness and cognitions related to harm). We suggest that these low scores, combined with low 'knowledge' scores, are in line with the inconsistent cross-sectional evidence regarding the relationship between smoking and anxiety,[27] and evidence from Mendelian randomisation studies which find no causal relationship between smoking and the onset of anxiety.[28] We suggest that it is important to consider the veracity of the health risk claims depicted in health warnings and we raise the important question of whether including health risks which are not supported by the evidence might reduce the overall impact of warnings.

Warnings have repeatedly been shown to be a cost-effective tool for communicating the health risks of smoking to smokers' and non-smokers.[4] For low- and middle-income countries, warnings are proposed to be even more effective.[4] However, for this to occur, smokers must be exposed to warnings and increased noticeability may prevent wear-out effects.[20] Our data suggest that strategies for increasing exposure to warnings are required in Colombia, particularly as a large proportion of smokers purchase single cigarettes rather than packs. One method for achieving this is to include health warning messages on individual cigarette sticks and emerging evidence indicates that this represents a novel and effective method for reducing tobacco use.[29-31]

Our study has numerous strengths, including a large sample size, representing smokers from over 100 different areas in Colombia. Additionally, our work represents some of the first empirical data examining the impact of tobacco control measures such as warnings in Colombia. However, our findings should also be considered in light of the following

For peer review only

limitations. Our study is limited by the use of self-reported data on the perceived effectiveness of health warnings, where it is difficult to determine whether these responses accurately reflect behaviour. We were unable to make a direct comparison between participants' responses between study times, as different samples of smokers were recruited. We also observed that many participants failed our attention check, highlighting the limitation of using online self-report data. Our participants may not be representative of the general population in Colombia and in particular smokers. Internet penetration is estimated at 65% in Colombia [32], where individuals without internet access would not have been able to participate in our survey. Further, our sample of smokers were on average younger, more educated and more likely to be male compared with Colombian smokers reported in previous research [33]. Therefore, our sample may limit the generalisability of our findings to all smokers in Colombia, where further research on the effectiveness of tobacco health warnings for all Colombian smokers is warranted.

CONCLUSIONS

Future tobacco control policy should seek to improve the noticeability of and exposure to tobacco health warnings in Colombia and to reduce the impact of wear-out effects. Updating and rotating tobacco warnings is a major challenge facing regulators in maintaining the effectiveness of health messages.[34] Rotation should include considering what similar warning themes have been used in previous years as well as ensuring that new warnings are well supported by scientific evidence. It is also likely that larger health warnings may prevent wear-out effects. Tobacco control policy may also seek to implement a requirement of retailers to stop selling cigarette packs with older warnings to reduce the phase-in period for new warnings and improve the availability and impact of new warnings.

For peer review only

CONTRIBUTORSHIP

Conception and design of the work: SA, RMT, AACA, OMM. Analysis: SA and OMM. Interpretation of data for the work: all authors. Drafting the work: SA and OMM. Revising it critically: all authors. Final approval of the version to be published: all authors.

FUNDING

This research was funded by an ESRC New Investigator's Award, awarded to Dr Olivia Maynard (ES/R003424/1) and a University of Bristol Global Challenges Research Ad Hoc Fund.

COMPETING INTERESTS

RMTO and AACA were previously hired by the Colombian Ministry of Health to design the six warnings used here. The Ministry of Health had no role in the design of the study.

PATIENT AND PUBLIC INVOLVEMENT

It was not appropriate or possible to involve patients or the public in the design, or conduct, or reporting, or dissemination plans of our research

DATA AVAILABILITY

The data will be made available for sharing via a University of Bristol online data repository

For peer review only

TABLES

Table 1. Participant characteristics by data collection time

	***n* = 1985**	***n* = 1572**	***p* -value [CIs]**
Sex (% male)	72	69	N/A
Age (years)	26 (9)	26 (8)	0.03 [-1.098 - 0.049]
Location (% Bogota)	49	67	< 0.001
Smoking frequency			
Daily smokers (%)	65	62	0.06
Cigarettes per day (daily smokers)	7 (6)	7 (5)	N/A
Weekly smokers (%)	35	38	0.06
Cigarettes per week (weekly smokers)	10 (10)	9 (9)	0.09
How many days a week do you smoke? (weekly smokers)	3 (1)	3 (1)	0.42
Cigarettes per day (weekly smokers)	3 (3)	3 (3)	0.38
Type of packs bought			
Buys cigarette packs (%)	38	31	< 0.001
Buys single cigarettes (%)	22	26	N/A
Buys both packs and single cigarettes (%)	39	41	0.21
Dependence			
Smokes within first 60 minutes of waking (%)	27	24	0.02

For peer review only

Note. Values are %, except for age, cigarettes per day, cigarettes per week, smoking days per week -mean (standard deviation). P-values represent differences in variables between times (t-tests/chi-square).

Table 2. Responses to tobacco health warnings and attitudes to smoking by data collection time (for all participants and excluding participants unexposed to new warnings at Time 2).

		**Time 1 vs Time 2 - including all participants at Time 2**			**Time 1 vs Time 2 - only including participants exposed to new warnings at Time 2**				
	**Time 1**	**Time 2**	***t***	***df*** [b]	***p* - value**	**Time 2**	***t***	***df*** [b]	***p* - value**
	(*n* = 1985)	(*n* = 1572)				(n = 910)			
Responses to warnings on packs									
Negative affect	41(27)	38 (26)	3.03	3411	<0.001	38 (26)	3.35	2893	<0.001
Thinking about warning messages	31 (26)	28 (25)	3.28	3454	<0.001	27 (25)	3.28	1880	<0.001
Cognitive elaboration	46 (31)	42 (29)	3.54	3432	< 0.001	42 (29)	3.59	1867	< 0.001
Knowledge of health risks (overall)	73 (17)	74 (16)	-1.78	3443	0.08	76 (17)	-3.91	2893	< 0.001
Lose your baby[a]	85 (22)	85 (21)	-0.59	3470	0.56	87 (20)	-2.24	1979	0.03
Smoking causes pancreatic cancer	68 (29)	70 (27)	-2.2	3555	0.03	72 (28)	-3.53	2893	< 0.001
Secondhand smoke is deadly	74 (27)	73 (26)	1.2	3555	0.23	74 (26)	0.41	2893	0.68
Smoking damages your heart	83 (21)	83 (20)	-0.03	3555	0.98	84 (20)	-0.9	2893	0.37
Smoking causes anxiety	60 (36)	64 (34)	-3.41	3471	<0.001	67 (33)	-5.67	1948	< 0.001
Smoking causes a slow and painful death	68 (30)	68 (28)	-0.59	3460	0.56	70 (28)	-1.93	1873	0.05
Attitudes to smoking									
Perceived likelihood of harm	78 (19)	77 (17)	0.61	3467	0.54	78 (17)	-0.3	1887	0.76
Perceived severity of smoking harms	87 (17)	89 (15)	-3.97	3501	< 0.001	88 (16)	-1.73	1815	0.08
Self-efficacy	69 (32)	67 (32)	1.65	3555	0.1	64 (33)	3.73	1708	< 0.001
Response-efficacy	83 (24)	84 (22)	-0.54	3487	0.59	84 (22)	-0.32	1899	0.75
Quit intention	60 (30)	59 (30)	1.61	3554	0.11	56 (31)	3.9	2892	< 0.001

For peer review only

Note. Vales are mean (standard deviation), *t*-score, degrees of freedom and *p*-values

[a] Full wording is 'Smoking while pregnant could cause you to lose your baby'

[b] Degrees of freedom differ according to equality of variances of variables

For peer review only

Table 3. Knowledge of health risks (percentage of participants giving a score of 0 or 100) by data collection time (for all participants and excluding participants unexposed to new warnings at Time 2).

			**Time 1 vs Time 2 including all participants at Time 2**		**Time 1 vs Time 2 - only including participants exposed to new warnings at Time 2**	
	**Time 1**		**Time 2**		**Time 2**	
	(*n* = 1985)		(*n* = 1572)		(n = 910)	
	% giving a score of 0	% giving a score of 100	% giving a score of 0	% giving a score of 100	% giving a score of 0	% giving a score of 100
**Warning 1 "Pancreatic cancer"**	4	24	3	25	3	28
**Warning 2 "Miscarriage"**	1	50	1	48	1	51
**Warning 3 "Death"**	5	25	3	23	3	25
**Warning 4 "Secondhand smoke"**	2	31	1	27	1	30
**Warning 5 "Heart disease"**	1	41	1	39	1	42
**Warning 6 "Anxiety"**	11	25	7	25	6	28

Note. Values are % of participants giving a score of 0 ‘strongly disagree’ or 100 “strongly agree” for the six new warnings

For peer review only

Table 4. Responses to the new tobacco health warnings by data collection time

	Time 1	Time 2	*t*	*df*[a]	*p*-value
	(*n* = 1985)	(*n* = 1572)			
**Warning 1 "Pancreatic cancer"**					
Negative affect	47 (30)	45 (28)	1.79	3093	0.07
Cognitions related to harm	59 (32)	57 (32)	2.55	3134	0.01
Believability	57 (32)	60 (31)	-2.97	3076	< 0.001
Reactance	41 (25)	39 (25)	2.52	3134	0.01
Perceived message effectiveness	48 (27)	47 (26)	1.52	3134	0.13
**Warning 2 "Miscarriage"**					
Negative affect	50 (30)	44 (29)	5.57	3128	<0.001
Cognitions related to harm	59 (34)	51 (35)	6.16	2984	<0.001
Believability	67 (31)	64 (31)	2.92	3128	< 0.001
Reactance	43 (27)	42 (27)	0.84	3128	0.4
Perceived message effectiveness	51 (27)	47 (27)	4.1	3128	<0.001
**Warning 3 "Death"**					
Negative affect	55 (30)	51 (29)	3.52	3136	<0.001
Cognitions related to harm	67 (31)	64 (31)	2.75	3136	0.01
Believability	68 (30)	66 (30)	1.5	3136	0.13
Reactance	42 (27)	41 (25)	0.4	3096	0.69
Perceived message effectiveness	54 (26)	52 (27)	2.31	3136	0.02
**Warning 4 "Secondhand smoke"**					
Negative affect	46 (29)	42 (28)	3.52	3123	<0.001
Cognitions related to harm	61 (33)	55 (33)	4.95	3123	<0.001
Believability	69 (30)	66 (30)	2.19	3123	0.03
Reactance	40 (25)	40 (25)	-0.19	3123	0.85
Perceived message effectiveness	48 (26)	45 (26)	3.39	3123	< 0.001
**Warning 5 "Heart disease"**					
Negative affect	49 (30)	45 (28)	3.33	3068	< 0.001
Cognitions related to harm	64 (31)	60 (31)	3.41	3112	< 0.001
Believability	66 (30)	65 (30)	1.6	3112	0.11
Reactance	38 (25)	37 (24)	1.51	3058	0.13

For peer review only

Perceived message effectiveness	49 (26)	46 (25)	3.47	3112	< 0.001
**Warning 6 "Anxiety"**					
Negative affect	30 (28)	29 (26)	1.2	3089	0.23
Cognitions related to harm	42 (35)	41 (33)	1.04	3084	0.3
Believability	51 (34)	53 (34)	-1.4	3131	0.16
Reactance	41 (25)	40 (25)	1.14	3131	0.26
Perceived message effectiveness	36 (25)	35 (25)	0.93	3131	0.35

Note. Values are mean (standard deviation), *t*-score, degrees of freedom and *p*-values

[a] Degrees of freedom differ according to equality of variances of variables

For peer review only

REFERENCES

1 American Cancer Society (2021, April 28). Tobacco Atlas: Colombia https://tobaccoatlas.org/country/colombia/

2 Posada-Villa, J., Cheng, H. G., Martins, S. S., Storr, C. L., Aguilar-Gaxiola, S. A., & Anthony, J. C. (2011). The tobacco dependence dimension in Colombia. *Revista panamericana de salud publica = Pan American journal of public health*, *29*(1), 52–56.

3 Campaign for Tobacco Free Kids (2021, April 28) Legislation by Country: Colombia https://www.tobaccocontrollaws.org/legislation/country/colombia/pl-health-warnings

4 Fong, G. T., Hammond, D., & Hitchman, S. C. (2009). The impact of pictures on the effectiveness of tobacco warnings. *Bulletin of the World Health Organization*, *87*(8), 640–643. https://doi.org/10.2471/blt.09.069575

5 Hammond, D., Fong, G. T., McNeill, A., Borland, R., & Cummings, K. M. (2006). Effectiveness of cigarette warning labels in informing smokers about the risks of smoking: findings from the International Tobacco Control (ITC) Four Country Survey. *Tobacco control*, *15 Suppl 3*(Suppl 3), iii19–iii25. https://doi.org/10.1136/tc.2005.012294

6 Thrasher, J. F., Hammond, D., Fong, G. T., & Arillo-Santillán, E. (2007). Smokers' reactions to cigarette package warnings with graphic imagery and with only text: a comparison between Mexico and Canada. *Salud publica de Mexico*, *49 Suppl 2*, S233–S240. https://doi.org/10.1590/s0036-36342007000800013

For peer review only

7 Borland, R., Wilson, N., Fong, G. T., Hammond, D., Cummings, K. M., Yong, H. H., Hosking, W., Hastings, G., Thrasher, J., & McNeill, A. (2009a). Impact of graphic and text warnings on cigarette packs: findings from four countries over five years. *Tobacco control*, *18*(5), 358–364. https://doi.org/10.1136/tc.2008.028043

8 Li, J., & Grigg, M. (2009). New Zealand: new graphic warnings encourage registrations with the quitline. *Tobacco control*, *18*(1), 72. https://doi.org/10.1136/tc.2008.027649

9 Borland, R., Yong, H. H., Wilson, N., Fong, G. T., Hammond, D., Cummings, K. M., Hosking, W., & McNeill, A. (2009b). How reactions to cigarette packet health warnings influence quitting: findings from the ITC Four-Country survey. *Addiction (Abingdon, England)*, *104*(4), 669–675. https://doi.org/10.1111/j.1360-0443.2009.02508.x

10 Hammond, D., Fong, G. T., Borland, R., Cummings, K. M., McNeill, A., & Driezen, P. (2007). Text and graphic warnings on cigarette packages: findings from the international tobacco control four country study. *American journal of preventive medicine*, *32*(3), 202–209. https://doi.org/10.1016/j.amepre.2006.11.011

11 Swayampakala, K., Thrasher, J. F., Yong, H. H., Nagelhout, G. E., Li, L., Borland, R., Hammond, D., O'Connor, R. J., & Hardin, J. W. (2018). Over-Time Impacts of Pictorial Health Warning Labels and their Differences across Smoker Subgroups: Results from Adult Smokers in Canada and Australia. *Nicotine & tobacco research: official journal of the Society for Research on Nicotine and Tobacco*, *20*(7), 888–896. https://doi.org/10.1093/ntr/ntx134

For peer review only

12 FCTC (2009) article 11: tobacco warning labels: evidence and recommendations from the ITC project Waterloo, ON: International Tobacco Control Policy Evaluation Project. http://www.itcproject.org

13 Wipfli, H. L., Fujimoto, K., & Valente, T. W. (2010). Global tobacco control diffusion: the case of the framework convention on tobacco control. *American journal of public health*, *100*(7), 1260–1266. https://doi.org/10.2105/AJPH.2009.167833

14 Heatherton, T. F., Kozlowski, L. T., Frecker, R. C., & Fagerström, K. O. (1991). The Fagerström Test for Nicotine Dependence: a revision of the Fagerström Tolerance Questionnaire. *British journal of addiction*, *86*(9), 1119–1127. https://doi.org/10.1111/j.1360-0443.1991.tb01879.x

15 Maldonado, N., Llorente, B., Escobar, D., et al (2020). Smoke signals: monitoring illicit cigarettes and smoking behaviour in Colombia to support tobacco taxes. *Tobacco Control,* 29, 243-248.

16 Woelbert, E., d'Hombres, B. (2019). Pictorial health warnings and wear-out effects: evidence from a web experiment in 10 European countries. *Tobacco Control,* 28, 71-76.

17 Environics Research Group (1999) Canadian adult and youth opinions on the sizing of health warning messages. Ottawa, Canada.

18 Environics Research Group (2000) Testing new health warning messages for cigarette packages: A summary of three phases of focus group research: Final report. Ottawa, Canada.

19 Brubaker, R. G., & Mitby, S. K. (1990). Health-risk warning labels on smokeless tobacco products: are they effective?. *Addictive behaviors*, *15*(2), 115–118. https://doi.org/10.1016/0306-4603(90)90014-o

20 Anshari, D., Yong, H., Borland, R., et al (2018). Which type of tobacco product warning imagery is more effective and sustainable over time? A longitudinal assessment of smokers in Canada, Australia and Mexico. *BMJ Open, 8***.** doi: 10.1136/bmjopen-2018-021983

21 Shanahan, P., & Elliott, D. (2008) Evaluation of the Effectiveness of the Graphic Health Warnings on Tobacco Product Packaging. Canberra, Australian Government Department of Health and Aging. Evaluation of effectiveness of graphic health warnings on tobacco product packaging

22 Environics Research Group (2002) The health effects of tobacco and health warning messages on cigarette packages: Survey of adults and adult smokers. Toronto, Canada.

23 Li, L., Borland, R., Yong, H., Cummings, K. M., Thrasher, J. F., Hitchman, S. C., Fong, G. T., Hammond, D., & Bansal-Travers, M. (2015). Longer term impact of cigarette package warnings in Australia compared with the United Kingdom and Canada. *Health education research*, *30*(1), 67–80. https://doi.org/10.1093/her/cyu074

For peer review only

24 Sillero-Rejon, C, Mahmoud, O, Tamayo, RM, Clavijo-Alvarez, AA, Adams, S, Maynard, OM. Standardised packs and larger health warnings: visual attention and perceptions among Colombian smokers and non-smokers. *Addiction*. 2022; 1– 11. https://doi.org/10.1111/add.15779

25 Maynard, O.M., Gove, H., Skinner, A.L. *et al.* (2018) Severity and susceptibility: measuring the perceived effectiveness and believability of tobacco health warnings. *BMC Public Health* 18**,** 468. https://doi.org/10.1186/s12889-018-5385-x

26 Kees, J., Burton, S., Andrews, J. C., & Kozup, J. (2010). Understanding how Graphic Pictorial Warnings Work on Cigarette Packaging. *Journal of Public Policy & Marketing*, *29*(2), 265–276. https://doi.org/10.1509/jppm.29.2.265

27 Fluharty, M., Taylor, A. E., Grabski, M., & Munafò, M. R. (2017). The Association of Cigarette Smoking with Depression and Anxiety: A Systematic Review. *Nicotine & tobacco research: official journal of the Society for Research on Nicotine and Tobacco*, *19*(1), 3–13. https://doi.org/10.1093/ntr/ntw140

28 Taylor, A. E., Fluharty, M. E., Bjørngaard, J. H., Gabrielsen, M. E., Skorpen, F., Marioni, R. E., Campbell, A., Engmann, J., Mirza, S. S., Loukola, A., Laatikainen, T., Partonen, T., Kaakinen, M., Ducci, F., Cavadino, A., Husemoen, L., Ahluwalia, T. S., Jacobsen, R. K., Skaaby, T., Ebstrup, J. F., … Munafò, M. R. (2014). Investigating the possible causal association of smoking with depression and anxiety using Mendelian randomisation meta-analysis: the CARTA consortium. *BMJ open*, *4*(10), e006141. https://doi.org/10.1136/bmjopen-2014-006141

29 Drovandi, A., Teague, P.A., Glass, B., Malau-Aduli, B. (2019) Do health warnings on cigarette sticks dissuade smokers and non-smokers? A focus group and interview study of Australian university students. *Psychol Res Behav Manag*. 12:361-373 https://doi.org/10.2147/PRBM.S193754

30 Moodie, C.S., Hiscock, R,. Thrasher, J. *et al.* (2018) Perceptions of cigarette pack inserts promoting cessation and dissuasive cigarettes among young adult smokers in the UK: a cross-sectional online survey. *BMJ Open* 8:e019662. doi: 10.1136/bmjopen-2017-019662

31 Moodie, C., Gendall, P., Hoek, J., MacKintosh, A. M., Best, C., & Murray, S. (2019). The Response of Young Adult Smokers and Nonsmokers in the United Kingdom to Dissuasive Cigarettes: An Online Survey. *Nicotine & tobacco research : official journal of the Society for Research on Nicotine and Tobacco*, *21*(2), 227–233. https://doi.org/10.1093/ntr/ntx261

32 Statista. (2022, January 19) Percentage of population using the internet in Colombia from 2000 to 2019. https://www.statista.com/statistics/209109/number-of-internet-users-per-100-inhabitants-in-colombia-since-2000/

33 Storr, C. L., Cheng, H., Posada-Villa, J., Aguilar-Gaxiola, S., & Anthony, J. C. (2008). Adult smokers in Colombia: who isn't giving it up?. *Addictive behaviors*, *33*(3), 412–421. https://doi.org/10.1016/j.addbeh.2007.10.003

34 Hammond, D. & Reid, J.L. (2012) Health warnings on tobacco products: International practices. Salud publica de Mexico, *54* (3), 270-80. Doi:10.1590/S0036-36342012000300009

For peer review only

Figure 1. Percentage of participants who report being aware of having seen the new warnings at Wave 1 and 2

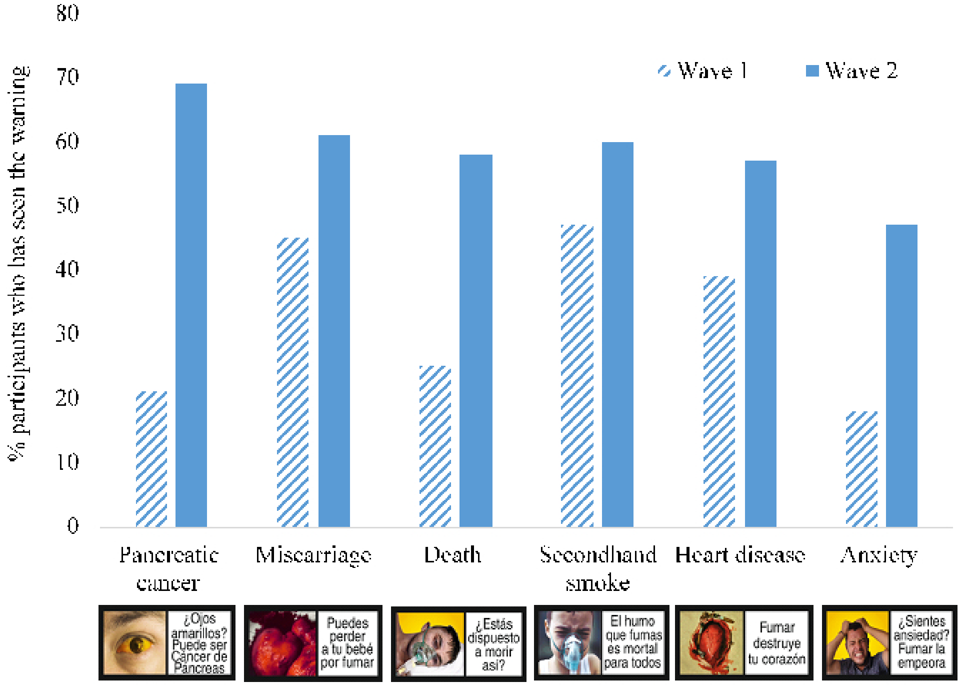

185x153mm (96 x 96 DPI)

Supplementary Materials Table 1. Survey Questions

Variable	Items	Response
Smoking behaviour - eligibility	Have you smoked at least 100 cigarettes in your lifetime?	1 = Yes<br>2 = No
	How often do you smoke cigarettes?	1 = Every day<br>2 = Every week<br>3 = Less than every week<br>4 = Not al all
Smoking heaviness – daily smokers	Approximately how many cigarettes do you smoke per day?	Provide a single number
Smoking heaviness -non-daily smokers	Approximately how many days a week do you smoke	
	On the days that you do smoke, approximately how many cigarettes do you smoke	
	Approximately how many cigarettes do you smoke per week?	
Fagerström Test for Nicotine Dependence (FTND)	How soon after you wake do you smoke your first cigarette?	1 = Within 5 minutes<br>2 = 6 to 30 minutes<br>3 = 31 to 60 minutes<br>4 = After 60 minutes
Awareness of new warnings	Have you seen this health warning on a cigarette pack?	1= Yes<br>2 = No<br>3 = Unsure
Responses to warnings seen on tobacco packs		
Negative affect	In the last week, the warning on my cigarette pack made me feel...	
	…scared	0 = Strongly disagree<br>25 = Somewhat disagree<br>50 = Neither agree nor disagree<br>75 = Somewhat agree<br>100 = Strongly agree
	…regretful	
	…disgusted	

For peer review only

Thinking about the warning message	When I notice my cigarette pack, I think about the message that the warning conveys	0 = Strongly disagree<br>25 = Somewhat disagree<br>50 = Neither agree nor disagree<br>75 = Somewhat agree<br>100 = Strongly agree
	When my cigarette pack is not<br>in sight, I think about the message that the warning conveys	
Attitudes towards smoking		
Perceived likelihood of harm	What is the chance that you will one day get the following diseases if you continue to smoke cigarettes?	
	…heart disease?	0 = No chance<br>25 = Low chance<br>50 = Moderate chance<br>75 = High chance<br>100 = Certain
	…cancer?	
	…a permanent breathing problem?	
Perceived severity of harm from smoking	How much would getting the following diseases because of smoking affect your life?	
	…heart disease?	0 = No chance<br>25 = Low chance<br>50 = Moderate chance<br>75 = High chance<br>100 = Certain
	…cancer?	
	…a permanent breathing problem?	
Self-efficacy	I see myself as being capable of quitting smoking in the next 2 months if I wanted to	0 = Strongly disagree<br>25 = Somewhat disagree<br>50 = Neither agree nor disagree<br>75 = Somewhat agree<br>100 = Strongly agree
Response-efficacy	Quitting smoking would lower my chances of getting a<br>disease related<br>to smoking	

Quit intentions	Are you planning to quit smoking within the next 6 months?	0 = Very unlikely<br>25 = Unlikely<br>50 = Maybe, maybe not<br>75 = Likely<br>100 = Very likely
Responses to new warnings		
Negative affect	This warning makes me feel…	
	…scared	0 = Strongly disagree<br>25 = Somewhat disagree<br>50 = Neither agree nor disagree<br>75 = Somewhat agree<br>100 = Strongly agree
	…regretful	
	…disgusted	
Believability	This warning is believable	0 = Strongly disagree<br>25 = Somewhat disagree<br>50 = Neither agree nor disagree<br>75 = Somewhat agree<br>100 = Strongly agree
Thinking about harms	This warning makes me think about the health problems caused by smoking	0 = Strongly disagree<br>25 = Somewhat disagree<br>50 = Neither agree nor disagree<br>75 = Somewhat agree<br>100 = Strongly agree
Reactance	This warning is trying to manipulate me	0 = Strongly disagree<br>25 = Somewhat disagree<br>50 = Neither agree nor disagree<br>75 = Somewhat agree<br>100 = Strongly agree
	The health effect of this warning is overblown	
	This warning annoys me	
Perceived message effectiveness	This warning discourages me from wanting to smoke	0 = Strongly disagree<br>25 = Somewhat disagree<br>50 = Neither agree nor disagree<br>75 = Somewhat agree<br>100 = Strongly agree
	This warning makes smoking seem unpleasant to me	
	This warning makes me concerned about the health effects of smoking	
	If I saw this warning on my	

For peer review only

	cigarette pack, I would...	
	...try to avoid thinking about it	
	…try to avoid looking at it	
	…keep the pack out of sight to avoid looking at the warning	
Knowledge of health risks	Say how much you agree or disagree with each statement below:	0 = Strongly disagree<br>25 = Somewhat disagree<br>50 = Neither agree nor disagree<br>75 = Somewhat agree<br>100 = Strongly agree
	Smoking while pregnant could cause you to lose your baby	
	Smoking causes pancreatic cancer	
	Secondhand smoke is deadly	
	Smoking damages your heart	
	Smoking causes anxiety	
	Smoking causes a slow and painful death	

For peer review only

Supplementary Material – Table 2. Internal consistency of latent constructs

**Latent construct**	**Number of items**	**α**	***r***
Responses to warnings on packs			
Negative affect	3 items	0.79	0.49 – 0.69
Thinking about warning message	2 items	0.66	0.5
Cognitive elaboration	2 items	0.8	0.66
Attitudes to smoking			
Perceived likelihood of harm	3 items	0.84	0.61-0.65
Perceived severity	3 items	0.87	0.66-0.71
Responses to new warnings			
**Warning 1 "Pancreatic cancer"**			
Negative affect	3 items	0.83	0.54 – 0.77
Reactance	3 items	0.68	0.39 – 0.45
Perceived effectiveness	6 items	0.87	0.34 – 0.82
**Warning 2 "Miscarriage"**			
Negative affect	3 items	0.77	0.38 – 0.79
Reactance	3 items	0.69	0.41 – 0.44
Perceived effectiveness	6 items	0.86	0.28 – 0.75
**Warning 3 "Death"**			
Negative affect	3 items	0.8	0.46 – 0.77
Reactance	3 items	0.69	0.43 – 0.44
Perceived effectiveness	6 items	0.86	0.33 – 0.82
**Warning 4 "Secondhand Smoke"**			
Negative affect	3 items	0.81	0.49 – 0.67
Reactance	3 items	0.67	0.37 – 0.44
Perceived effectiveness	6 items	0.86	0.31 – 0.80
**Warning 5 "Heart disease"**			
Negative affect	3 items	0.83	0.53 – 0.80
Reactance	3 items	0.68	0.40 – 0.44
Perceived effectiveness	6 items	0.86	0.33 – 0.80
**Warning 6 "Anxiety"**			
Negative affect	3 items	0.87	0.63 – 0.76
Reactance	3 items	0.64	0.33 – 0.43
Perceived effectiveness	6 items	0.87	0.34 – 0.85

For peer review only

Note. The internal consistency of latent constructs assessed using Cronbach's alpha and associations between the constructs using bivariate correlations.

For peer review only

Supplementary Materials Table 3. – Presence of warning theme between 2010 and 2018.

**Warning theme**	**2010**	**2011**	**2012**	**2013**	**2014**	**2015**	**2016**	**2017**	**2018**
Death							X		X
Secondhand smoke		X			X			X	X
Anxiety									X
Miscarriage		X		X			X	X	X
Heart attack / heart disease					X		X	X	X
Pancreatic cancer									X
Mouth cancer and bad breath	X								
Kidney cancer				X				X	
Breast cancer				X					
Leukemia					X				
Bladder cancer					X				
Laryngeal cancer							X		
Cancer							X		
Damaged arteries					X			X	
Thrombosis					X				
Vascular problems / disease				X					
Cerebral stroke		X							
Lung disease		X					X		
Eye problems / disease		X							
Bone damage				X					
Sexual impotence	X	X		X					
Aging	X								

Data are taken from https://www.tobaccocontrollaws.org/legislation/country/colombia/laws. Data not available for 2012 or 2015.

All warnings are available from the Colombian Ministry of Health and Social Protection.

Colombia. Ministerio de Salud y Protección Social. (2020). Advertencias sanitarias productos de tabaco y derivados 2010 - 2021. [Piezas publicitarias;] Retrieved From: https://www.minsalud.gov.co/sites/rid/Lists/BibliotecaDigital/RIDE/VS/PP/ENT/advertencias-sanitarias-todas-vigencias.zip

For peer review only

Supplementary Material - Figure 1. Six new tobacco health warnings introduced in Colombia July 2018

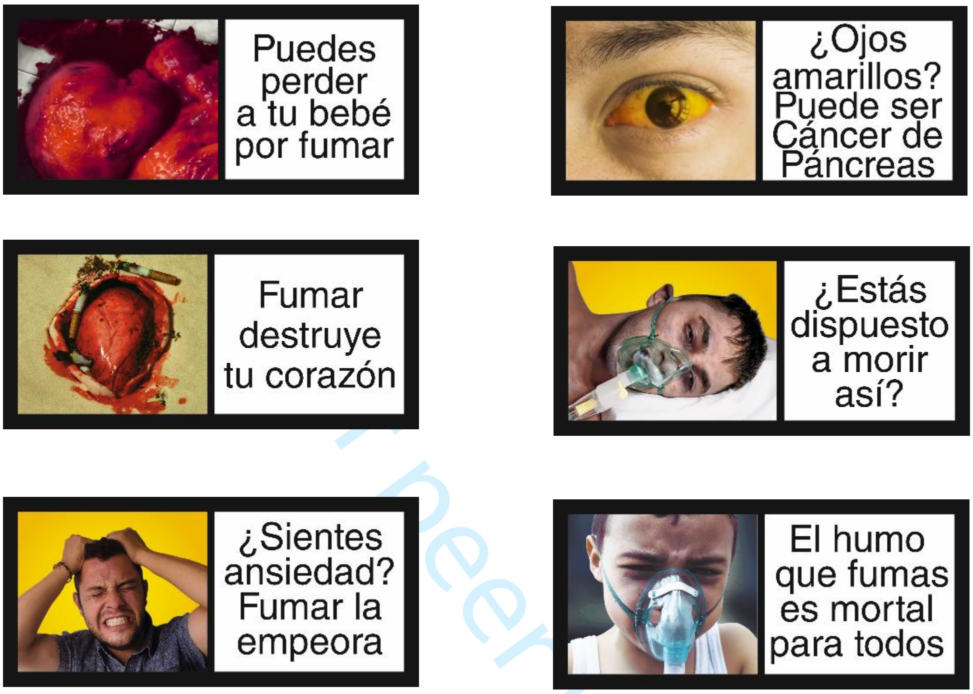

Health warning topics – clockwise *"You can lose your baby by smoking", "Yellow eyes? It could be pancreatic cancer", "Smoking destroys your heart", "Are you willing to die like this?", "Do you feel anxiety? Smoking makes it worse"* and *"The smoke you smoke is deadly for everyone".*

For peer review only

Supplementary Material – Figure 2. Percentage of participants who report having seen the new tobacco health warnings by data collection wave and smoker type (i.e., buys cigarette packs, buys packs and singles, buys singles).

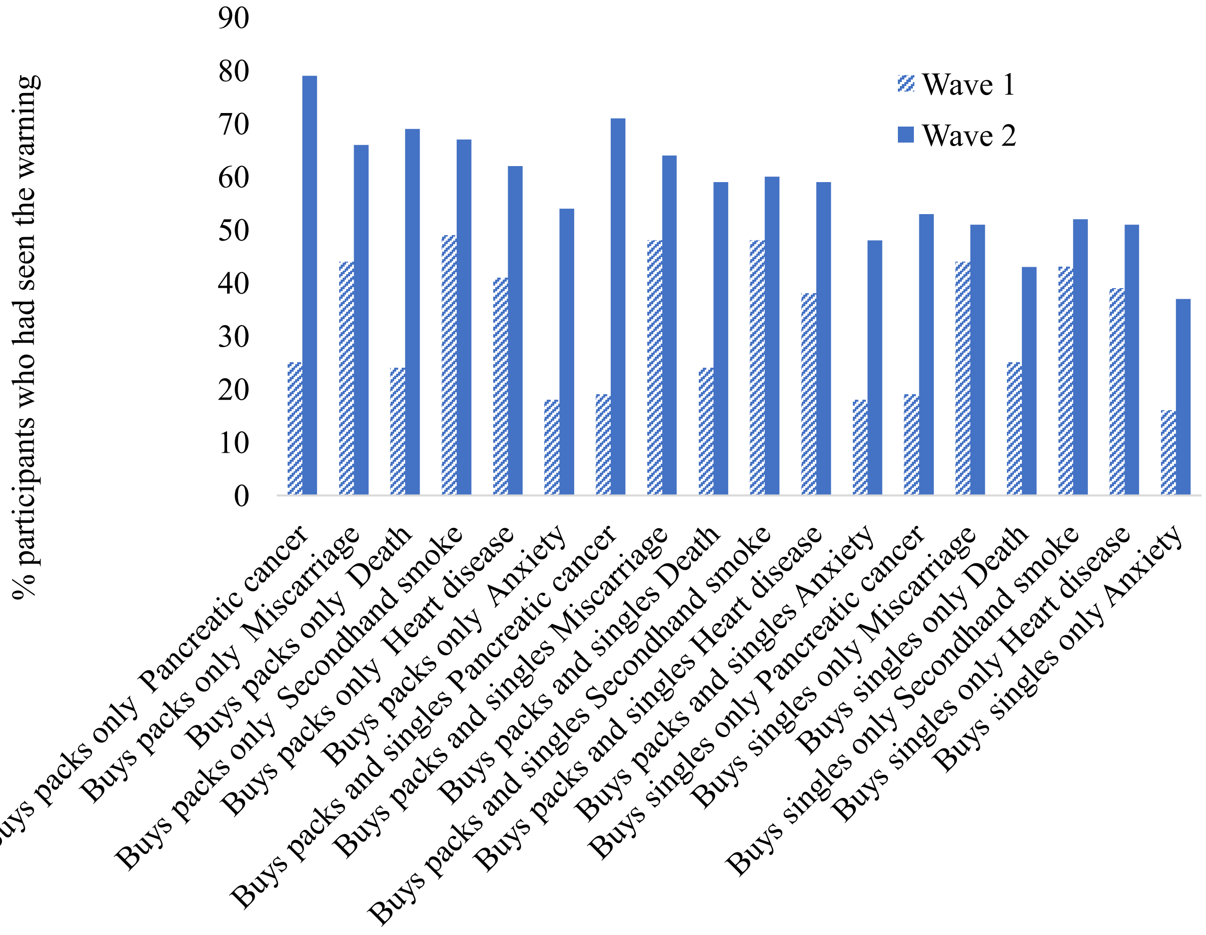

only

Supplementary Material – Figure 3. Percentage of participants who report having seen the new tobacco health warnings by data collection wave and smoking status (i.e., daily smoker, weekly smoker).

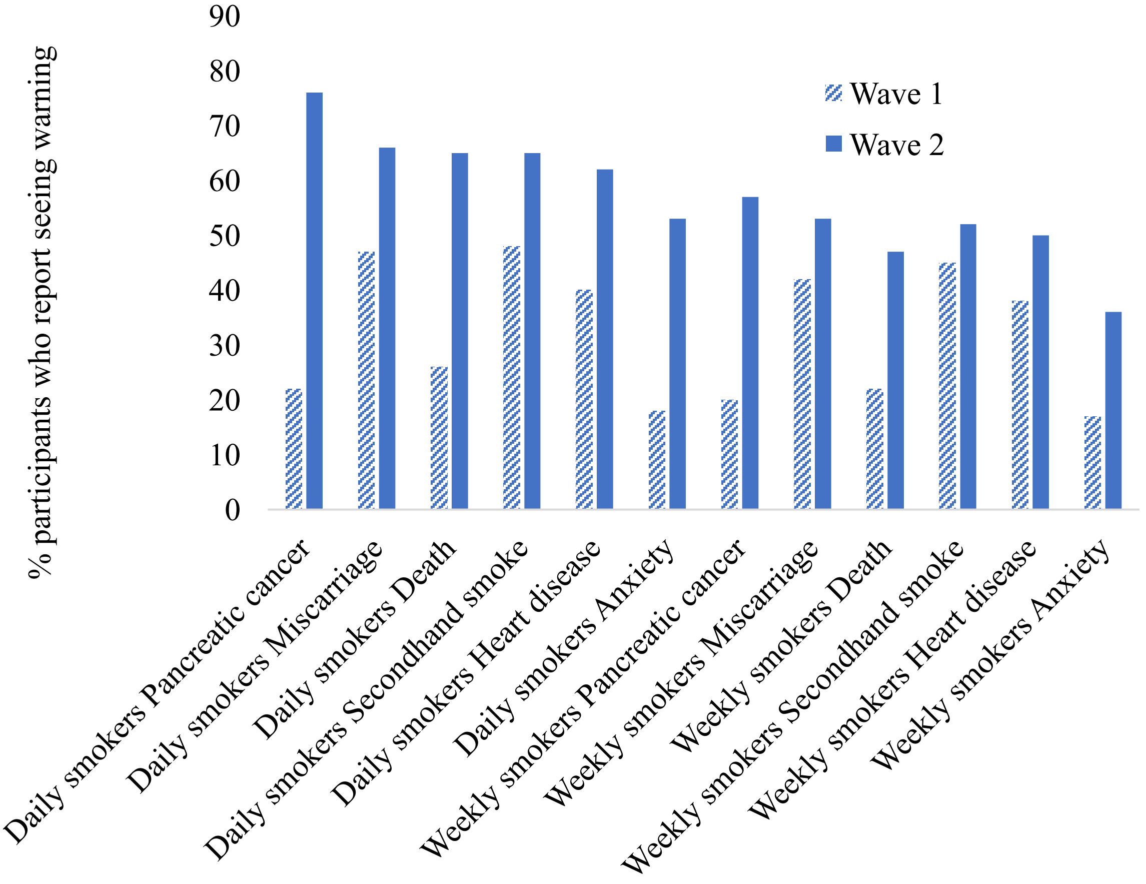

BMJ Open

A cross-sectional online survey of the impact of new tobacco health warnings in Colombia.

Journal:	*BMJ Open*
Manuscript ID	bmjopen-2021-056754.R2
Article Type:	Original research
Date Submitted by the Author:	04-Mar-2022
Complete List of Authors:	Adams, Sally; University of Bath, Psychology<br>Clavijo, Arturo; Universidad Nacional de Colombia, Psychology<br>Tamayo, Ricardo ; Universidad Nacional de Colombia, Psychology<br>Maynard, Olivia ; Univ Bristol, School of Experimental Psychology
**Primary Subject Heading**:	Public health
Secondary Subject Heading:	Global health
Keywords:	Health policy < HEALTH SERVICES ADMINISTRATION & MANAGEMENT, Substance misuse < PSYCHIATRY, PUBLIC HEALTH

SCHOLARONE™
Manuscripts

I, the Submitting Author has the right to grant and does grant on behalf of all authors of the Work (as defined in the below author licence), an exclusive licence and/or a non-exclusive licence for contributions from authors who are: i) UK Crown employees; ii) where BMJ has agreed a CC-BY licence shall apply, and/or iii) in accordance with the terms applicable for US Federal Government officers or employees acting as part of their official duties; on a worldwide, perpetual, irrevocable, royalty-free basis to BMJ Publishing Group Ltd ("BMJ") its licensees and where the relevant Journal is co-owned by BMJ to the co-owners of the Journal, to publish the Work in this journal and any other BMJ products and to exploit all rights, as set out in our licence.

The Submitting Author accepts and understands that any supply made under these terms is made by BMJ to the Submitting Author unless you are acting as an employee on behalf of your employer or a postgraduate student of an affiliated institution which is paying any applicable article publishing charge ("APC") for Open Access articles. Where the Submitting Author wishes to make the Work available on an Open Access basis (and intends to pay the relevant APC), the terms of reuse of such Open Access shall be governed by a Creative Commons licence – details of these licences and which Creative Commons licence will apply to this Work are set out in our licence referred to above.

Other than as permitted in any relevant BMJ Author's Self Archiving Policies, I confirm this Work has not been accepted for publication elsewhere, is not being considered for publication elsewhere and does not duplicate material already published. I confirm all authors consent to publication of this Work and authorise the granting of this licence.

For peer review only

A cross-sectional online survey of the impact of new tobacco health warnings in Colombia.

Sally Adams[1]; Alvaro Arturo Clavijo-Alvarez[2]; Ricardo M Tamayo[2]; Olivia M Maynard[3,4]

[1] Addiction and Mental Health Group (AIM), Department of Psychology, University of Bath, UK

[2] Departamento de Psicología, Universidad Nacional de Colombia, Colombia

[3] School of Psychological Science, University of Bristol, UK

[4] MRC Integrative Epidemiology Unit (IEU), University of Bristol, Bristol, UK

Corresponding author: Sally Adams, Addiction and Mental Health Group, Department of Psychology, University of Bath, 10 West, Bath BA2 7AY, United Kingdom. T: +44. 1225. 384004; F: +44. 1225. 386752; E:S.Adams@bath.ac.uk

Word count: 3,827

For peer review only

ABSTRACT

Objectives This study aimed to explore the impact of a new set of six pictorial warnings introduced in 2018.

Design and Setting Using a cross-sectional design, we examined awareness of the new warnings among Colombian smokers across two time points of data collection.

Participants Adult smokers (≥ 18 years of age), defined as having smoked at least 100 cigarettes in their lifetime and currently smoking at least one cigarette per week participated at Time 1, prior to the introduction of the new health warnings in Colombia in 2018 (n=1985, 72% male), and at Time 2, twelve-months post-introduction (n=1572, 69% male).

Primary Outcomes At each time we examined smokers' responses to warnings on packs (negative affect, thinking about warning messages, cognitive elaboration), attitudes towards smoking (perceived likelihood and severity of harm, self-efficacy, response-efficacy and quit intentions), knowledge of the health risks of smoking and responses to the new warnings (negative affect, believability, thinking about the harms, reactance and perceived message effectiveness).

Results Awareness of the warnings was low, with only 59% of smokers reporting having seen them at Time 2. Between times, we observed a reduction in negative affect towards current warnings ($p < 0.001$), reduced thinking about ($p < 0.001$) and cognitive elaboration of the warning message ($p < 0.001$), and an increase in perceived severity of warnings ($p < 0.001$). When asked about the six new health warnings, we found a reduction in negative affect (ps < 0.07), cognitions related to harm (ps < 0.01), believability (ps < 0.03), reactance (ps < 0.01) and perceived message effectiveness (ps < 0.02) between times.

Conclusions Our data indicate that effectiveness was low prior to the introduction of the new health warnings and at twelve-months post-introduction. Tobacco control policy should seek to improve exposure to and noticeability of tobacco health warnings in Colombia.

For peer review only

Keywords: tobacco health warnings; tobacco control; policy; cross-sectional survey

For peer review only

STRENGTHS AND LIMITATIONS

- Our study includes a large sample size, representing smokers from over 100 different areas in Colombia.
- Our work represents some of the first empirical data examining the impact of tobacco control measures such as warnings in Colombia.
- Our study is limited by the use of self-reported data on the perceived effectiveness of health warnings, where it is difficult to determine whether these responses accurately reflect behaviour
- We were unable to make a direct comparison between participants' responses between study times, as different samples of smokers were recruited. We also observed that many participants failed our attention check, highlighting the limitation of using online self-report data.

For peer review only

INTRODUCTION

Every year, over 20,900 Colombian adults die from tobacco-related diseases and despite this, 10% of the population continue to use tobacco each day.[1] Colombia is an emerging market economy, where tobacco use is a growing public health challenge.[2] In response, Colombia has introduced tobacco control strategies including restrictions on labelling and packaging of tobacco products, smoke-free places, restrictions to advertising and sales.[3]

Warnings on tobacco products are the most cost-effective tool for educating smokers and non-smokers about the health risks of tobacco use.[4] Pictorial warnings (PWs) are more likely to be noticed than text-only warnings,[5-7] are more effective at educating smokers about the health risk of smoking, increasing cognitions about health risks,[5,8] and at increasing motivation to quit smoking,[5,7,9], whilst being less vulnerable to the effects of "wear-out".[8,10] Evidence from low- and middle-income countries indicates that PWs may be more effective in these countries, being one of the few sources of information about tobacco's risks.[4,11,12] Despite this evidence there is high variability in the implementation of warnings in low- and middle-income countries.[13]

PWs were first introduced in Colombia in 2009 and since July 2010 all tobacco products have been required by law to display PWs that cover 30% of the package and are rotated annually. These requirements fall short of the World Health Organisation Framework Convention on Tobacco Control (FCTC) recommendations that warnings cover 50% of the package and that branding is replaced by plain packaging [4]. However, the Colombian Ministry of Health has called for "local evidence" on the effectiveness of larger warnings and plain packaging, before the implementation of these tobacco control measures. In Colombia, smokers' exposure to warnings may be limited. First, implementation is likely to be slow, particularly in rural areas. Each year when new warnings are introduced, retailers have 12 months to stop selling tobacco products with older warnings (referred to here as the 'phase-in period'). Second, there is a high proportion of Colombian smokers who purchase single sticks, rather than packs, who are therefore not exposed to any warnings. Given this uncertainty over the extent to

For peer review only

which Colombian smokers are exposed to new warnings, this one-year cross-sectional study explores smokers' responses to a new set of six pictorial warnings covering 30% of the pack which were introduced in Colombia in 2018.

First, we examined the rate of market saturation and the noticeability of the new warnings among smokers over the implementation period. Given that there is no requirement for retailers to stop selling cigarette packs with the old warnings during the 12-month phase-in period and that many Colombian smokers do not purchase cigarette packs (instead buying single cigarettes), we hypothesised that even a year after implementation, awareness of warnings (a proxy marker of market saturation) will be low (less than 75% of respondents will have seen the warnings). Second, we examined smokers' responses to warnings on packs (negative affect, thinking about warning measures, cognitive elaboration), attitudes towards smoking (perceived likelihood and severity of harm, self-efficacy, response-efficacy and quit intentions), knowledge of the health risks of smoking and responses to the new warnings (negative affect, believability, thinking about the harms, reactance and perceived message effectiveness) across the survey times (baseline and 12-months post introduction). Given relatively small changes between the new and old warnings and a low rate of market saturation, we hypothesised that over our assessment period there would be either a small, or no increase in negative affect, cognitions related to smoking, knowledge regarding health risks of smoking, perceived severity and likelihood of harm from smoking and quit intentions. Third, we examined smokers' responses to each of the new warnings in terms of negative affect, believability, cognitions related to harm, avoidance, reactance and perceived message effectiveness. Due to wear-out (as a result of repeated, albeit potentially limited exposure) of the warnings, we hypothesised that each of these outcomes would be greatest at baseline as compared with 12-months post exposure. Our hypotheses, methods and analysis plan were pre-registered in our study protocol (osf.io/qbr5v).

METHODS

For peer review only

Design

A cross-sectional online survey of Colombian smokers with two times of data collection: one prior to the introduction of the new health warnings, and one 12-months post-introduction. The study was approved by the Universidad Nacional de Colombia, Faculty of Human Sciences Ethics Committee (reference: B.VDIECH-024-18).

Participants

We recruited adult smokers (≥ 18 years of age), defined as having smoked at least 100 cigarettes in their lifetime and currently smoking at least one cigarette per week, from the staff and students at the Universidad Nacional de Colombia and from the general public. The research team used existing mailing lists to contact members of the university across eight cities in Colombia and attended student classes to encourage students to complete the surveys. We recruited participants from the general population through social media, word of mouth and posters in public spaces.

Participants provided their email addresses at baseline. For the second time participants who completed the baseline time were re-contacted to ask if they would like to participate again. We also sent the survey to individuals who may not have participated in the previous times using the methods described above. At each survey time, participants provided their email address and could choose to enter a prize draw to win one of three iPads.

Measures and Materials

The study team translated all questionnaire measures into Spanish. A professional translator also completed a process of 'back-translation'. Following this, we discussed discrepancies and optimal wording and the original Spanish translations were changed. All wording and questions were checked by native Spanish speakers in pre-study piloting.

Health warnings

For peer review only

The six warnings introduced on 21st July 2018 depicted six different health-related consequences of tobacco smoking (miscarriage, pancreatic cancer, heart disease, death, anxiety and secondhand smoke), (see Supplementary Materials - Figure 1).

Smoking behaviour

Participants completed smoking behaviour questions to determine eligibility. Demographic measures also included questions on smoker type and smoking heaviness and the first item of the Fagerström Test for Nicotine Dependence (FTND) [14] (see Supplementary Materials – Table 1).

Awareness of new warnings

Awareness of the new pictorial warnings among Colombian smokers was assessed across two times of data collection.

Responses to warnings seen on tobacco packs

Responses to warnings seen on packs were assessed using measures of negative affect, thinking about warning message and cognitive elaboration.

Attitudes to smoking

Attitudes to smoking were assessed using measures of perceived likelihood of harm, perceived severity of harm from smoking, self-efficacy, response efficacy and quit intentions.

Responses to new warnings

In response to viewing the six new health warnings, participants were asked to complete measures of negative affect, believability, thinking about harms, reactance, perceived message effectiveness.

For peer review only

Knowledge of health risks

We assessed knowledge of the six specific health risks presented on the new health warnings.

Procedure

The first time of data collection occurred between 21st May and 12th July 2018, prior to 21st July 2018, the date at which new warnings could start appearing on the market. The second time of data collection took place between 5th June and 1st August 2019. All procedures were identical at the two times.

Participants accessed the study via Qualtrics and were first shown an information statement before completing a tick-box consent page. All text and questions were presented in Spanish. Participants completed screening questions and those who were ineligible were taken to the end of the study. Eligible participants completed demographic questions, responses to warnings on packs, knowledge of health risks and attitudes towards smoking. An attention check item was included in this section (Please select the strongly agree option to let us know that you read all of the survey instructions). Each of the six new warnings were presented individually on screen in a randomised order, and participants completed questions about each warning. After reading a debriefing page, participants who wished to be entered into the prize draw were directed to a second survey. Participants who participated in the first time were re-contacted at the second time to ask if they would like to participate again.

Data analysis

To examine market spread of new warnings over the implementation period we calculated percentages of participants in the two times who reported being aware of seeing each of the six new warnings. In exploratory analyses we examined how warning awareness differs between smoking status (daily, weekly smokers) and smoker type (buys cigarette packs, buys singles, buys both).

For peer review only

To examine the impact of the new warnings, we compared responses to warnings on packs, knowledge of health risks and attitudes to smoking between Time 1 and Time 2 using independent samples t-tests. We ran these analyses both including and excluding participants who were defined as unexposed to the new warnings at Time 2. We defined participants as 'unexposed' if they reported not seeing (or being unsure whether they had seen) three or more of the six new warnings at Time 2.

To examine smokers' responses to each of the new warnings, we compared negative affect, believability, thinking about the harms, reactance and perceived message effectiveness for each of the six warnings at Time 1 and Time 2 using independent samples t-tests.

The internal consistency of the latent constructs (e.g. negative affect, cognitive elaboration) were assessed using Cronbach's alpha. All latent constructs achieved acceptable to good internal consistency and latent variables were calculated by taking a mean of the individual items (see Supplementary Materials - Table 2).

We conducted analyses using IBM SPSS Statistics 22. The data that form the basis of the results presented here are available from the University of Bath Research Data Repository (TBC).

RESULTS

Data screening and participant characteristics

Data were examined prior to analysis and participants who completed the survey after the closing date (*n*=6 in Time 1) or failed the attention check (*n*=1866 in Time 1 and *n*=988 in Time 2) were removed from further analyses. Participant characteristics are presented by data collection time in Table 1.

[Insert Table 1 about here]

Awareness of new warnings

For peer review only

As expected, participants' awareness of new tobacco health warnings (i.e., participants reporting that they had seen the warning) increased from Time 1 (33%) to Time 2 (59%; see Figure 1). Note that the new warnings had not been introduced on packs by Time 1, so the 33% who reported seeing them were mistaken. Participants mistakenly reporting that they had seen the new warnings at Time 1 was highest for the secondhand smoke (47%), miscarriage (45%) and heart disease (46%) warnings and lowest for the death (25%), pancreatic cancer (21%) and anxiety (18%) warnings.

[Insert Figure 1 about here]

Awareness of the new health warnings at Time 2 was higher for smokers who reported buying only cigarette packs or both pack and single cigarettes, compared with those who bought only single cigarettes. Awareness of the new health warnings at Time 2 was also higher for daily smokers compared with weekly smokers (see Supplementary Materials Figures 2 and 3).

Responses to warnings seen on tobacco packs

As shown in Table 2, there was evidence of a decrease in negative affect, thinking about warning messages and cognitive elaboration between Time 1 and 2. These findings did not differ when including only participants defined as 'exposed' at Time 2.

Attitudes to smoking

There was strong evidence of an increase in perceived severity between Time 1 and 2 (Table 2), although this difference was not evident after including only participants defined as exposed to the warnings at Time 2. The analysis including only participants exposed at Time 2 also indicated strong evidence of a decrease in self-efficacy and quit intention between times. There was no evidence for any other differences.

For peer review only

Knowledge of health risks

There was strong evidence of an increase in mean knowledge of health risks between times across all warnings, when including only participants defined as exposed at Time 2 (see Table 2). For individual warnings, there was evidence of a small, but statistically meaningful increase in knowledge for the health risks “Smoking causes pancreatic cancer” and “Smoking causes anxiety” between times. The analysis including only participants exposed to the warnings at Time 2 indicated that there was also evidence of an increase between times in knowledge for the health risk “Smoking while pregnant could cause you to lose your baby”. These data were highly skewed, however, with high rates of participants giving scores of either 0 (strongly disagree) or 100 (strongly agree) for each knowledge question (See Table 3).

[Insert Tables 2 and 3 about here]

For peer review only

Responses to new warnings

Differences in responses to the six new health warnings are shown in Table 4.

For the health warning "Miscarriage" there was strong evidence of a decrease in negative affect, cognitions related to harm, believability and perceived message effectiveness between times. For the health warning "Pancreatic cancer" there was strong evidence of a decrease in cognitions related to harm and reactance and an increase in believability between times. For the health warning "Anxiety" there was no evidence of any differences in response to the warning. For the health warning "Heart disease" there was strong evidence of a decrease in negative affect, cognitions related to harm and perceived message effectiveness between wavs. For the health warning "Second-hand smoke" there was strong evidence of a decrease in negative affect, cognitions related to harm, believability and perceived message effectiveness between times. Finally, for the health warning "Death" there was strong evidence of a decrease in negative affect, cognitions related to harm and perceived message effectiveness between times. There was no evidence for any other differences.

[Insert Table 4 about here]

DISCUSSION

Every year since 2010, Colombian smokers have been faced with new health warnings covering 30% of their cigarette packs. Using a cross-sectional design, we examined how smokers responded to the new set of warnings introduced in 2018 and find that they had relatively limited impact on knowledge of risk and attitudes towards smoking. We do however find some interesting differences between the warnings themselves, and together these findings have important implications for health warning design and implementation in Colombia and worldwide.

For peer review only

As hypothesised, we found that awareness of the warnings was low. Across the six warnings, the average percentage of participants reporting having seen them at Time 2 was 59%. This is particularly low, given that even at Time 1, 33% of participants incorrectly reported having seen the warnings. Given this low level of awareness, it is unsurprising that we see either no changes, or relatively small changes in our measures of responses to warnings on packs, overall knowledge of health risks and attitudes to smoking between Times 1 and 2 – these are outcomes we would expect to improve, had the new warnings made an impact on smokers. Although the differences were small, contrary to our hypothesis, participants reported *lower* negative affect, thinking about the warnings and cognitive elaboration of the warnings on their cigarette packs at Time 2. Even when we only include those participants we defined as 'exposed' to the new warnings at Time 2, these unexpected effects persisted. We saw little to no change in attitudes to smoking between times, although there was some evidence that participants at Time 2 thought that the severity of smoking harms were greater, and they had greater knowledge of the health risks of smoking. However, again, contrary to expectations, when we only examined these effects among participants who we defined as exposed to the new warnings at Time 2, self-efficacy and quit intentions were lower at Time 2.

Together, these findings suggest that the new warnings had relatively little impact on smokers. We consider there to be a number of complementary explanations for this. First, although Time 2 was conducted 12 months after the new warnings were permitted for sale, it is likely that they did not become commonplace for some time after that, as there is no legal requirement for retailers to stop selling packs with older tobacco warnings until 12 months after the implementation period. It is possible that this slow phase-in diminished warnings' impact and meant that even a year after they (in theory) started being available on packs for sale, few participants recalled having seen them. Second, low awareness of warnings is also

For peer review only

likely related to the extent to which smokers engage with cigarette packs. Despite being prohibited by law, many Colombian smokers purchase single cigarettes, [15] and therefore may not be exposed to health warnings at all. Indeed, we find that awareness of the new warnings is greatest for those who smoke daily and those who buy packs.

We do however find some interesting differences between the warnings themselves. As might be expected, we see the highest levels of participants incorrectly reporting at Time 1 that they had seen the new warnings for those with themes used in previous years ('miscarriage', 'heart disease', 'secondhand smoke' were used most recently in 2017 and 'death' was used in 2015; see Supplementary Materials -Table 3). For these four 'familiar' warnings (similar theme, but different wording and pictorial), we see high levels of believability and knowledge of these health risks at both times and only a small proportion of participants reporting that they 'strongly disagreed' that smoking caused these health risks.

However, for all four of these familiar warnings, we observe reductions between times in almost all specific responses to them (e.g., perceived message effectiveness and negative affect). These data may provide support for previous work which has observed a declining impact of warnings (i.e., "wear-out") over time. [7,16-19]. However, wear-out assumes that the warnings were initially effective, but become less effective with time. The six warnings tested here received relatively low scores on all measures at Time 1 and although statistically significant, the observed declining impact of warnings at Time 2 was small and may not reflect a meaningful change. These low scores for the warnings may suggest that they are not effective at eliciting responses such as negative affect, cognitions related to harm, believability, and perceived message effectiveness and it is possible that their effectiveness may be limited by the theme or disease depicted in the warning.

There are two warning themes which were new on Colombian cigarette packs in 2018, 'pancreatic cancer' and 'anxiety'. As expected, for these novel warnings, we saw a much

For peer review only

smaller percentage of participants incorrectly reporting that they had seen them at Time 1 than the four familiar warnings. However, interestingly, at Time 2, we saw divergent levels of awareness for these two warnings. The largest percentage of participants reported having seen the pancreatic cancer warning of all warnings at Time 2, but the lowest percentage of all participants reported having seen the anxiety warning. We suggest this may be due to the pictorial used, with the pancreatic cancer warning displaying a close-up of a yellow eye, which is arguably novel and memorable, while the anxiety warning depicted a man pulling at his hair. Previous research has suggested that more "severe" or grotesque warnings increase smokers' intentions to quit and are rated as more believable and effective than less severe warnings. [20,21]

Knowledge of the risk of smoking for both pancreatic cancer and anxiety was low at Time 1 and at this time-point we saw the highest percentages of participants reporting that they 'strongly disagreed' that smoking caused these conditions. The inclusion of these new warning messages reflects the priority for the Colombian Ministry of Health to inform the public about "lesser known" smoking health harms. We saw increased knowledge of these two health risks at Time 2, and fewer participants selecting 'strongly disagree' particularly for anxiety, although knowledge was still lower than for most other health risks. Despite this, these increases suggest that novel warnings can impact smokers' knowledge of smoking risks. We also saw that the anxiety warning had the lowest response scores at both times compared with all other warnings (particularly negative affect, believability, perceived message effectiveness and cognitions related to harm). We suggest that these low scores, combined with low 'knowledge' scores, are in line with the inconsistent cross-sectional evidence regarding the relationship between smoking and anxiety,[22] and evidence from Mendelian randomisation studies which find no causal relationship between smoking and the onset of anxiety.[23] We suggest that it is important to consider the veracity of the health risk

For peer review only

claims depicted in health warnings and we raise the important question of whether including health risks which are not supported by the evidence might reduce the overall impact of warnings.

Warnings have repeatedly been shown to be a cost-effective tool for communicating the health risks of smoking to smokers' and non-smokers.[4] For low- and middle-income countries, warnings are proposed to be even more effective.[4] However, for this to occur, smokers must be exposed to warnings, where increased noticeability may improve warning effectiveness. Our data suggest that strategies for increasing exposure and noticeability of warnings are required in Colombia. Currently, warnings in Colombia cover 30% of the pack, with the FCTC recommending that warnings should cover at least 50%. Noticeability and subsequent effectiveness of warnings may be improved by the introduction of larger pictorial health warnings. [24,25]. Additionally, as a large proportion of smokers purchase single cigarettes rather than packs, including health warning messages on individual cigarette sticks represents a novel and effective method for reducing tobacco use.[26-28]

Our study has numerous strengths, including a large sample size, representing smokers from over 100 different areas in Colombia. Additionally, our work represents some of the first empirical data examining the impact of tobacco control measures such as warnings in Colombia. However, our findings should also be considered in light of the following limitations. Our study is limited by the use of self-reported data on the perceived effectiveness of health warnings, where it is difficult to determine whether these responses accurately reflect behaviour. We were unable to make a direct comparison between participants' responses between study times, as different samples of smokers were recruited. We also observed that many participants failed our attention check, highlighting the limitation of using online self-report data. Our participants may not be representative of the general population in Colombia and in particular smokers. Internet penetration is estimated at

For peer review only

65% in Colombia [29], where individuals without internet access would not have been able to participate in our survey. Further, our sample of smokers were on average younger, more educated and more likely to be male compared with Colombian smokers reported in previous research [30]. Therefore, our sample may limit the generalisability of our findings to all smokers in Colombia, where further research on the effectiveness of tobacco health warnings for all Colombian smokers is warranted.

CONCLUSIONS

Health warnings represent a cost-effective tool for communicating the health risks of smoking in low- and middle-income countries. However, our data suggest that current warnings have a limited impact on knowledge of risk and attitudes towards smoking. Future tobacco control policy should seek to improve the noticeability of and exposure to tobacco health warnings in Colombia. Strategies may include consideration of veracity of the health risk claims depicted in health warnings, the inclusion of health warnings on single cigarette sticks and increased warning size on tobacco products.

For peer review only

CONTRIBUTORSHIP

Conception and design of the work: SA, RMT, AACA, OMM. Analysis: SA and OMM. Interpretation of data for the work: all authors. Drafting the work: SA and OMM. Revising it critically: all authors. Final approval of the version to be published: all authors.

FUNDING

This research was funded by an ESRC New Investigator's Award, awarded to Dr Olivia Maynard (ES/R003424/1) and a University of Bristol Global Challenges Research Ad Hoc Fund.

COMPETING INTERESTS

RMTO and AACA were previously hired by the Colombian Ministry of Health to design the six warnings used here. The Ministry of Health had no role in the design of the study.

PATIENT AND PUBLIC INVOLVEMENT

It was not appropriate or possible to involve patients or the public in the design, or conduct, or reporting, or dissemination plans of our research

DATA AVAILABILITY

The data will be made available for sharing via a University of Bristol online data repository

For peer review only

FIGURES

Figure 1. Percentage of participants who report being aware of having seen the new warnings at Wave 1 and 2

TABLES

Table 1. Participant characteristics by data collection time

	***n* = 1985**	***n* = 1572**	***p* -value [CIs]**
Sex (% male)	72	69	N/A
Age (years)	26 (9)	26 (8)	0.03 [-1.098 - 0.049]
Location (% Bogota)	49	67	< 0.001
Smoking frequency			
Daily smokers (%)	65	62	0.06
Cigarettes per day (daily smokers)	7 (6)	7 (5)	N/A
Weekly smokers (%)	35	38	0.06
Cigarettes per week (weekly smokers)	10 (10)	9 (9)	0.09
How many days a week do you smoke? (weekly smokers)	3 (1)	3 (1)	0.42
Cigarettes per day (weekly smokers)	3 (3)	3 (3)	0.38
Type of packs bought			
Buys cigarette packs (%)	38	31	< 0.001
Buys single cigarettes (%)	22	26	N/A
Buys both packs and single cigarettes (%)	39	41	0.21
Dependence			
Smokes within first 60 minutes of waking (%)	27	24	0.02

For peer review only

Note. Values are %, except for age, cigarettes per day, cigarettes per week, smoking days per week -mean (standard deviation). P-values represent differences in variables between times (t-tests/chi-square).

For peer review only

Table 2. Responses to tobacco health warnings and attitudes to smoking by data collection time (for all participants and excluding participants unexposed to new warnings at Time 2).

		**Time 1 vs Time 2 - including all participants at Time 2**			**Time 1 vs Time 2 - only including participants exposed to new warnings at Time 2**				
	**Time 1**	**Time 2**	***t***	df^b	***p* - value**	**Time 2**	***t***	df^b	***p* - value**
	(*n* = 1985)	(*n* = 1572)				(n = 910)			
Responses to warnings on packs									
Negative affect	41(27)	38 (26)	3.03	3411	<0.001	38 (26)	3.35	2893	<0.001
Thinking about warning messages	31 (26)	28 (25)	3.28	3454	<0.001	27 (25)	3.28	1880	<0.001
Cognitive elaboration	46 (31)	42 (29)	3.54	3432	< 0.001	42 (29)	3.59	1867	< 0.001
Knowledge of health risks (overall)	73 (17)	74 (16)	-1.78	3443	0.08	76 (17)	-3.91	2893	< 0.001
Lose your baby[a]	85 (22)	85 (21)	-0.59	3470	0.56	87 (20)	-2.24	1979	0.03
Smoking causes pancreatic cancer	68 (29)	70 (27)	-2.2	3555	0.03	72 (28)	-3.53	2893	< 0.001
Secondhand smoke is deadly	74 (27)	73 (26)	1.2	3555	0.23	74 (26)	0.41	2893	0.68
Smoking damages your heart	83 (21)	83 (20)	-0.03	3555	0.98	84 (20)	-0.9	2893	0.37
Smoking causes anxiety	60 (36)	64 (34)	-3.41	3471	<0.001	67 (33)	-5.67	1948	< 0.001
Smoking causes a slow and painful death	68 (30)	68 (28)	-0.59	3460	0.56	70 (28)	-1.93	1873	0.05
Attitudes to smoking									
Perceived likelihood of harm	78 (19)	77 (17)	0.61	3467	0.54	78 (17)	-0.3	1887	0.76
Perceived severity of smoking harms	87 (17)	89 (15)	-3.97	3501	< 0.001	88 (16)	-1.73	1815	0.08
Self-efficacy	69 (32)	67 (32)	1.65	3555	0.1	64 (33)	3.73	1708	< 0.001
Response-efficacy	83 (24)	84 (22)	-0.54	3487	0.59	84 (22)	-0.32	1899	0.75
Quit intention	60 (30)	59 (30)	1.61	3554	0.11	56 (31)	3.9	2892	< 0.001

For peer review only

Note. Vales are mean (standard deviation), *t*-score, degrees of freedom and *p*-values

[a] Full wording is ‘Smoking while pregnant could cause you to lose your baby’

[b] Degrees of freedom differ according to equality of variances of variables

For peer review only

Table 3. Knowledge of health risks (percentage of participants giving a score of 0 or 100) by data collection time (for all participants and excluding participants unexposed to new warnings at Time 2).

			**Time 1 vs Time 2 including all participants at Time 2**		**Time 1 vs Time 2 - only including participants exposed to new warnings at Time 2**	
	**Time 1**		**Time 2**		**Time 2**	
	(*n* = 1985)		(*n* = 1572)		(n = 910)	
	% giving a score of 0	% giving a score of 100	% giving a score of 0	% giving a score of 100	% giving a score of 0	% giving a score of 100
**Warning 1 "Pancreatic cancer"**	4	24	3	25	3	28
**Warning 2 "Miscarriage"**	1	50	1	48	1	51
**Warning 3 "Death"**	5	25	3	23	3	25
**Warning 4 "Secondhand smoke"**	2	31	1	27	1	30
**Warning 5 "Heart disease"**	1	41	1	39	1	42
**Warning 6 "Anxiety"**	11	25	7	25	6	28

Note. Values are % of participants giving a score of 0 'strongly disagree' or 100 "strongly agree" for the six new warnings

For peer review only

Table 4. Responses to the new tobacco health warnings by data collection time

	Time 1	Time 2	*t*	*df*[a]	*p* -value
	(n = 1985)	(n = 1572)			
**Warning 1 "Pancreatic cancer"**					
Negative affect	47 (30)	45 (28)	1.79	3093	0.07
Cognitions related to harm	59 (32)	57 (32)	2.55	3134	0.01
Believability	57 (32)	60 (31)	-2.97	3076	< 0.001
Reactance	41 (25)	39 (25)	2.52	3134	0.01
Perceived message effectiveness	48 (27)	47 (26)	1.52	3134	0.13
**Warning 2 "Miscarriage"**					
Negative affect	50 (30)	44 (29)	5.57	3128	<0.001
Cognitions related to harm	59 (34)	51 (35)	6.16	2984	<0.001
Believability	67 (31)	64 (31)	2.92	3128	< 0.001
Reactance	43 (27)	42 (27)	0.84	3128	0.4
Perceived message effectiveness	51 (27)	47 (27)	4.1	3128	<0.001
**Warning 3 "Death"**					
Negative affect	55 (30)	51 (29)	3.52	3136	<0.001
Cognitions related to harm	67 (31)	64 (31)	2.75	3136	0.01
Believability	68 (30)	66 (30)	1.5	3136	0.13
Reactance	42 (27)	41 (25)	0.4	3096	0.69
Perceived message effectiveness	54 (26)	52 (27)	2.31	3136	0.02
**Warning 4 "Secondhand smoke"**					
Negative affect	46 (29)	42 (28)	3.52	3123	<0.001
Cognitions related to harm	61 (33)	55 (33)	4.95	3123	<0.001
Believability	69 (30)	66 (30)	2.19	3123	0.03
Reactance	40 (25)	40 (25)	-0.19	3123	0.85
Perceived message effectiveness	48 (26)	45 (26)	3.39	3123	< 0.001
**Warning 5 "Heart disease"**					
Negative affect	49 (30)	45 (28)	3.33	3068	< 0.001
Cognitions related to harm	64 (31)	60 (31)	3.41	3112	< 0.001
Believability	66 (30)	65 (30)	1.6	3112	0.11
Reactance	38 (25)	37 (24)	1.51	3058	0.13

For peer review only

Perceived message effectiveness	49 (26)	46 (25)	3.47	3112	< 0.001
**Warning 6 "Anxiety"**					
Negative affect	30 (28)	29 (26)	1.2	3089	0.23
Cognitions related to harm	42 (35)	41 (33)	1.04	3084	0.3
Believability	51 (34)	53 (34)	-1.4	3131	0.16
Reactance	41 (25)	40 (25)	1.14	3131	0.26
Perceived message effectiveness	36 (25)	35 (25)	0.93	3131	0.35

Note. Values are mean (standard deviation), *t*-score, degrees of freedom and *p*-values

[a] Degrees of freedom differ according to equality of variances of variables

For peer review only

REFERENCES

1 American Cancer Society (2021, April 28). Tobacco Atlas: Colombia https://tobaccoatlas.org/country/colombia/

2 Posada-Villa, J., Cheng, H. G., Martins, S. S., Storr, C. L., Aguilar-Gaxiola, S. A., & Anthony, J. C. (2011). The tobacco dependence dimension in Colombia. *Revista panamericana de salud publica = Pan American journal of public health*, *29*(1), 52–56.

3 Campaign for Tobacco Free Kids (2021, April 28) Legislation by Country: Colombia https://www.tobaccocontrollaws.org/legislation/country/colombia/pl-health-warnings

4 Fong, G. T., Hammond, D., & Hitchman, S. C. (2009). The impact of pictures on the effectiveness of tobacco warnings. *Bulletin of the World Health Organization*, *87*(8), 640–643. https://doi.org/10.2471/blt.09.069575

5 Hammond, D., Fong, G. T., McNeill, A., Borland, R., & Cummings, K. M. (2006). Effectiveness of cigarette warning labels in informing smokers about the risks of smoking: findings from the International Tobacco Control (ITC) Four Country Survey. *Tobacco control*, *15 Suppl 3*(Suppl 3), iii19–iii25. https://doi.org/10.1136/tc.2005.012294

6 Thrasher, J. F., Hammond, D., Fong, G. T., & Arillo-Santillán, E. (2007). Smokers' reactions to cigarette package warnings with graphic imagery and with only text: a comparison between Mexico and Canada. *Salud publica de Mexico*, *49 Suppl 2*, S233–S240. https://doi.org/10.1590/s0036-36342007000800013

For peer review only - http://bmjopen.bmj.com/site/about/guidelines.xhtml

7 Borland, R., Wilson, N., Fong, G. T., Hammond, D., Cummings, K. M., Yong, H. H., Hosking, W., Hastings, G., Thrasher, J., & McNeill, A. (2009a). Impact of graphic and text warnings on cigarette packs: findings from four countries over five years. *Tobacco control*, *18*(5), 358–364. https://doi.org/10.1136/tc.2008.028043

8 Li, J., & Grigg, M. (2009). New Zealand: new graphic warnings encourage registrations with the quitline. *Tobacco control*, *18*(1), 72. https://doi.org/10.1136/tc.2008.027649

9 Borland, R., Yong, H. H., Wilson, N., Fong, G. T., Hammond, D., Cummings, K. M., Hosking, W., & McNeill, A. (2009b). How reactions to cigarette packet health warnings influence quitting: findings from the ITC Four-Country survey. *Addiction (Abingdon, England)*, *104*(4), 669–675. https://doi.org/10.1111/j.1360-0443.2009.02508.x

10 Hammond, D., Fong, G. T., Borland, R., Cummings, K. M., McNeill, A., & Driezen, P. (2007). Text and graphic warnings on cigarette packages: findings from the international tobacco control four country study. *American journal of preventive medicine*, *32*(3), 202–209. https://doi.org/10.1016/j.amepre.2006.11.011

11 Swayampakala, K., Thrasher, J. F., Yong, H. H., Nagelhout, G. E., Li, L., Borland, R., Hammond, D., O'Connor, R. J., & Hardin, J. W. (2018). Over-Time Impacts of Pictorial Health Warning Labels and their Differences across Smoker Subgroups: Results from Adult Smokers in Canada and Australia. *Nicotine & tobacco research: official journal of the Society for Research on Nicotine and Tobacco*, *20*(7), 888–896. https://doi.org/10.1093/ntr/ntx134

For peer review only

12 FCTC (2009) article 11: tobacco warning labels: evidence and recommendations from the ITC project Waterloo, ON: International Tobacco Control Policy Evaluation Project. http://www.itcproject.org

13 Wipfli, H. L., Fujimoto, K., & Valente, T. W. (2010). Global tobacco control diffusion: the case of the framework convention on tobacco control. *American journal of public health*, *100*(7), 1260–1266. https://doi.org/10.2105/AJPH.2009.167833

14 Heatherton, T. F., Kozlowski, L. T., Frecker, R. C., & Fagerström, K. O. (1991). The Fagerström Test for Nicotine Dependence: a revision of the Fagerström Tolerance Questionnaire. *British journal of addiction*, *86*(9), 1119–1127. https://doi.org/10.1111/j.1360-0443.1991.tb01879.x

15 Maldonado, N., Llorente, B., Escobar, D., et al (2020). Smoke signals: monitoring illicit cigarettes and smoking behaviour in Colombia to support tobacco taxes. *Tobacco Control,* 29, 243-248.

16 Woelbert, E., d'Hombres, B. (2019). Pictorial health warnings and wear-out effects: evidence from a web experiment in 10 European countries. *Tobacco Control,* 28, 71-76.

17 Environics Research Group (1999) Canadian adult and youth opinions on the sizing of health warning messages. Ottawa, Canada.

18 Environics Research Group (2000) Testing new health warning messages for cigarette packages: A summary of three phases of focus group research: Final report. Ottawa, Canada.

19 Brubaker, R. G., & Mitby, S. K. (1990). Health-risk warning labels on smokeless tobacco products: are they effective?. *Addictive behaviors*, *15*(2), 115–118. https://doi.org/10.1016/0306-4603(90)90014-o

20 Maynard, O.M., Gove, H., Skinner, A.L. *et al.* (2018) Severity and susceptibility: measuring the perceived effectiveness and believability of tobacco health warnings. *BMC Public Health* 18**,** 468. https://doi.org/10.1186/s12889-018-5385-x

21 Kees, J., Burton, S., Andrews, J. C., & Kozup, J. (2010). Understanding how Graphic Pictorial Warnings Work on Cigarette Packaging. *Journal of Public Policy & Marketing*, *29*(2), 265–276. https://doi.org/10.1509/jppm.29.2.265

22 Fluharty, M., Taylor, A. E., Grabski, M., & Munafò, M. R. (2017). The Association of Cigarette Smoking with Depression and Anxiety: A Systematic Review. *Nicotine & tobacco research: official journal of the Society for Research on Nicotine and Tobacco*, *19*(1), 3–13. https://doi.org/10.1093/ntr/ntw140

23 Taylor, A. E., Fluharty, M. E., Bjørngaard, J. H., Gabrielsen, M. E., Skorpen, F., Marioni, R. E., Campbell, A., Engmann, J., Mirza, S. S., Loukola, A., Laatikainen, T., Partonen, T., Kaakinen, M., Ducci, F., Cavadino, A., Husemoen, L., Ahluwalia, T. S., Jacobsen, R. K., Skaaby, T., Ebstrup, J. F., … Munafò, M. R. (2014). Investigating the possible causal association of smoking with depression and anxiety using Mendelian randomisation meta-

For peer review only

analysis: the CARTA consortium. *BMJ open*, *4*(10), e006141. https://doi.org/10.1136/bmjopen-2014-006141

24 Anshari, D., Yong, H., Borland, R., et al (2018). Which type of tobacco product warning imagery is more effective and sustainable over time? A longitudinal assessment of smokers in Canada, Australia and Mexico. *BMJ Open, 8*. doi: 10.1136/bmjopen-2018-021983

25 Li, L., Borland, R., Yong, H., Cummings, K. M., Thrasher, J. F., Hitchman, S. C., Fong, G. T., Hammond, D., & Bansal-Travers, M. (2015). Longer term impact of cigarette package warnings in Australia compared with the United Kingdom and Canada. *Health education research*, *30*(1), 67–80. https://doi.org/10.1093/her/cyu074

26 Drovandi, A., Teague, P.A., Glass, B., Malau-Aduli, B. (2019) Do health warnings on cigarette sticks dissuade smokers and non-smokers? A focus group and interview study of Australian university students. *Psychol Res Behav Manag*. 12:361-373 https://doi.org/10.2147/PRBM.S193754

27 Moodie, C.S., Hiscock, R,. Thrasher, J. *et al.* (2018) Perceptions of cigarette pack inserts promoting cessation and dissuasive cigarettes among young adult smokers in the UK: a cross-sectional online survey. *BMJ Open* 8:e019662. doi: 10.1136/bmjopen-2017-019662

28 Moodie, C., Gendall, P., Hoek, J., MacKintosh, A. M., Best, C., & Murray, S. (2019). The Response of Young Adult Smokers and Nonsmokers in the United Kingdom to Dissuasive Cigarettes: An Online Survey. *Nicotine & tobacco research : official journal of the Society for Research on Nicotine and Tobacco*, *21*(2), 227–233. https://doi.org/10.1093/ntr/ntx261

For peer review only - http://bmjopen.bmj.com/site/about/guidelines.xhtml

29 Statista. (2022, January 19) Percentage of population using the internet in Colombia from 2000 to 2019. https://www.statista.com/statistics/209109/number-of-internet-users-per-100-inhabitants-in-colombia-since-2000/

30 Storr, C. L., Cheng, H., Posada-Villa, J., Aguilar-Gaxiola, S., & Anthony, J. C. (2008). Adult smokers in Colombia: who isn't giving it up?. *Addictive behaviors*, *33*(3), 412–421. https://doi.org/10.1016/j.addbeh.2007.10.003

For peer review only

Figure 1. Percentage of participants who report being aware of having seen the new warnings at Wave 1 and 2

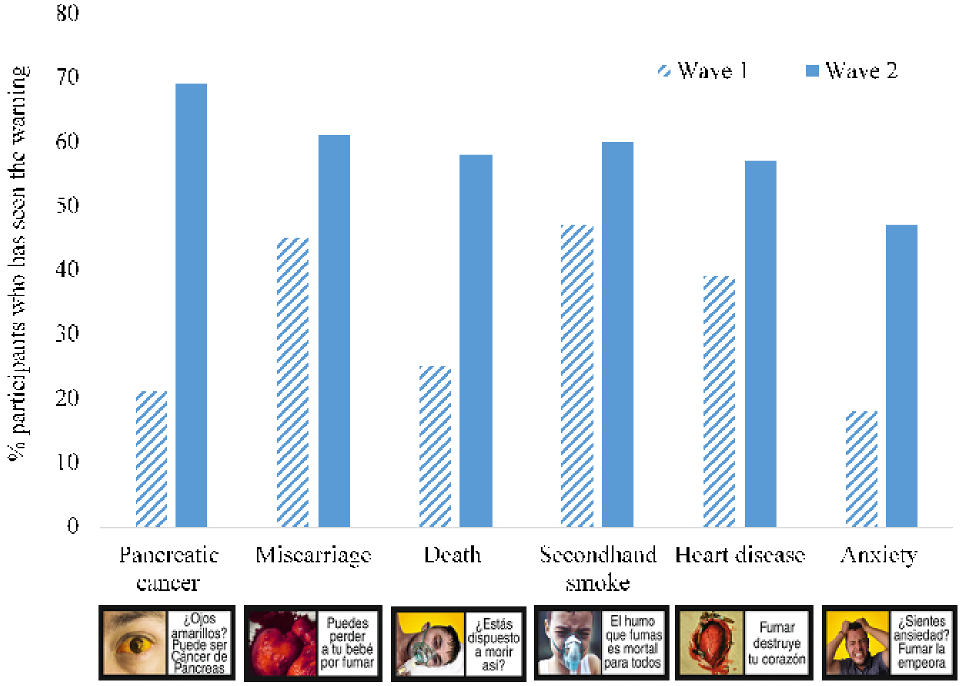

185x153mm (96 x 96 DPI)

Supplementary Materials Table 1. Survey Questions

Variable	Items	Response
Smoking behaviour - eligibility	Have you smoked at least 100 cigarettes in your lifetime?	1 = Yes<br>2 = No
	How often do you smoke cigarettes?	1 = Every day<br>2 = Every week<br>3 = Less than every week<br>4 = Not al all
Smoking heaviness – daily smokers	Approximately how many cigarettes do you smoke per day?	Provide a single number
Smoking heaviness -non-daily smokers	Approximately how many days a week do you smoke	
	On the days that you do smoke, approximately how many cigarettes do you smoke	
	Approximately how many cigarettes do you smoke per week?	
Fagerström Test for Nicotine Dependence (FTND)	How soon after you wake do you smoke your first cigarette?	1 = Within 5 minutes<br>2 = 6 to 30 minutes<br>3 = 31 to 60 minutes<br>4 = After 60 minutes
Awareness of new warnings	Have you seen this health warning on a cigarette pack?	1= Yes<br>2 = No<br>3 = Unsure
Responses to warnings seen on tobacco packs		
Negative affect	In the last week, the warning on my cigarette pack made me feel...	
	…scared	0 = Strongly disagree<br>25 = Somewhat disagree<br>50 = Neither agree nor disagree<br>75 = Somewhat agree<br>100 = Strongly agree
	…regretful	
	…disgusted	

Thinking about the warning message	When I notice my cigarette pack, I think about the message that the warning conveys	0 = Strongly disagree<br>25 = Somewhat disagree<br>50 = Neither agree nor disagree<br>75 = Somewhat agree<br>100 = Strongly agree
	When my cigarette pack is not<br>in sight, I think about the message that the warning conveys	
Attitudes towards smoking		
Perceived likelihood of harm	What is the chance that you will one day get the following diseases if you continue to smoke cigarettes?	
	…heart disease?	0 = No chance<br>25 = Low chance<br>50 = Moderate chance<br>75 = High chance<br>100 = Certain
	…cancer?	
	…a permanent breathing problem?	
Perceived severity of harm from smoking	How much would getting the following diseases because of smoking affect your life?	
	…heart disease?	0 = No chance<br>25 = Low chance<br>50 = Moderate chance<br>75 = High chance<br>100 = Certain
	…cancer?	
	…a permanent breathing problem?	
Self-efficacy	I see myself as being capable of quitting smoking in the next 2 months if I wanted to	0 = Strongly disagree<br>25 = Somewhat disagree<br>50 = Neither agree nor disagree<br>75 = Somewhat agree<br>100 = Strongly agree
Response-efficacy	Quitting smoking would lower my chances of getting a<br>disease related<br>to smoking	

Quit intentions	Are you planning to quit smoking within the next 6 months?	0 = Very unlikely<br>25 = Unlikely<br>50 = Maybe, maybe not<br>75 = Likely<br>100 = Very likely
**Responses to new warnings**		
Negative affect	This warning makes me feel…	
	…scared	0 = Strongly disagree<br>25 = Somewhat disagree<br>50 = Neither agree nor disagree<br>75 = Somewhat agree<br>100 = Strongly agree
	…regretful	
	…disgusted	
Believability	This warning is believable	0 = Strongly disagree<br>25 = Somewhat disagree<br>50 = Neither agree nor disagree<br>75 = Somewhat agree<br>100 = Strongly agree
Thinking about harms	This warning makes me think about the health problems caused by smoking	0 = Strongly disagree<br>25 = Somewhat disagree<br>50 = Neither agree nor disagree<br>75 = Somewhat agree<br>100 = Strongly agree
Reactance	This warning is trying to manipulate me	0 = Strongly disagree<br>25 = Somewhat disagree<br>50 = Neither agree nor disagree<br>75 = Somewhat agree<br>100 = Strongly agree
	The health effect of this warning is overblown	
	This warning annoys me	
Perceived message effectiveness	This warning discourages me from wanting to smoke	0 = Strongly disagree<br>25 = Somewhat disagree<br>50 = Neither agree nor disagree<br>75 = Somewhat agree<br>100 = Strongly agree
	This warning makes smoking seem unpleasant to me	
	This warning makes me concerned about the health effects of smoking	
	If I saw this warning on my	

For peer review only

	cigarette pack, I would...	
	...try to avoid thinking about it	
	…try to avoid looking at it	
	…keep the pack out of sight to avoid looking at the warning	
Knowledge of health risks	Say how much you agree or disagree with each statement below:	0 = Strongly disagree<br>25 = Somewhat disagree<br>50 = Neither agree nor disagree<br>75 = Somewhat agree<br>100 = Strongly agree
	Smoking while pregnant could cause you to lose your baby	
	Smoking causes pancreatic cancer	
	Secondhand smoke is deadly	
	Smoking damages your heart	
	Smoking causes anxiety	
	Smoking causes a slow and painful death	

Supplementary Material – Table 2. Internal consistency of latent constructs

**Latent construct**	**Number of items**	**α**	***r***
Responses to warnings on packs			
Negative affect	3 items	0.79	0.49 – 0.69
Thinking about warning message	2 items	0.66	0.5
Cognitive elaboration	2 items	0.8	0.66
Attitudes to smoking			
Perceived likelihood of harm	3 items	0.84	0.61-0.65
Perceived severity	3 items	0.87	0.66-0.71
Responses to new warnings			
**Warning 1 "Pancreatic cancer"**			
Negative affect	3 items	0.83	0.54 – 0.77
Reactance	3 items	0.68	0.39 – 0.45
Perceived effectiveness	6 items	0.87	0.34 – 0.82
**Warning 2 "Miscarriage"**			
Negative affect	3 items	0.77	0.38 – 0.79
Reactance	3 items	0.69	0.41 – 0.44
Perceived effectiveness	6 items	0.86	0.28 – 0.75
**Warning 3 "Death"**			
Negative affect	3 items	0.8	0.46 – 0.77
Reactance	3 items	0.69	0.43 – 0.44
Perceived effectiveness	6 items	0.86	0.33 – 0.82
**Warning 4 "Secondhand Smoke"**			
Negative affect	3 items	0.81	0.49 – 0.67
Reactance	3 items	0.67	0.37 – 0.44
Perceived effectiveness	6 items	0.86	0.31 – 0.80
**Warning 5 "Heart disease"**			
Negative affect	3 items	0.83	0.53 – 0.80
Reactance	3 items	0.68	0.40 – 0.44
Perceived effectiveness	6 items	0.86	0.33 – 0.80
**Warning 6 "Anxiety"**			
Negative affect	3 items	0.87	0.63 – 0.76
Reactance	3 items	0.64	0.33 – 0.43
Perceived effectiveness	6 items	0.87	0.34 – 0.85

For peer review only

Note. The internal consistency of latent constructs assessed using Cronbach's alpha and associations between the constructs using bivariate correlations.

For peer review only

Supplementary Materials Table 3. – Presence of warning theme between 2010 and 2018.

Warning theme	2010	2011	2012	2013	2014	2015	2016	2017	2018
Death							X		X
Secondhand smoke		X			X			X	X
Anxiety									X
Miscarriage		X		X			X	X	X
Heart attack / heart disease					X		X	X	X
Pancreatic cancer									X
Mouth cancer and bad breath	X								
Kidney cancer				X				X	
Breast cancer				X					
Leukemia					X				
Bladder cancer					X				
Laryngeal cancer							X		
Cancer							X		
Damaged arteries					X			X	
Thrombosis					X				
Vascular problems / disease				X					
Cerebral stroke		X							
Lung disease		X					X		
Eye problems / disease		X							
Bone damage				X					
Sexual impotence	X	X		X					
Aging	X								

Data are taken from https://www.tobaccocontrollaws.org/legislation/country/colombia/laws. Data not available for 2012 or 2015.

All warnings are available from the Colombian Ministry of Health and Social Protection.

Colombia. Ministerio de Salud y Protección Social. (2020). Advertencias sanitarias productos de tabaco y derivados 2010 - 2021. [Piezas publicitarias;] Retrieved From: https://www.minsalud.gov.co/sites/rid/Lists/BibliotecaDigital/RIDE/VS/PP/ENT/advertencias-sanitarias-todas-vigencias.zip

For peer review only

Supplementary Material - Figure 1. Six new tobacco health warnings introduced in Colombia July 2018

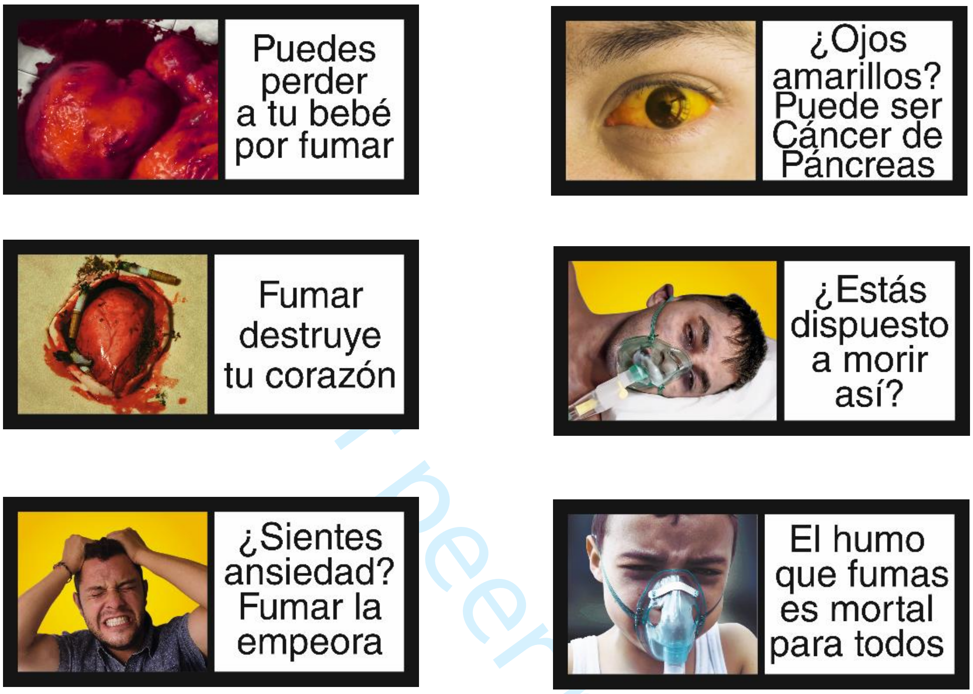

Health warning topics – clockwise *"You can lose your baby by smoking", "Yellow eyes? It could be pancreatic cancer", "Smoking destroys your heart", "Are you willing to die like this?", "Do you feel anxiety? Smoking makes it worse"* and *"The smoke you smoke is deadly for everyone".*

Supplementary Material – Figure 2. Percentage of participants who report having seen the new tobacco health warnings by data collection wave and smoker type (i.e., buys cigarette packs, buys packs and singles, buys singles).

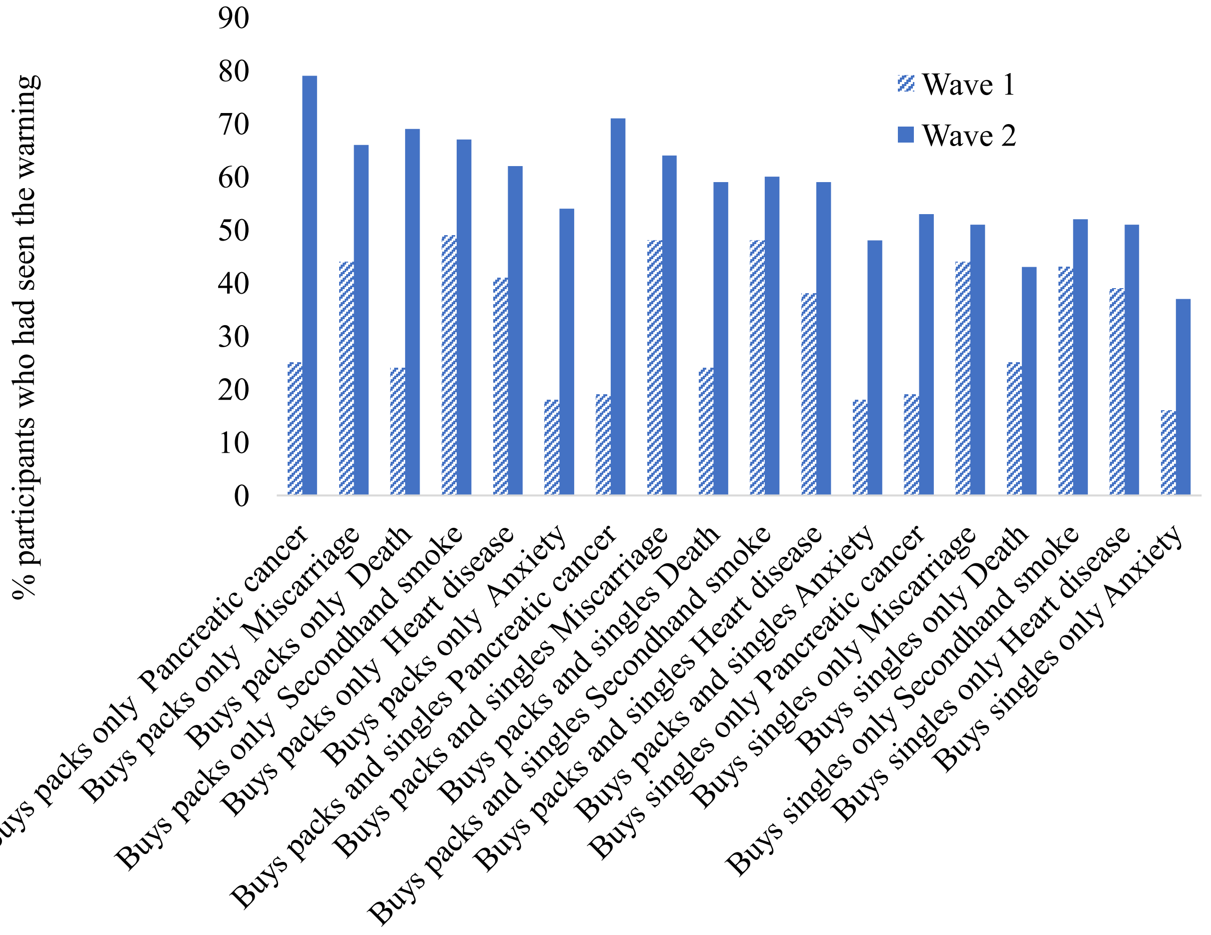

Supplementary Material – Figure 3. Percentage of participants who report having seen the new tobacco health warnings by data collection wave and smoking status (i.e., daily smoker, weekly smoker).

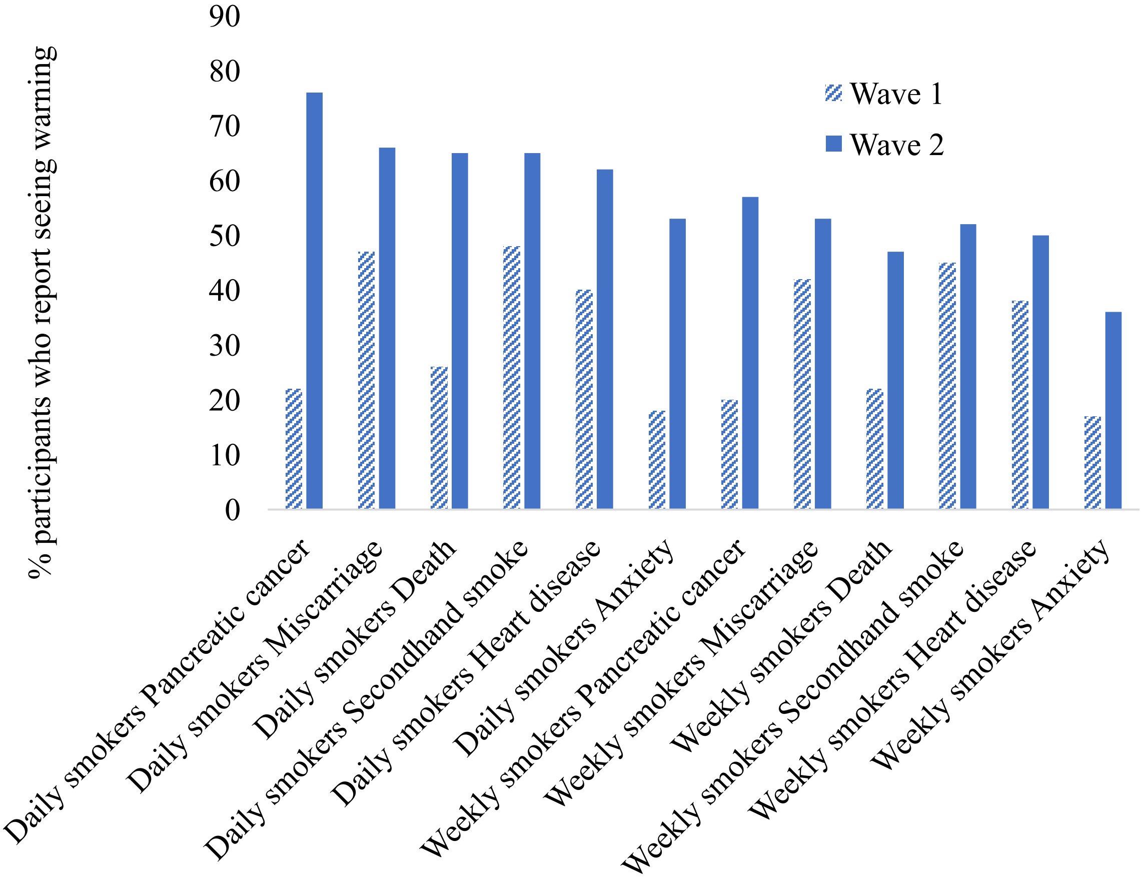

BMJ Open

A cross-sectional online survey of the impact of new tobacco health warnings in Colombia.

Journal:	*BMJ Open*
Manuscript ID	bmjopen-2021-056754.R3
Article Type:	Original research
Date Submitted by the Author:	07-Apr-2022
Complete List of Authors:	Adams, Sally; University of Bath, Psychology<br>Clavijo, Arturo; Universidad Nacional de Colombia, Psychology<br>Tamayo, Ricardo ; Universidad Nacional de Colombia, Psychology<br>Maynard, Olivia ; Univ Bristol, School of Experimental Psychology
**Primary Subject Heading**:	Public health
Secondary Subject Heading:	Global health
Keywords:	Health policy < HEALTH SERVICES ADMINISTRATION & MANAGEMENT, Substance misuse < PSYCHIATRY, PUBLIC HEALTH

SCHOLARONE™
Manuscripts

I, the Submitting Author has the right to grant and does grant on behalf of all authors of the Work (as defined in the below author licence), an exclusive licence and/or a non-exclusive licence for contributions from authors who are: i) UK Crown employees; ii) where BMJ has agreed a CC-BY licence shall apply, and/or iii) in accordance with the terms applicable for US Federal Government officers or employees acting as part of their official duties; on a worldwide, perpetual, irrevocable, royalty-free basis to BMJ Publishing Group Ltd ("BMJ") its licensees and where the relevant Journal is co-owned by BMJ to the co-owners of the Journal, to publish the Work in this journal and any other BMJ products and to exploit all rights, as set out in our licence.

The Submitting Author accepts and understands that any supply made under these terms is made by BMJ to the Submitting Author unless you are acting as an employee on behalf of your employer or a postgraduate student of an affiliated institution which is paying any applicable article publishing charge ("APC") for Open Access articles. Where the Submitting Author wishes to make the Work available on an Open Access basis (and intends to pay the relevant APC), the terms of reuse of such Open Access shall be governed by a Creative Commons licence – details of these licences and which Creative Commons licence will apply to this Work are set out in our licence referred to above.

Other than as permitted in any relevant BMJ Author's Self Archiving Policies, I confirm this Work has not been accepted for publication elsewhere, is not being considered for publication elsewhere and does not duplicate material already published. I confirm all authors consent to publication of this Work and authorise the granting of this licence.

For peer review only

A cross-sectional online survey of the impact of new tobacco health warnings in Colombia.

Sally Adams[1]; Alvaro Arturo Clavijo-Alvarez[2]; Ricardo M Tamayo[2]; Olivia M Maynard[3,4]

[1] Addiction and Mental Health Group (AIM), Department of Psychology, University of Bath, UK

[2] Departamento de Psicología, Universidad Nacional de Colombia, Colombia

[3] School of Psychological Science, University of Bristol, UK

[4] MRC Integrative Epidemiology Unit (IEU), University of Bristol, Bristol, UK

Corresponding author: Sally Adams, Addiction and Mental Health Group, Department of Psychology, University of Bath, 10 West, Bath BA2 7AY, United Kingdom. T: +44. 1225. 384004; F: +44. 1225. 386752; E:S.Adams@bath.ac.uk

Word count: 3,871

For peer review only

ABSTRACT

Objectives This study aimed to explore the impact of a new set of six pictorial warnings introduced in 2018.

Design and Setting Using a cross-sectional design, we examined awareness of the new warnings among Colombian smokers across two time points of data collection.

Participants Adult smokers (≥ 18 years of age), defined as having smoked at least 100 cigarettes in their lifetime and currently smoking at least one cigarette per week participated at Time 1, prior to the introduction of the new health warnings in Colombia in 2018 (n=1985, 72% male), and at Time 2, twelve-months post-introduction (n=1572, 69% male).

Primary Outcomes At each time we examined smokers' responses to warnings on packs (negative affect, thinking about warning messages, cognitive elaboration), attitudes towards smoking (perceived likelihood and severity of harm, self-efficacy, response-efficacy and quit intentions), knowledge of the health risks of smoking and responses to the new warnings (negative affect, believability, thinking about the harms, reactance and perceived message effectiveness).

Results Awareness of the warnings was low, with only 59% of smokers reporting having seen them at Time 2. Between times, we observed a reduction in negative affect towards current warnings ($p < 0.001$), reduced thinking about ($p < 0.001$) and cognitive elaboration of the warning message ($p < 0.001$), and an increase in perceived severity of warnings ($p < 0.001$). When asked about the six new health warnings, we found a reduction in negative affect (ps < 0.07), cognitions related to harm (ps < 0.01), believability (ps < 0.03), reactance (ps < 0.01) and perceived message effectiveness (ps < 0.02) between times.

Conclusions Our data indicate that effectiveness was low prior to the introduction of the new health warnings and at twelve-months post-introduction. Tobacco control policy should seek to improve exposure to and noticeability of tobacco health warnings in Colombia.

For peer review only

Keywords: tobacco health warnings; tobacco control; policy; cross-sectional survey

For peer review only

STRENGTHS AND LIMITATIONS

- Our study includes a large sample size, representing smokers from over 100 different areas in Colombia.
- Our work represents some of the first empirical data examining the impact of tobacco control measures such as warnings in Colombia.
- Our study is limited by the use of self-reported data on the perceived effectiveness of health warnings, where it is difficult to determine whether these responses accurately reflect behaviour
- We were unable to make a direct comparison between participants' responses between study times, as different samples of smokers were recruited. We also observed that many participants failed our attention check, highlighting the limitation of using online self-report data.

For peer review only

INTRODUCTION

Every year, over 20,900 Colombian adults die from tobacco-related diseases and despite this, 10% of the population continue to use tobacco each day.[1] Colombia is an emerging market economy, where tobacco use is a growing public health challenge.[2] In response, Colombia has introduced tobacco control strategies including restrictions on labelling and packaging of tobacco products, smoke-free places, restrictions to advertising and sales.[3]

Warnings on tobacco products are the most cost-effective tool for educating smokers and non-smokers about the health risks of tobacco use.[4] Pictorial warnings (PWs) are more likely to be noticed than text-only warnings,[5-7] are more effective at educating smokers about the health risk of smoking, increasing cognitions about health risks,[5,8] and at increasing motivation to quit smoking,[5,7,9], whilst being less vulnerable to the effects of "wear-out".[8,10] Evidence from low- and middle-income countries indicates that PWs may be more effective in these countries, being one of the few sources of information about tobacco's risks.[4,11,12] Despite this evidence there is high variability in the implementation of warnings in low- and middle-income countries.[13]

PWs were first introduced in Colombia in 2009 and since July 2010 all tobacco products have been required by law to display PWs that cover 30% of the package and are rotated annually. These requirements fall short of the World Health Organisation Framework Convention on Tobacco Control (FCTC) recommendations that warnings cover 50% of the package and that branding is replaced by plain packaging [4]. However, the Colombian Ministry of Health has called for "local evidence" on the effectiveness of larger warnings and plain packaging, before the implementation of these tobacco control measures. In Colombia, smokers' exposure to warnings may be limited. First, implementation is likely to be slow, particularly in rural areas. Each year when new warnings are introduced, retailers have 12 months to stop selling tobacco products with older warnings (referred to here as the 'phase-in period'). Second, there is a high proportion of Colombian smokers who purchase single sticks, rather than packs, who are therefore not exposed to any warnings. Given this uncertainty over the extent to

For peer review only - http://bmjopen.bmj.com/site/about/guidelines.xhtml

which Colombian smokers are exposed to new warnings, this one-year cross-sectional study explores smokers' responses to a new set of six pictorial warnings covering 30% of the pack which were introduced in Colombia in 2018.

First, we examined the rate of market saturation and the noticeability of the new warnings among smokers over the implementation period. Given that there is no requirement for retailers to stop selling cigarette packs with the old warnings during the 12-month phase-in period and that many Colombian smokers do not purchase cigarette packs (instead buying single cigarettes), we hypothesised that even a year after implementation, awareness of warnings (a proxy marker of market saturation) will be low (less than 75% of respondents will have seen the warnings). Second, we examined smokers' responses to warnings on packs (negative affect, thinking about warning measures, cognitive elaboration), attitudes towards smoking (perceived likelihood and severity of harm, self-efficacy, response-efficacy and quit intentions), knowledge of the health risks of smoking and responses to the new warnings (negative affect, believability, thinking about the harms, reactance and perceived message effectiveness) across the survey times (baseline and 12-months post introduction). Given relatively small changes between the new and old warnings and a low rate of market saturation, we hypothesised that over our assessment period there would be either a small, or no increase in negative affect, cognitions related to smoking, knowledge regarding health risks of smoking, perceived severity and likelihood of harm from smoking and quit intentions. Third, we examined smokers' responses to each of the new warnings in terms of negative affect, believability, cognitions related to harm, avoidance, reactance and perceived message effectiveness. Due to wear-out (as a result of repeated, albeit potentially limited exposure) of the warnings, we hypothesised that each of these outcomes would be greatest at baseline as compared with 12-months post exposure. Our hypotheses, methods and analysis plan were pre-registered in our study protocol (osf.io/qbr5v).

METHODS

For peer review only

Design

A cross-sectional online survey of Colombian smokers with two times of data collection: one prior to the introduction of the new health warnings, and one 12-months post-introduction. The study was approved by the Universidad Nacional de Colombia, Faculty of Human Sciences Ethics Committee (reference: B.VDIECH-024-18).

Participants

We recruited adult smokers (≥ 18 years of age), defined as having smoked at least 100 cigarettes in their lifetime and currently smoking at least one cigarette per week, from the staff and students at the Universidad Nacional de Colombia and from the general public. The research team used existing mailing lists to contact members of the university across eight cities in Colombia and attended student classes to encourage students to complete the surveys. We recruited participants from the general population through social media, word of mouth and posters in public spaces. Due to the exploratory nature of the study we did not conduct a sample size calculation.

Participants provided their email addresses at baseline. For the second time participants who completed the baseline time were re-contacted to ask if they would like to participate again. We also sent the survey to individuals who may not have participated in the previous times using the methods described above. At each survey time, participants provided their email address and could choose to enter a prize draw to win one of three iPads.

Measures and Materials

The study team translated all questionnaire measures into Spanish. A professional translator also completed a process of 'back-translation'. Following this, we discussed discrepancies and optimal wording and the original Spanish translations were changed. All wording and questions were checked by native Spanish speakers in pre-study piloting.

For peer review only

Health warnings

The six warnings introduced on 21st July 2018 depicted six different health-related consequences of tobacco smoking (miscarriage, pancreatic cancer, heart disease, death, anxiety and secondhand smoke), (see Supplementary Materials - Figure 1).

Smoking behaviour

Participants completed smoking behaviour questions to determine eligibility. Demographic measures also included questions on smoker type and smoking heaviness and the first item of the Fagerström Test for Nicotine Dependence (FTND) [14] (see Supplementary Materials – Table 1).

Awareness of new warnings

Awareness of the new pictorial warnings among Colombian smokers was assessed across two times of data collection.

Responses to warnings seen on tobacco packs

Responses to warnings seen on packs were assessed using measures of negative affect, thinking about warning message and cognitive elaboration.

Attitudes to smoking

Attitudes to smoking were assessed using measures of perceived likelihood of harm, perceived severity of harm from smoking, self-efficacy, response efficacy and quit intentions.

Responses to new warnings

In response to viewing the six new health warnings, participants were asked to complete measures of negative affect, believability, thinking about harms, reactance, perceived message effectiveness.

For peer review only

Knowledge of health risks

We assessed knowledge of the six specific health risks presented on the new health warnings.

Procedure

The first time of data collection occurred between 21st May and 12th July 2018, prior to 21st July 2018, the date at which new warnings could start appearing on the market. The second time of data collection took place between 5th June and 1st August 2019. All procedures were identical at the two times.

Participants accessed the study via Qualtrics and were first shown an information statement before completing a tick-box consent page. All text and questions were presented in Spanish. Participants completed screening questions and those who were ineligible were taken to the end of the study. Eligible participants completed demographic questions, responses to warnings on packs, knowledge of health risks and attitudes towards smoking. An attention check item was included in this section (Please select the strongly agree option to let us know that you read all of the survey instructions). Each of the six new warnings were presented individually on screen in a randomised order, and participants completed questions about each warning. After reading a debriefing page, participants who wished to be entered into the prize draw were directed to a second survey. Participants who participated in the first time were re-contacted at the second time to ask if they would like to participate again.

Data analysis

To examine market spread of new warnings over the implementation period we calculated percentages of participants in the two times who reported being aware of seeing each of the six new warnings. In exploratory analyses we examined how warning awareness differs between smoking status (daily, weekly smokers) and smoker type (buys cigarette packs, buys singles, buys both).

For peer review only

To examine the impact of the new warnings, we compared responses to warnings on packs, knowledge of health risks and attitudes to smoking between Time 1 and Time 2 using independent samples t-tests. We ran these analyses both including and excluding participants who were defined as unexposed to the new warnings at Time 2. We defined participants as ‘unexposed’ if they reported not seeing (or being unsure whether they had seen) three or more of the six new warnings at Time 2.

To examine smokers’ responses to each of the new warnings, we compared negative affect, believability, thinking about the harms, reactance and perceived message effectiveness for each of the six warnings at Time 1 and Time 2 using independent samples t-tests.

The internal consistency of the latent constructs (e.g. negative affect, cognitive elaboration) were assessed using Cronbach’s alpha. All latent constructs achieved acceptable to good internal consistency and latent variables were calculated by taking a mean of the individual items (see Supplementary Materials - Table 2).

We conducted analyses using IBM SPSS Statistics 22. The data that form the basis of the results presented here are available from the University of Bristol data repository, data.bris, at https://doi.org/10.5523/bris.3fscvnkvn2s2e2ev9vint0lbj3.These data were generated by the authors of this manuscript [dataset] [15].

PATIENT AND PUBLIC INVOLVEMENT

It was not appropriate or possible to involve patients or the public in the design, or conduct, or reporting, or dissemination plans of our research

RESULTS

Data screening and participant characteristics

Data were examined prior to analysis and participants who completed the survey after the closing date (n=6 in Time 1) or failed the attention check (n=1866 in Time 1 and n=988 in Time 2) were removed

For peer review only

from further analyses. Participant characteristics are presented by data collection time in Table 1.

[Insert Table 1 about here]

Awareness of new warnings

As expected, participants' awareness of new tobacco health warnings (i.e., participants reporting that they had seen the warning) increased from Time 1 (33%) to Time 2 (59%; see Figure 1). Note that the new warnings had not been introduced on packs by Time 1, so the 33% who reported seeing them were mistaken. Participants mistakenly reporting that they had seen the new warnings at Time 1 was highest for the secondhand smoke (47%), miscarriage (45%) and heart disease (46%) warnings and lowest for the death (25%), pancreatic cancer (21%) and anxiety (18%) warnings.

[Insert Figure 1 about here]

Awareness of the new health warnings at Time 2 was higher for smokers who reported buying only cigarette packs or both pack and single cigarettes, compared with those who bought only single cigarettes. Awareness of the new health warnings at Time 2 was also higher for daily smokers compared with weekly smokers (see Supplementary Materials Figures 2 and 3).

Responses to warnings seen on tobacco packs

As shown in Table 2, there was evidence of a decrease in negative affect, thinking about warning messages and cognitive elaboration between Time 1 and 2. These findings did not differ when including only participants defined as 'exposed' at Time 2.

Attitudes to smoking

For peer review only

There was strong evidence of an increase in perceived severity between Time 1 and 2 (Table 2), although this difference was not evident after including only participants defined as exposed to the warnings at Time 2. The analysis including only participants exposed at Time 2 also indicated strong evidence of a decrease in self-efficacy and quit intention between times. There was no evidence for any other differences.

Knowledge of health risks

There was strong evidence of an increase in mean knowledge of health risks between times across all warnings, when including only participants defined as exposed at Time 2 (see Table 2). For individual warnings, there was evidence of a small, but statistically meaningful increase in knowledge for the health risks "Smoking causes pancreatic cancer" and "Smoking causes anxiety" between times. The analysis including only participants exposed to the warnings at Time 2 indicated that there was also evidence of an increase between times in knowledge for the health risk "Smoking while pregnant could cause you to lose your baby". These data were highly skewed, however, with high rates of participants giving scores of either 0 (strongly disagree) or 100 (strongly agree) for each knowledge question (See Table 3).

[Insert Tables 2 and 3 about here]

For peer review only

Responses to new warnings

Differences in responses to the six new health warnings are shown in Table 4.

For the health warning "Miscarriage" there was strong evidence of a decrease in negative affect, cognitions related to harm, believability and perceived message effectiveness between times. For the health warning "Pancreatic cancer" there was strong evidence of a decrease in cognitions related to harm and reactance and an increase in believability between times. For the health warning "Anxiety" there was no evidence of any differences in response to the warning. For the health warning "Heart disease" there was strong evidence of a decrease in negative affect, cognitions related to harm and perceived message effectiveness between wavs. For the health warning "Second-hand smoke" there was strong evidence of a decrease in negative affect, cognitions related to harm, believability and perceived message effectiveness between times. Finally, for the health warning "Death" there was strong evidence of a decrease in negative affect, cognitions related to harm and perceived message effectiveness between times. There was no evidence for any other differences.

[Insert Table 4 about here]

DISCUSSION

Every year since 2010, Colombian smokers have been faced with new health warnings covering 30% of their cigarette packs. Using a cross-sectional design, we examined how smokers responded to the new set of warnings introduced in 2018 and find that they had relatively limited impact on knowledge of risk and attitudes towards smoking. We do however find some interesting differences between the warnings themselves, and together these findings have important implications for health warning design and implementation in Colombia and worldwide.

For peer review only

As hypothesised, we found that awareness of the warnings was low. Across the six warnings, the average percentage of participants reporting having seen them at Time 2 was 59%. This is particularly low, given that even at Time 1, 33% of participants incorrectly reported having seen the warnings. Given this low level of awareness, it is unsurprising that we see either no changes, or relatively small changes in our measures of responses to warnings on packs, overall knowledge of health risks and attitudes to smoking between Times 1 and 2 – these are outcomes we would expect to improve, had the new warnings made an impact on smokers. Although the differences were small, contrary to our hypothesis, participants reported *lower* negative affect, thinking about the warnings and cognitive elaboration of the warnings on their cigarette packs at Time 2. Even when we only include those participants we defined as ‘exposed’ to the new warnings at Time 2, these unexpected effects persisted. We saw little to no change in attitudes to smoking between times, although there was some evidence that participants at Time 2 thought that the severity of smoking harms were greater, and they had greater knowledge of the health risks of smoking. However, again, contrary to expectations, when we only examined these effects among participants who we defined as exposed to the new warnings at Time 2, self-efficacy and quit intentions were lower at Time 2.

Together, these findings suggest that the new warnings had relatively little impact on smokers. We consider there to be a number of complementary explanations for this. First, although Time 2 was conducted 12 months after the new warnings were permitted for sale, it is likely that they did not become commonplace for some time after that, as there is no legal requirement for retailers to stop selling packs with older tobacco warnings until 12 months after the implementation period. It is possible that this slow phase-in diminished warnings’ impact and meant that even a year after they (in theory) started being available on packs for sale, few participants recalled having seen them. Second, low awareness of warnings is also

likely related to the extent to which smokers engage with cigarette packs. Despite being prohibited by law, many Colombian smokers purchase single cigarettes, [16] and therefore may not be exposed to health warnings at all. Indeed, we find that awareness of the new warnings is greatest for those who smoke daily and those who buy packs.

We do however find some interesting differences between the warnings themselves. As might be expected, we see the highest levels of participants incorrectly reporting at Time 1 that they had seen the new warnings for those with themes used in previous years ('miscarriage', 'heart disease', 'secondhand smoke' were used most recently in 2017 and 'death' was used in 2015; see Supplementary Materials -Table 3). For these four 'familiar' warnings (similar theme, but different wording and pictorial), we see high levels of believability and knowledge of these health risks at both times and only a small proportion of participants reporting that they 'strongly disagreed' that smoking caused these health risks.

However, for all four of these familiar warnings, we observe reductions between times in almost all specific responses to them (e.g., perceived message effectiveness and negative affect). These data may provide support for previous work which has observed a declining impact of warnings (i.e., "wear-out") over time. [7,17-20]. However, wear-out assumes that the warnings were initially effective, but become less effective with time. The six warnings tested here received relatively low scores on all measures at Time 1 and although statistically significant, the observed declining impact of warnings at Time 2 was small and may not reflect a meaningful change. These low scores for the warnings may suggest that they are not effective at eliciting responses such as negative affect, cognitions related to harm, believability, and perceived message effectiveness and it is possible that their effectiveness may be limited by the theme or disease depicted in the warning.

There are two warning themes which were new on Colombian cigarette packs in 2018, 'pancreatic cancer' and 'anxiety'. As expected, for these novel warnings, we saw a much

For peer review only - http://bmjopen.bmj.com/site/about/guidelines.xhtml

smaller percentage of participants incorrectly reporting that they had seen them at Time 1 than the four familiar warnings. However, interestingly, at Time 2, we saw divergent levels of awareness for these two warnings. The largest percentage of participants reported having seen the pancreatic cancer warning of all warnings at Time 2, but the lowest percentage of all participants reported having seen the anxiety warning. We suggest this may be due to the pictorial used, with the pancreatic cancer warning displaying a close-up of a yellow eye, which is arguably novel and memorable, while the anxiety warning depicted a man pulling at his hair. Previous research has suggested that more "severe" or grotesque warnings increase smokers' intentions to quit and are rated as more believable and effective than less severe warnings. [21,22]

Knowledge of the risk of smoking for both pancreatic cancer and anxiety was low at Time 1 and at this time-point we saw the highest percentages of participants reporting that they 'strongly disagreed' that smoking caused these conditions. The inclusion of these new warning messages reflects the priority for the Colombian Ministry of Health to inform the public about "lesser known" smoking health harms. We saw increased knowledge of these two health risks at Time 2, and fewer participants selecting 'strongly disagree' particularly for anxiety, although knowledge was still lower than for most other health risks. Despite this, these increases suggest that novel warnings can impact smokers' knowledge of smoking risks. We also saw that the anxiety warning had the lowest response scores at both times compared with all other warnings (particularly negative affect, believability, perceived message effectiveness and cognitions related to harm). We suggest that these low scores, combined with low 'knowledge' scores, are in line with the inconsistent cross-sectional evidence regarding the relationship between smoking and anxiety,[23] and evidence from Mendelian randomisation studies which find no causal relationship between smoking and the onset of anxiety.[24] We suggest that it is important to consider the veracity of the health risk

For peer review only

claims depicted in health warnings and we raise the important question of whether including health risks which are not supported by the evidence might reduce the overall impact of warnings.

Warnings have repeatedly been shown to be a cost-effective tool for communicating the health risks of smoking to smokers' and non-smokers.[4] For low- and middle-income countries, warnings are proposed to be even more effective.[4] However, for this to occur, smokers must be exposed to warnings, where increased noticeability may improve warning effectiveness. Our data suggest that strategies for increasing exposure and noticeability of warnings are required in Colombia. Currently, warnings in Colombia cover 30% of the pack, with the FCTC recommending that warnings should cover at least 50%. Noticeability and subsequent effectiveness of warnings may be improved by the introduction of larger pictorial health warnings. [25,26]. Additionally, as a large proportion of smokers purchase single cigarettes rather than packs, including health warning messages on individual cigarette sticks represents a novel and effective method for reducing tobacco use.[27-29]

Our study has numerous strengths, including a large sample size, representing smokers from over 100 different areas in Colombia. Additionally, our work represents some of the first empirical data examining the impact of tobacco control measures such as warnings in Colombia. However, our findings should also be considered in light of the following limitations. Our study is limited by the use of self-reported data on the perceived effectiveness of health warnings, where it is difficult to determine whether these responses accurately reflect behaviour. We were unable to make a direct comparison between participants' responses between study times, as different samples of smokers were recruited. We also observed that many participants failed our attention check, highlighting the limitation of using online self-report data. Our participants may not be representative of the general population in Colombia and in particular smokers. Internet penetration is estimated at

For peer review only

65% in Colombia [30], where individuals without internet access would not have been able to participate in our survey. Further, our sample of smokers were on average younger, more educated and more likely to be male compared with Colombian smokers reported in previous research [31]. Therefore, our sample may limit the generalisability of our findings to all smokers in Colombia, where further research on the effectiveness of tobacco health warnings for all Colombian smokers is warranted.

CONCLUSIONS

Health warnings represent a cost-effective tool for communicating the health risks of smoking in low- and middle-income countries. However, our data suggest that current warnings have a limited impact on knowledge of risk and attitudes towards smoking. Future tobacco control policy should seek to improve the noticeability of and exposure to tobacco health warnings in Colombia. Strategies may include consideration of veracity of the health risk claims depicted in health warnings, the inclusion of health warnings on single cigarette sticks and increased warning size on tobacco products.

For peer review only

CONTRIBUTORSHIP

Conception and design of the work: SA, RMT, AACA, OMM. Analysis: SA and OMM. Interpretation of data for the work: all authors. Drafting the work: SA and OMM. Revising it critically: all authors. Final approval of the version to be published: all authors.

FUNDING

This research was funded by an ESRC New Investigator's Award, awarded to Dr Olivia Maynard (ES/R003424/1) and a University of Bristol Global Challenges Research Ad Hoc Fund.

COMPETING INTERESTS

RMTO and AACA were previously hired by the Colombian Ministry of Health to design the six warnings used here. The Ministry of Health had no role in the design of the study.

DATA AVAILABILITY

The data that form the basis of the results presented here are available from the University of Bristol data repository, data.bris, at https://doi.org/10.5523/bris.3fscvnkvn2s2e2ev9vint0lbj3.These data were generated by the authors of this manuscript [dataset] [15].

For peer review only

FIGURES

Figure 1. Percentage of participants who report being aware of having seen the new warnings at Wave 1 and 2

TABLES

Table 1. Participant characteristics by data collection time

	n **= 1985**	***n*** **= 1572**	***p*** **-value [CIs]**
Sex (% male)	72	69	N/A
Age (years)	26 (9)	26 (8)	0.03 [-1.098 - 0.049]
Location (% Bogota)	49	67	< 0.001
Smoking frequency			
Daily smokers (%)	65	62	0.06
Cigarettes per day (daily smokers)	7 (6)	7 (5)	N/A
Weekly smokers (%)	35	38	0.06
Cigarettes per week (weekly smokers)	10 (10)	9 (9)	0.09
How many days a week do you smoke? (weekly smokers)	3 (1)	3 (1)	0.42
Cigarettes per day (weekly smokers)	3 (3)	3 (3)	0.38
Type of packs bought			
Buys cigarette packs (%)	38	31	< 0.001
Buys single cigarettes (%)	22	26	N/A
Buys both packs and single cigarettes (%)	39	41	0.21
Dependence			
Smokes within first 60 minutes of waking (%)	27	24	0.02

For peer review only

Note. Values are %, except for age, cigarettes per day, cigarettes per week, smoking days per week -mean (standard deviation). P-values represent differences in variables between times (t-tests/chi-square).

For peer review only

Table 2. Responses to tobacco health warnings and attitudes to smoking by data collection time (for all participants and excluding participants unexposed to new warnings at Time 2).

		**Time 1 vs Time 2 - including all participants at Time 2**			**Time 1 vs Time 2 - only including participants exposed to new warnings at Time 2**				
	**Time 1**	**Time 2**	***t***	***df*[b]**	***p* - value**	**Time 2**	***t***	***df*[b]**	***p* - value**
	(*n* = 1985)	(*n* = 1572)				(n = 910)			
Responses to warnings on packs									
Negative affect	41(27)	38 (26)	3.03	3411	<0.001	38 (26)	3.35	2893	<0.001
Thinking about warning messages	31 (26)	28 (25)	3.28	3454	<0.001	27 (25)	3.28	1880	<0.001
Cognitive elaboration	46 (31)	42 (29)	3.54	3432	< 0.001	42 (29)	3.59	1867	< 0.001
Knowledge of health risks (overall)	73 (17)	74 (16)	-1.78	3443	0.08	76 (17)	-3.91	2893	< 0.001
Lose your baby[a]	85 (22)	85 (21)	-0.59	3470	0.56	87 (20)	-2.24	1979	0.03
Smoking causes pancreatic cancer	68 (29)	70 (27)	-2.2	3555	0.03	72 (28)	-3.53	2893	< 0.001
Secondhand smoke is deadly	74 (27)	73 (26)	1.2	3555	0.23	74 (26)	0.41	2893	0.68
Smoking damages your heart	83 (21)	83 (20)	-0.03	3555	0.98	84 (20)	-0.9	2893	0.37
Smoking causes anxiety	60 (36)	64 (34)	-3.41	3471	<0.001	67 (33)	-5.67	1948	< 0.001
Smoking causes a slow and painful death	68 (30)	68 (28)	-0.59	3460	0.56	70 (28)	-1.93	1873	0.05
Attitudes to smoking									
Perceived likelihood of harm	78 (19)	77 (17)	0.61	3467	0.54	78 (17)	-0.3	1887	0.76
Perceived severity of smoking harms	87 (17)	89 (15)	-3.97	3501	< 0.001	88 (16)	-1.73	1815	0.08
Self-efficacy	69 (32)	67 (32)	1.65	3555	0.1	64 (33)	3.73	1708	< 0.001
Response-efficacy	83 (24)	84 (22)	-0.54	3487	0.59	84 (22)	-0.32	1899	0.75
Quit intention	60 (30)	59 (30)	1.61	3554	0.11	56 (31)	3.9	2892	< 0.001

For peer review only

Note. Vales are mean (standard deviation), *t*-score, degrees of freedom and *p*-values

[a] Full wording is 'Smoking while pregnant could cause you to lose your baby'

[b] Degrees of freedom differ according to equality of variances of variables

For peer review only

Table 3. Knowledge of health risks (percentage of participants giving a score of 0 or 100) by data collection time (for all participants and excluding participants unexposed to new warnings at Time 2).

			**Time 1 vs Time 2 including all participants at Time 2**		**Time 1 vs Time 2 - only including participants exposed to new warnings at Time 2**	
	**Time 1**		**Time 2**		**Time 2**	
	(*n* = 1985)		(*n* = 1572)		(n = 910)	
	% giving a score of 0	% giving a score of 100	% giving a score of 0	% giving a score of 100	% giving a score of 0	% giving a score of 100
**Warning 1 "Pancreatic cancer"**	4	24	3	25	3	28
**Warning 2 "Miscarriage"**	1	50	1	48	1	51
**Warning 3 "Death"**	5	25	3	23	3	25
**Warning 4 "Secondhand smoke"**	2	31	1	27	1	30
**Warning 5 "Heart disease"**	1	41	1	39	1	42
**Warning 6 "Anxiety"**	11	25	7	25	6	28

Note. Values are % of participants giving a score of 0 ‘strongly disagree’ or 100 “strongly agree” for the six new warnings

For peer review only

Table 4. Responses to the new tobacco health warnings by data collection time

	Time 1	Time 2	*t*	*df*[a]	*p*-value
	(*n* = 1985)	(*n* = 1572)			
**Warning 1 "Pancreatic cancer"**					
Negative affect	47 (30)	45 (28)	1.79	3093	0.07
Cognitions related to harm	59 (32)	57 (32)	2.55	3134	0.01
Believability	57 (32)	60 (31)	-2.97	3076	< 0.001
Reactance	41 (25)	39 (25)	2.52	3134	0.01
Perceived message effectiveness	48 (27)	47 (26)	1.52	3134	0.13
**Warning 2 "Miscarriage"**					
Negative affect	50 (30)	44 (29)	5.57	3128	<0.001
Cognitions related to harm	59 (34)	51 (35)	6.16	2984	<0.001
Believability	67 (31)	64 (31)	2.92	3128	< 0.001
Reactance	43 (27)	42 (27)	0.84	3128	0.4
Perceived message effectiveness	51 (27)	47 (27)	4.1	3128	<0.001
**Warning 3 "Death"**					
Negative affect	55 (30)	51 (29)	3.52	3136	<0.001
Cognitions related to harm	67 (31)	64 (31)	2.75	3136	0.01
Believability	68 (30)	66 (30)	1.5	3136	0.13
Reactance	42 (27)	41 (25)	0.4	3096	0.69
Perceived message effectiveness	54 (26)	52 (27)	2.31	3136	0.02
**Warning 4 "Secondhand smoke"**					
Negative affect	46 (29)	42 (28)	3.52	3123	<0.001
Cognitions related to harm	61 (33)	55 (33)	4.95	3123	<0.001
Believability	69 (30)	66 (30)	2.19	3123	0.03
Reactance	40 (25)	40 (25)	-0.19	3123	0.85
Perceived message effectiveness	48 (26)	45 (26)	3.39	3123	< 0.001
**Warning 5 "Heart disease"**					
Negative affect	49 (30)	45 (28)	3.33	3068	< 0.001
Cognitions related to harm	64 (31)	60 (31)	3.41	3112	< 0.001
Believability	66 (30)	65 (30)	1.6	3112	0.11
Reactance	38 (25)	37 (24)	1.51	3058	0.13

For peer review only

Perceived message effectiveness	49 (26)	46 (25)	3.47	3112	< 0.001
**Warning 6 "Anxiety"**					
Negative affect	30 (28)	29 (26)	1.2	3089	0.23
Cognitions related to harm	42 (35)	41 (33)	1.04	3084	0.3
Believability	51 (34)	53 (34)	-1.4	3131	0.16
Reactance	41 (25)	40 (25)	1.14	3131	0.26
Perceived message effectiveness	36 (25)	35 (25)	0.93	3131	0.35

For peer review only

Note. Values are mean (standard deviation), *t*-score, degrees of freedom and *p*-values

[a] Degrees of freedom differ according to equality of variances of variables

REFERENCES

1 American Cancer Society (2021, April 28). Tobacco Atlas: Colombia https://tobaccoatlas.org/country/colombia/

2 Posada-Villa, J., Cheng, H. G., Martins, S. S., Storr, C. L., Aguilar-Gaxiola, S. A., & Anthony, J. C. (2011). The tobacco dependence dimension in Colombia. *Revista panamericana de salud publica = Pan American journal of public health*, *29*(1), 52–56.

3 Campaign for Tobacco Free Kids (2021, April 28) Legislation by Country: Colombia https://www.tobaccocontrollaws.org/legislation/country/colombia/pl-health-warnings

4 Fong, G. T., Hammond, D., & Hitchman, S. C. (2009). The impact of pictures on the effectiveness of tobacco warnings. *Bulletin of the World Health Organization*, *87*(8), 640–643. https://doi.org/10.2471/blt.09.069575

5 Hammond, D., Fong, G. T., McNeill, A., Borland, R., & Cummings, K. M. (2006). Effectiveness of cigarette warning labels in informing smokers about the risks of smoking: findings from the International Tobacco Control (ITC) Four Country Survey. *Tobacco control*, *15 Suppl 3*(Suppl 3), iii19–iii25. https://doi.org/10.1136/tc.2005.012294

6 Thrasher, J. F., Hammond, D., Fong, G. T., & Arillo-Santillán, E. (2007). Smokers' reactions to cigarette package warnings with graphic imagery and with only text: a comparison between Mexico and Canada. *Salud publica de Mexico*, *49 Suppl 2*, S233–S240. https://doi.org/10.1590/s0036-36342007000800013

For peer review only

7 Borland, R., Wilson, N., Fong, G. T., Hammond, D., Cummings, K. M., Yong, H. H., Hosking, W., Hastings, G., Thrasher, J., & McNeill, A. (2009a). Impact of graphic and text warnings on cigarette packs: findings from four countries over five years. *Tobacco control*, *18*(5), 358–364. https://doi.org/10.1136/tc.2008.028043

8 Li, J., & Grigg, M. (2009). New Zealand: new graphic warnings encourage registrations with the quitline. *Tobacco control*, *18*(1), 72. https://doi.org/10.1136/tc.2008.027649

9 Borland, R., Yong, H. H., Wilson, N., Fong, G. T., Hammond, D., Cummings, K. M., Hosking, W., & McNeill, A. (2009b). How reactions to cigarette packet health warnings influence quitting: findings from the ITC Four-Country survey. *Addiction (Abingdon, England)*, *104*(4), 669–675. https://doi.org/10.1111/j.1360-0443.2009.02508.x

10 Hammond, D., Fong, G. T., Borland, R., Cummings, K. M., McNeill, A., & Driezen, P. (2007). Text and graphic warnings on cigarette packages: findings from the international tobacco control four country study. *American journal of preventive medicine*, *32*(3), 202–209. https://doi.org/10.1016/j.amepre.2006.11.011

11 Swayampakala, K., Thrasher, J. F., Yong, H. H., Nagelhout, G. E., Li, L., Borland, R., Hammond, D., O'Connor, R. J., & Hardin, J. W. (2018). Over-Time Impacts of Pictorial Health Warning Labels and their Differences across Smoker Subgroups: Results from Adult Smokers in Canada and Australia. *Nicotine & tobacco research: official journal of the Society for Research on Nicotine and Tobacco*, *20*(7), 888–896. https://doi.org/10.1093/ntr/ntx134

For peer review only

12 FCTC (2009) article 11: tobacco warning labels: evidence and recommendations from the ITC project Waterloo, ON: International Tobacco Control Policy Evaluation Project. http://www.itcproject.org

13 Wipfli, H. L., Fujimoto, K., & Valente, T. W. (2010). Global tobacco control diffusion: the case of the framework convention on tobacco control. *American journal of public health*, *100*(7), 1260–1266. https://doi.org/10.2105/AJPH.2009.167833

14 Heatherton, T. F., Kozlowski, L. T., Frecker, R. C., & Fagerström, K. O. (1991). The Fagerström Test for Nicotine Dependence: a revision of the Fagerström Tolerance Questionnaire. *British journal of addiction*, *86*(9), 1119–1127. https://doi.org/10.1111/j.1360-0443.1991.tb01879.x

[dataset] 15 Adams, S., Clavijo-Alvarez, A.A., Tamayo, R., Maynard, O.M. Data from: A cross-sectional online survey of the impact of new tobacco health warnings in Colombia. University of Bristol repository, June 1st 2022. https://doi.org/10.5523/bris.3fscvnkvn2s2e2ev9vint0lbj3.

16 Maldonado, N., Llorente, B., Escobar, D., et al (2020). Smoke signals: monitoring illicit cigarettes and smoking behaviour in Colombia to support tobacco taxes. *Tobacco Control,* 29, 243-248.

17 Woelbert, E., d'Hombres, B. (2019). Pictorial health warnings and wear-out effects: evidence from a web experiment in 10 European countries. *Tobacco Control,* 28, 71-76.

For peer review only

18 Environics Research Group (1999) Canadian adult and youth opinions on the sizing of health warning messages. Ottawa, Canada.

19 Environics Research Group (2000) Testing new health warning messages for cigarette packages: A summary of three phases of focus group research: Final report. Ottawa, Canada.

20 Brubaker, R. G., & Mitby, S. K. (1990). Health-risk warning labels on smokeless tobacco products: are they effective?. *Addictive behaviors*, *15*(2), 115–118. https://doi.org/10.1016/0306-4603(90)90014-o

21 Maynard, O.M., Gove, H., Skinner, A.L. *et al.* (2018) Severity and susceptibility: measuring the perceived effectiveness and believability of tobacco health warnings. *BMC Public Health* 18**,** 468. https://doi.org/10.1186/s12889-018-5385-x

22 Kees, J., Burton, S., Andrews, J. C., & Kozup, J. (2010). Understanding how Graphic Pictorial Warnings Work on Cigarette Packaging. *Journal of Public Policy & Marketing*, *29*(2), 265–276. https://doi.org/10.1509/jppm.29.2.265

23 Fluharty, M., Taylor, A. E., Grabski, M., & Munafò, M. R. (2017). The Association of Cigarette Smoking with Depression and Anxiety: A Systematic Review. *Nicotine & tobacco research: official journal of the Society for Research on Nicotine and Tobacco*, *19*(1), 3–13. https://doi.org/10.1093/ntr/ntw140

24 Taylor, A. E., Fluharty, M. E., Bjørngaard, J. H., Gabrielsen, M. E., Skorpen, F., Marioni, R. E., Campbell, A., Engmann, J., Mirza, S. S., Loukola, A., Laatikainen, T., Partonen, T., Kaakinen, M., Ducci, F., Cavadino, A., Husemoen, L., Ahluwalia, T. S., Jacobsen, R. K., Skaaby, T., Ebstrup, J. F., … Munafò, M. R. (2014). Investigating the possible causal association of smoking with depression and anxiety using Mendelian randomisation meta-analysis: the CARTA consortium. *BMJ open*, *4*(10), e006141. https://doi.org/10.1136/bmjopen-2014-006141

25 Anshari, D., Yong, H., Borland, R., et al (2018). Which type of tobacco product warning imagery is more effective and sustainable over time? A longitudinal assessment of smokers in Canada, Australia and Mexico. *BMJ Open, 8***.** doi: 10.1136/bmjopen-2018-021983

26 Li, L., Borland, R., Yong, H., Cummings, K. M., Thrasher, J. F., Hitchman, S. C., Fong, G. T., Hammond, D., & Bansal-Travers, M. (2015). Longer term impact of cigarette package warnings in Australia compared with the United Kingdom and Canada. *Health education research*, *30*(1), 67–80. https://doi.org/10.1093/her/cyu074

27 Drovandi, A., Teague, P.A., Glass, B., Malau-Aduli, B. (2019) Do health warnings on cigarette sticks dissuade smokers and non-smokers? A focus group and interview study of Australian university students. *Psychol Res Behav Manag*. 12:361-373 https://doi.org/10.2147/PRBM.S193754

28 Moodie, C.S., Hiscock, R,. Thrasher, J. *et al.* (2018) Perceptions of cigarette pack inserts promoting cessation and dissuasive cigarettes among young adult smokers in the UK: a cross-sectional online survey. *BMJ Open* 8:e019662. doi: 10.1136/bmjopen-2017-019662

For peer review only

29 Moodie, C., Gendall, P., Hoek, J., MacKintosh, A. M., Best, C., & Murray, S. (2019). The Response of Young Adult Smokers and Nonsmokers in the United Kingdom to Dissuasive Cigarettes: An Online Survey. *Nicotine & tobacco research : official journal of the Society for Research on Nicotine and Tobacco*, *21*(2), 227–233. https://doi.org/10.1093/ntr/ntx261

30 Statista. (2022, January 19) Percentage of population using the internet in Colombia from 2000 to 2019. https://www.statista.com/statistics/209109/number-of-internet-users-per-100-inhabitants-in-colombia-since-2000/

31 Storr, C. L., Cheng, H., Posada-Villa, J., Aguilar-Gaxiola, S., & Anthony, J. C. (2008). Adult smokers in Colombia: who isn't giving it up?. *Addictive behaviors*, *33*(3), 412–421. https://doi.org/10.1016/j.addbeh.2007.10.003

y only

Figure 1. Percentage of participants who report being aware of having seen the new warnings at Wave 1 and 2

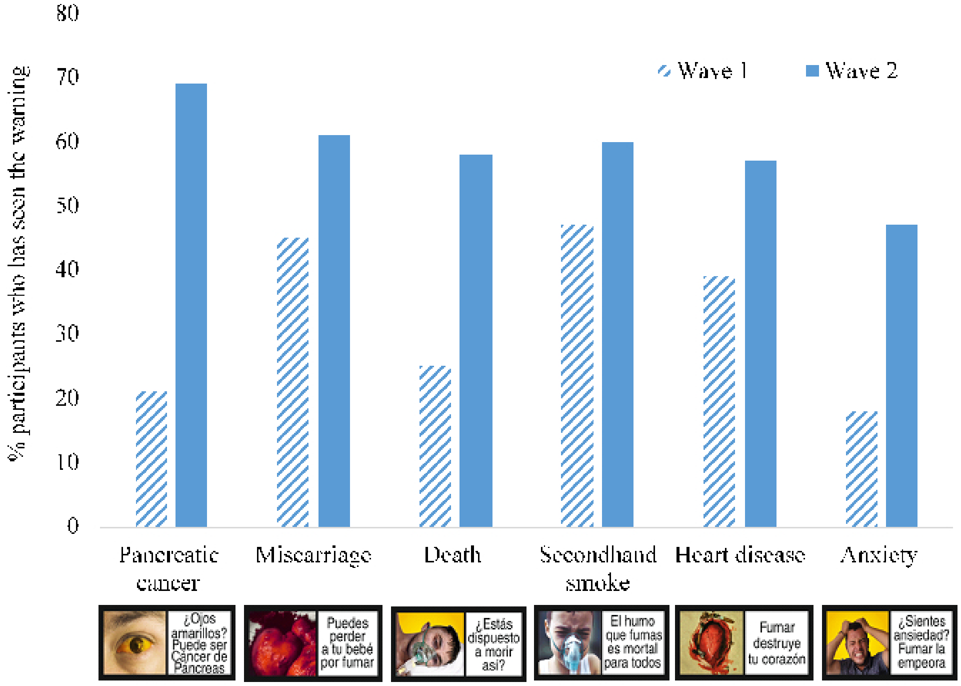

185x153mm (96 x 96 DPI)

Supplementary Materials Table 1. Survey Questions

Variable	Items	Response
Smoking behaviour - eligibility	Have you smoked at least 100 cigarettes in your lifetime?	1 = Yes<br>2 = No
	How often do you smoke cigarettes?	1 = Every day<br>2 = Every week<br>3 = Less than every week<br>4 = Not al all
Smoking heaviness – daily smokers	Approximately how many cigarettes do you smoke per day?	Provide a single number
Smoking heaviness -non-daily smokers	Approximately how many days a week do you smoke	
	On the days that you do smoke, approximately how many cigarettes do you smoke	
	Approximately how many cigarettes do you smoke per week?	
Fagerström Test for Nicotine Dependence (FTND)	How soon after you wake do you smoke your first cigarette?	1 = Within 5 minutes<br>2 = 6 to 30 minutes<br>3 = 31 to 60 minutes<br>4 = After 60 minutes
Awareness of new warnings	Have you seen this health warning on a cigarette pack?	1= Yes<br>2 = No<br>3 = Unsure
Responses to warnings seen on tobacco packs		
Negative affect	In the last week, the warning on my cigarette pack made me feel...	
	…scared	0 = Strongly disagree<br>25 = Somewhat disagree<br>50 = Neither agree nor disagree<br>75 = Somewhat agree<br>100 = Strongly agree
	…regretful	
	…disgusted	

Thinking about the warning message	When I notice my cigarette pack, I think about the message that the warning conveys	0 = Strongly disagree<br>25 = Somewhat disagree<br>50 = Neither agree nor disagree<br>75 = Somewhat agree<br>100 = Strongly agree
	When my cigarette pack is not<br>in sight, I think about the message that the warning conveys	
Attitudes towards smoking		
Perceived likelihood of harm	What is the chance that you will one day get the following diseases if you continue to smoke cigarettes?	
	…heart disease?	0 = No chance<br>25 = Low chance<br>50 = Moderate chance<br>75 = High chance<br>100 = Certain
	…cancer?	
	…a permanent breathing problem?	
Perceived severity of harm from smoking	How much would getting the following diseases because of smoking affect your life?	
	…heart disease?	0 = No chance<br>25 = Low chance<br>50 = Moderate chance<br>75 = High chance<br>100 = Certain
	…cancer?	
	…a permanent breathing problem?	
Self-efficacy	I see myself as being capable of quitting smoking in the next 2 months if I wanted to	0 = Strongly disagree<br>25 = Somewhat disagree<br>50 = Neither agree nor disagree<br>75 = Somewhat agree<br>100 = Strongly agree
Response-efficacy	Quitting smoking would lower my chances of getting a<br>disease related<br>to smoking	

Quit intentions	Are you planning to quit smoking within the next 6 months?	0 = Very unlikely<br>25 = Unlikely<br>50 = Maybe, maybe not<br>75 = Likely<br>100 = Very likely
Responses to new warnings		
Negative affect	This warning makes me feel…	
	…scared	0 = Strongly disagree<br>25 = Somewhat disagree<br>50 = Neither agree nor disagree<br>75 = Somewhat agree<br>100 = Strongly agree
	…regretful	
	…disgusted	
Believability	This warning is believable	0 = Strongly disagree<br>25 = Somewhat disagree<br>50 = Neither agree nor disagree<br>75 = Somewhat agree<br>100 = Strongly agree
Thinking about harms	This warning makes me think about the health problems caused by smoking	0 = Strongly disagree<br>25 = Somewhat disagree<br>50 = Neither agree nor disagree<br>75 = Somewhat agree<br>100 = Strongly agree
Reactance	This warning is trying to manipulate me	0 = Strongly disagree<br>25 = Somewhat disagree<br>50 = Neither agree nor disagree<br>75 = Somewhat agree<br>100 = Strongly agree
	The health effect of this warning is overblown	
	This warning annoys me	
Perceived message effectiveness	This warning discourages me from wanting to smoke	0 = Strongly disagree<br>25 = Somewhat disagree<br>50 = Neither agree nor disagree<br>75 = Somewhat agree<br>100 = Strongly agree
	This warning makes smoking seem unpleasant to me	
	This warning makes me concerned about the health effects of smoking	
	If I saw this warning on my	

	cigarette pack, I would...	
	...try to avoid thinking about it	
	…try to avoid looking at it	
	…keep the pack out of sight to avoid looking at the warning	
Knowledge of health risks	Say how much you agree or disagree with each statement below:	0 = Strongly disagree<br>25 = Somewhat disagree<br>50 = Neither agree nor disagree<br>75 = Somewhat agree<br>100 = Strongly agree
	Smoking while pregnant could cause you to lose your baby	
	Smoking causes pancreatic cancer	
	Secondhand smoke is deadly	
	Smoking damages your heart	
	Smoking causes anxiety	
	Smoking causes a slow and painful death	

Supplementary Material – Table 2. Internal consistency of latent constructs

**Latent construct**	**Number of items**	**α**	***r***
Responses to warnings on packs			
Negative affect	3 items	0.79	0.49 – 0.69
Thinking about warning message	2 items	0.66	0.5
Cognitive elaboration	2 items	0.8	0.66
Attitudes to smoking			
Perceived likelihood of harm	3 items	0.84	0.61-0.65
Perceived severity	3 items	0.87	0.66-0.71
Responses to new warnings			
**Warning 1 "Pancreatic cancer"**			
Negative affect	3 items	0.83	0.54 – 0.77
Reactance	3 items	0.68	0.39 – 0.45
Perceived effectiveness	6 items	0.87	0.34 – 0.82
**Warning 2 "Miscarriage"**			
Negative affect	3 items	0.77	0.38 – 0.79
Reactance	3 items	0.69	0.41 – 0.44
Perceived effectiveness	6 items	0.86	0.28 – 0.75
**Warning 3 "Death"**			
Negative affect	3 items	0.8	0.46 – 0.77
Reactance	3 items	0.69	0.43 – 0.44
Perceived effectiveness	6 items	0.86	0.33 – 0.82
**Warning 4 "Secondhand Smoke"**			
Negative affect	3 items	0.81	0.49 – 0.67
Reactance	3 items	0.67	0.37 – 0.44
Perceived effectiveness	6 items	0.86	0.31 – 0.80
**Warning 5 "Heart disease"**			
Negative affect	3 items	0.83	0.53 – 0.80
Reactance	3 items	0.68	0.40 – 0.44
Perceived effectiveness	6 items	0.86	0.33 – 0.80
**Warning 6 "Anxiety"**			
Negative affect	3 items	0.87	0.63 – 0.76
Reactance	3 items	0.64	0.33 – 0.43
Perceived effectiveness	6 items	0.87	0.34 – 0.85

For peer review only

Note. The internal consistency of latent constructs assessed using Cronbach's alpha and associations between the constructs using bivariate correlations.

For peer review only

Supplementary Materials Table 3. – Presence of warning theme between 2010 and 2018.

**Warning theme**	**2010**	**2011**	**2012**	**2013**	**2014**	**2015**	**2016**	**2017**	**2018**
Death							X		X
Secondhand smoke		X			X			X	X
Anxiety									X
Miscarriage		X		X			X	X	X
Heart attack / heart disease					X		X	X	X
Pancreatic cancer									X
Mouth cancer and bad breath	X								
Kidney cancer				X				X	
Breast cancer				X					
Leukemia					X				
Bladder cancer					X				
Laryngeal cancer							X		
Cancer							X		
Damaged arteries					X			X	
Thrombosis					X				
Vascular problems / disease				X					
Cerebral stroke		X							
Lung disease		X					X		
Eye problems / disease		X							
Bone damage				X					
Sexual impotence	X	X		X					
Aging	X								

Data are taken from https://www.tobaccocontrollaws.org/legislation/country/colombia/laws.
Data not available for 2012 or 2015.

All warnings are available from the Colombian Ministry of Health and Social Protection.

Colombia. Ministerio de Salud y Protección Social. (2020). Advertencias sanitarias productos de tabaco y derivados 2010 - 2021. [Piezas publicitarias;] Retrieved From: https://www.minsalud.gov.co/sites/rid/Lists/BibliotecaDigital/RIDE/VS/PP/ENT/advertencias-sanitarias-todas-vigencias.zip

For peer review only

Supplementary Material - Figure 1. Six new tobacco health warnings introduced in Colombia July 2018

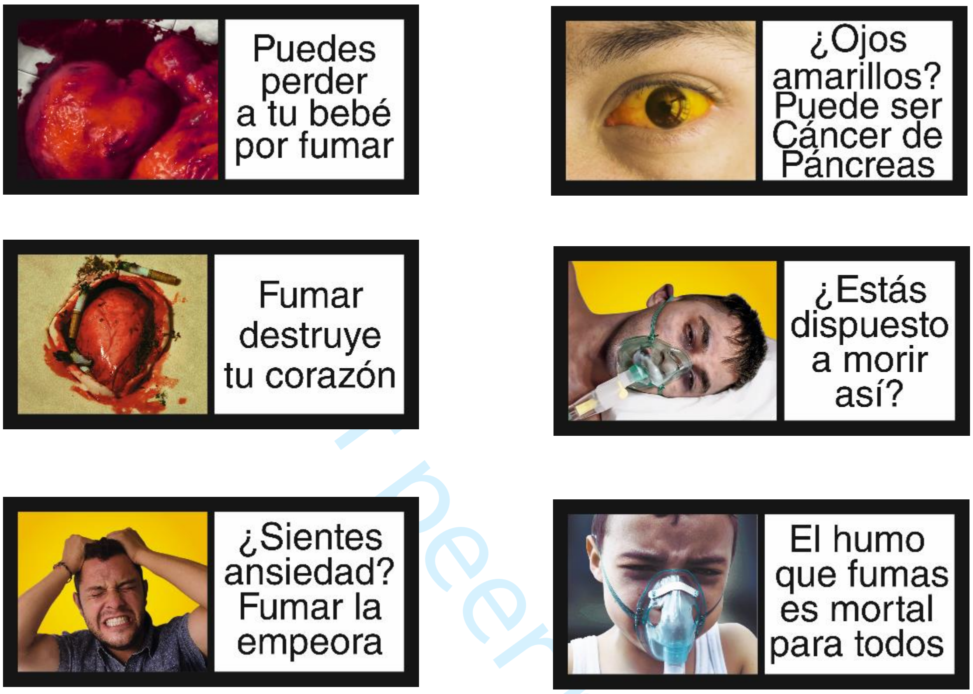

Health warning topics – clockwise *"You can lose your baby by smoking", "Yellow eyes? It could be pancreatic cancer", "Smoking destroys your heart", "Are you willing to die like this?", "Do you feel anxiety? Smoking makes it worse"* and *"The smoke you smoke is deadly for everyone".*

For peer review only

For peer review only - http://bmjopen.bmj.com/site/about/guidelines.xhtml

Supplementary Material – Figure 2. Percentage of participants who report having seen the new tobacco health warnings by data collection wave and smoker type (i.e., buys cigarette packs, buys packs and singles, buys singles).

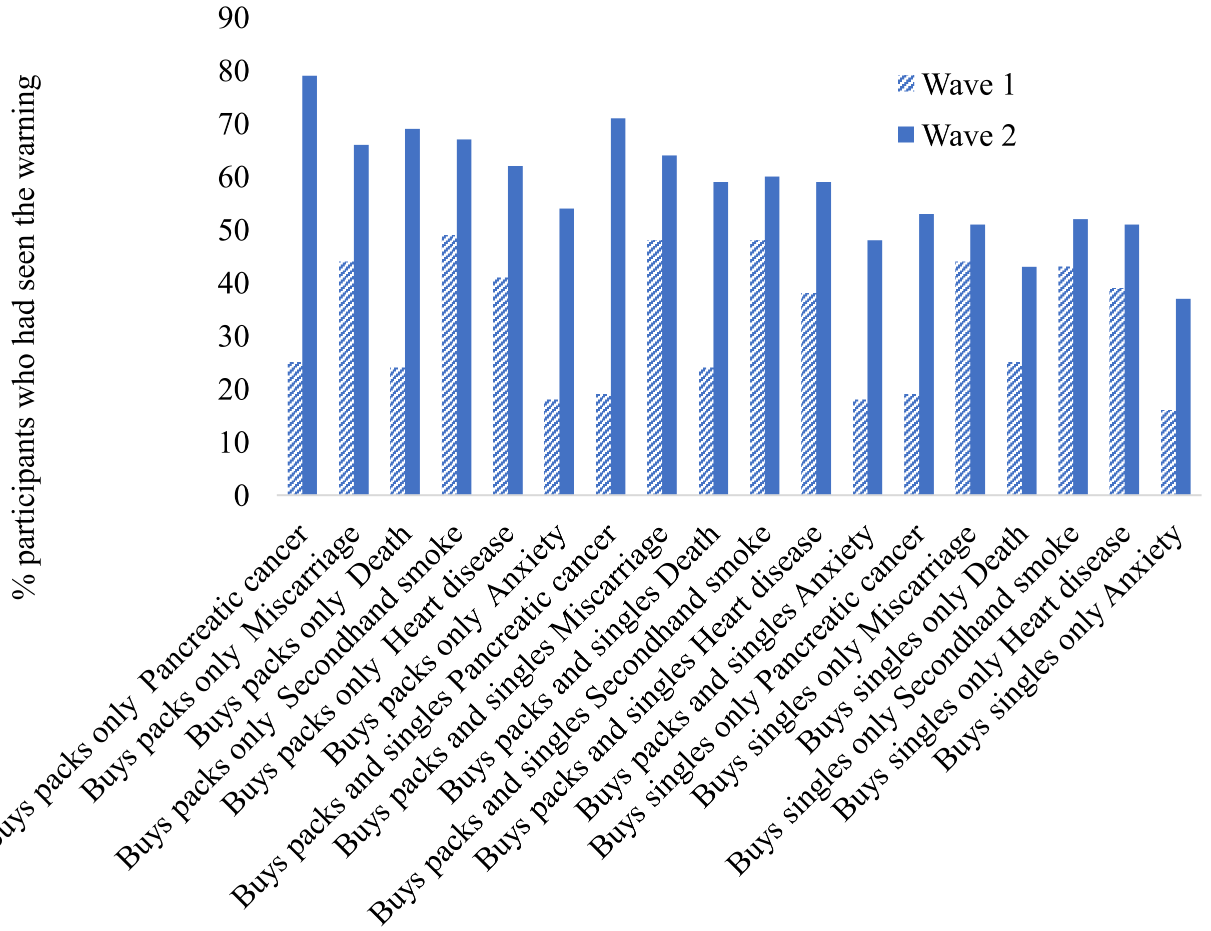

only

Supplementary Material – Figure 3. Percentage of participants who report having seen the new tobacco health warnings by data collection wave and smoking status (i.e., daily smoker, weekly smoker).

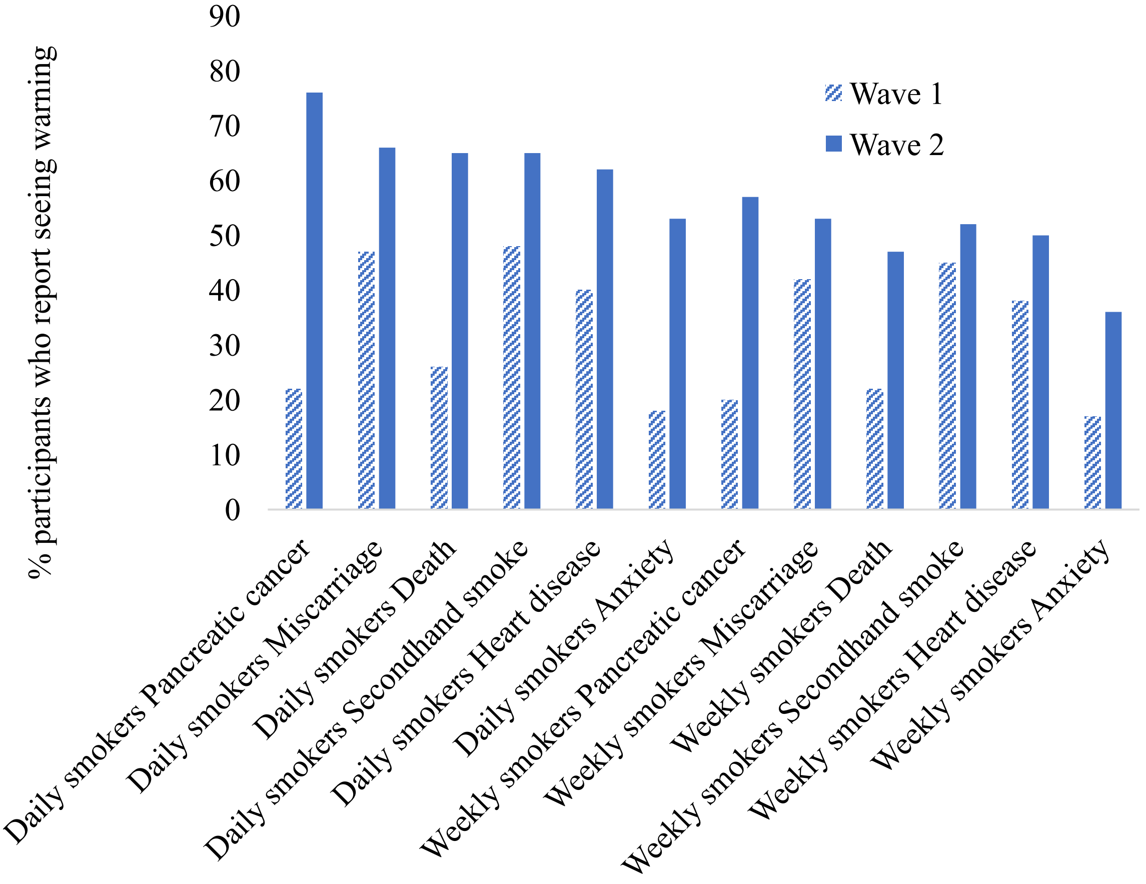

STROBE Statement—checklist of items that should be included in reports of observational studies

	Item No.	Recommendation	Page No.	Relevant text from manuscript
**Title and abstract**	1	(*a*) Indicate the study's design with a commonly used term in the title or the abstract	1.	A cross-sectional online survey of the impact of new tobacco health warnings in Colombia.
		(*b*) Provide in the abstract an informative and balanced summary of what was done and what was found	2.	**Objectives** This study aimed to explore the impact of a new set of six pictorial warnings introduced in 2018. **Design and Setting** Using a cross-sectional design, we examined awareness of the new warnings among Colombian smokers across two time points of data collection. **Participants** Adult smokers (≥ 18 years of age), defined as having smoked at least 100 cigarettes in their lifetime and currently smoking at least one cigarette per week participated at Time 1, prior to the introduction of the new health warnings in Colombia in 2018 (*n*=1985, 72% male), and at Time 2, twelve-months

For peer review only - http://bmjopen.bmj.com/site/about/guidelines.xhtml

post-introduction (n=1572, 69% male).

Primary Outcomes At each time we examined smokers' responses to warnings on packs (negative affect, thinking about warning messages, cognitive elaboration), attitudes towards smoking (perceived likelihood and severity of harm, self-efficacy, response-efficacy and quit intentions), knowledge of the health risks of smoking and responses to the new warnings (negative affect, believability, thinking about the harms, reactance and perceived message effectiveness).

Results Awareness of the warnings was low, with only 59% of smokers reporting having seen them at Time 2. Between times, we observed a reduction in negative affect towards current warnings ($p < 0.001$), reduced thinking about ($p < 0.001$) and cognitive elaboration of the warning

For peer review only - http://bmjopen.bmj.com/site/about/guidelines.xhtml

				message ($p < 0.001$), and an increase in perceived severity of warnings ($p < 0.001$). When asked about the six new health warnings, we found a reduction in negative affect (*ps* < 0.07), cognitions related to harm (*ps* < 0.01), believability (*ps* < 0.03), reactance (*ps* < 0.01) and perceived message effectiveness (*ps* < 0.02) between times. **Conclusions** Our data indicate that effectiveness was low prior to the introduction of the new health warnings and at twelve-months post-introduction. Tobacco control policy should seek to improve exposure to and noticeability of tobacco health warnings in Colombia.
**Introduction**				
Background/rationale	2	Explain the scientific background and rationale for the investigation being reported	5.	Warnings on tobacco products are the most cost-effective tool for educating smokers and non-smokers about the health risks of tobacco use.[4] Pictorial

For peer review only

For peer review only - http://bmjopen.bmj.com/site/about/guidelines.xhtml

warnings (PWs) are more likely to be noticed than text-only warnings,[5-7] are more effective at educating smokers about the health risk of smoking, increasing cognitions about health risks,[5,8] and at increasing motivation to quit smoking,[5,7,9], whilst being less vulnerable to the effects of "wear-out".[8,10] Evidence from low- and middle-income countries indicates that PWs may be more effective in these countries, being one of the few sources of information about tobacco's risks.[4,11,12] Despite this evidence there is high variability in the implementation of warnings in low- and middle-income countries.[13]

PWs were first introduced in Colombia in 2009 and since July 2010 all tobacco products have been required by law to display PWs that cover

For peer review only - http://bmjopen.bmj.com/site/about/guidelines.xhtml

For peer review only

30% of the package and are rotated annually. These requirements fall short of the World Health Organisation Framework Convention on Tobacco Control (FCTC) recommendations that warnings cover 50% of the package and that branding is replaced by plain packaging [4]. However, the Colombian Ministry of Health has called for "local evidence" on the effectiveness of larger warnings and plain packaging, before the implementation of these tobacco control measures. In Colombia, smokers' exposure to warnings may be limited. First, implementation is likely to be slow, particularly in rural areas. Each year when new warnings are introduced, retailers have 12 months to stop selling tobacco products with older warnings (referred to here as the 'phase-in period'). Second, there is a high proportion of

For peer review only - http://bmjopen.bmj.com/site/about/guidelines.xhtml

				Colombian smokers who purchase single sticks, rather than packs, who are therefore not exposed to any warnings. Given this uncertainty over the extent to which Colombian smokers are exposed to new warnings, this one-year cross-sectional study explores smokers' responses to a new set of six pictorial warnings covering 30% of the pack which were introduced in Colombia in 2018.
Objectives	3	State specific objectives, including any prespecified hypotheses	6.	First, we examined the rate of market saturation and the noticeability of the new warnings among smokers over the implementation period. Given that there is no requirement for retailers to stop selling cigarette packs with the old warnings during the 12-month phase-in period and that many Colombian smokers do not purchase cigarette packs (instead buying single cigarettes), we

For peer review only

hypothesised that even a year after implementation, awareness of warnings (a proxy marker of market saturation) will be low (less than 75% of respondents will have seen the warnings). Second, we examined smokers' responses to warnings on packs (negative affect, thinking about warning measures, cognitive elaboration), attitudes towards smoking (perceived likelihood and severity of harm, self-efficacy, response-efficacy and quit intentions), knowledge of the health risks of smoking and responses to the new warnings (negative affect, believability, thinking about the harms, reactance and perceived message effectiveness) across the survey times (baseline and 12-months post introduction). Given relatively small changes between the new and old

For peer review only - http://bmjopen.bmj.com/site/about/guidelines.xhtml

warnings and a low rate of market saturation, we hypothesised that over our assessment period there would be either a small, or no increase in negative affect, cognitions related to smoking, knowledge regarding health risks of smoking, perceived severity and likelihood of harm from smoking and quit intentions. Third, we examined smokers' responses to each of the new warnings in terms of negative affect, believability, cognitions related to harm, avoidance, reactance and perceived message effectiveness. Due to wear-out (as a result of repeated, albeit potentially limited exposure) of the warnings, we hypothesised that each of these outcomes would be greatest at baseline as compared with 12-months post exposure. Our hypotheses, methods and

For peer review only - http://bmjopen.bmj.com/site/about/guidelines.xhtml
For peer review only

				analysis plan were pre-registered in our study protocol (osf.io/qbr5v).
**Methods**				
Study design	4	Present key elements of study design early in the paper	7.	A cross-sectional online survey of Colombian smokers with two times of data collection: one prior to the introduction of the new health warnings, and one 12-months post-introduction. The study was approved by the Universidad Nacional de Colombia, Faculty of Human Sciences Ethics Committee (reference: B.VDIECH-024-18).
Setting	5	Describe the setting, locations, and relevant dates, including periods of recruitment, exposure, follow-up, and data collection	9.	The first time of data collection occurred between 21st May and 12th July 2018, prior to 21st July 2018, the date at which new warnings could start appearing on the market. The second time of data collection took place between 5th June and 1st August 2019. All procedures were identical at the two times. Participants accessed the study via Qualtrics and

For peer review only

				were first shown an information statement before completing a tick-box consent page.
Participants	6	(*a*) *Cohort study*—Give the eligibility criteria, and the sources and methods of selection of participants. Describe methods of follow-up<br>*Case-control study*—Give the eligibility criteria, and the sources and methods of case ascertainment and control selection. Give the rationale for the choice of cases and controls<br>*Cross-sectional study*—Give the eligibility criteria, and the sources and methods of selection of participants	7.	We recruited adult smokers (≥ 18 years of age), defined as having smoked at least 100 cigarettes in their lifetime and currently smoking at least one cigarette per week, from the staff and students at the Universidad Nacional de Colombia and from the general public. The research team used existing mailing lists to contact members of the university across eight cities in Colombia and attended student classes to encourage students to complete the surveys. We recruited participants from the general population through social media, word of mouth and posters in public spaces.
		(*b*) *Cohort study*—For matched studies, give matching criteria and number of exposed and unexposed		

For peer review only - http://bmjopen.bmj.com/site/about/guidelines.xhtml

		Case-control study—For matched studies, give matching criteria and the number of controls per case		
Variables	7	Clearly define all outcomes, exposures, predictors, potential confounders, and effect modifiers. Give diagnostic criteria, if applicable	9.	Participants accessed the study via Qualtrics and were first shown an information statement before completing a tick-box consent page. All text and questions were presented in Spanish. Participants completed screening questions and those who were ineligible were taken to the end of the study. Eligible participants completed demographic questions, responses to warnings on packs, knowledge of health risks and attitudes towards smoking. An attention check item was included in this section (Please select the strongly agree option to let us know that you read all of the survey instructions). Each of the six new warnings were presented individually on screen in a randomised order, and participants completed questions about each warning. After

For peer review only - http://bmjopen.bmj.com/site/about/guidelines.xhtml

For peer review only

				reading a debriefing page, participants who wished to be entered into the prize draw were directed to a second survey. Participants who participated in the first time were re-contacted at the second time to ask if they would like to participate again.
Data sources/ measurement	8*	For each variable of interest, give sources of data and details of methods of assessment (measurement). Describe comparability of assessment methods if there is more than one group	7.	Health warnings<br>The six warnings introduced on 21st July 2018 depicted six different health-related consequences of tobacco smoking (miscarriage, pancreatic cancer, heart disease, death, anxiety and secondhand smoke), (see Supplementary Materials - Figure 1).<br><br>Smoking behaviour<br>Participants completed smoking behaviour questions to determine eligibility. Demographic measures also included questions on smoker type and smoking heaviness and the first item of the Fagerström Test for

For peer review only - http://bmjopen.bmj.com/site/about/guidelines.xhtml

Nicotine Dependence (FTND) [14] (see Supplementary Materials – Table 1).<br><br>Awareness of new warnings<br>Awareness of the new pictorial warnings among Colombian smokers was assessed across two times of data collection.<br><br>Responses to warnings seen on tobacco packs<br>Responses to warnings seen on packs were assessed using measures of negative affect, thinking about warning message and cognitive elaboration.<br><br>Attitudes to smoking<br>Attitudes to smoking were assessed using measures of perceived likelihood of harm, perceived severity of harm from smoking, self-efficacy, response efficacy and quit intentions.

For peer review only

For peer review only - http://bmjopen.bmj.com/site/about/guidelines.xhtml

				Responses to new warnings<br>In response to viewing the six new health warnings, participants were asked to complete measures of negative affect, believability, thinking about harms, reactance, perceived message effectiveness.<br><br>Knowledge of health risks<br>We assessed knowledge of the six specific health risks presented on the new health warnings
Bias	9	Describe any efforts to address potential sources of bias	9.	An attention check item was included in this section
Study size	10	Explain how the study size was arrived at	7.	Due to the exploratory nature of the study we did not conduct a sample size calculation.

Continued on next page

For peer review only - http://bmjopen.bmj.com/site/about/guidelines.xhtml

Quantitative variables	11	Explain how quantitative variables were handled in the analyses. If applicable, describe which groupings were chosen and why	10.	The internal consistency of the latent constructs (e.g. negative affect, cognitive elaboration) were assessed using Cronbach's alpha. All latent constructs achieved acceptable to good internal consistency and latent variables were calculated by taking a mean of the individual items (see Supplementary Materials - Table 2).
Statistical methods	12	(*a*) Describe all statistical methods, including those used to control for confounding	9.	To examine market spread of new warnings over the implementation period we calculated percentages of participants in the two times who reported being aware of seeing each of the six new warnings. In exploratory analyses we examined how warning awareness differs between smoking status (daily, weekly smokers) and smoker type (buys cigarette packs, buys singles, buys both).<br>To examine the impact of the new warnings, we compared responses to warnings on packs, knowledge of health risks and

For peer review only - http://bmjopen.bmj.com/site/about/guidelines.xhtml

		attitudes to smoking between Time 1 and Time 2 using independent samples t-tests.
(*b*) Describe any methods used to examine subgroups and interactions	10.	We ran these analyses both including and excluding participants who were defined as unexposed to the new warnings at Time 2. We defined participants as 'unexposed' if they reported not seeing (or being unsure whether they had seen) three or more of the six new warnings at Time 2.<br><br>To examine smokers' responses to each of the new warnings, we compared negative affect, believability, thinking about the harms, reactance and perceived message effectiveness for each of the six warnings at Time 1 and Time 2 using independent samples t-tests.
(*c*) Explain how missing data were addressed	10.	Data were examined prior to analysis and participants who completed the survey after the closing date (*n*=6 in Time 1) or failed the attention check (*n*=1866 in Time 1 and *n*=988 in Time 2) were removed from further

For peer review only

				analyses. Participant characteristics are presented by data collection time in Table 1.
		(*d*) *Cohort study*—If applicable, explain how loss to follow-up was addressed<br>*Case-control study*—If applicable, explain how matching of cases and controls was addressed<br>*Cross-sectional study*—If applicable, describe analytical methods taking account of sampling strategy		
		(*e*) Describe any sensitivity analyses	N/A	
**Results**				
Participants	13*	(a) Report numbers of individuals at each stage of study—eg numbers potentially eligible, examined for eligibility, confirmed eligible, included in the study, completing follow-up, and analysed	10.	Data were examined prior to analysis and participants who completed the survey after the closing date (*n*=6 in Time 1) or failed the attention check (*n*=1866 in Time 1 and *n*=988 in Time 2) were removed from further analyses.
		(b) Give reasons for non-participation at each stage	10.	As above
		(c) Consider use of a flow diagram	N/A	
Descriptive data	14*	(a) Give characteristics of study participants (eg demographic, clinical, social) and information on exposures and potential confounders	10.	Participant characteristics are presented by data collection time in Table 1.
		(b) Indicate number of participants with missing data for each variable of interest	N/A	
		(c) *Cohort study*—Summarise follow-up time (eg, average and total amount)		
Outcome data	15*	*Cohort study*—Report numbers of outcome events or summary measures over time		
		Case-control study—Report numbers in each exposure category, or summary measures of exposure		
		Cross-sectional study—Report numbers of outcome events or summary measures	22-25	Tables 2-4

For peer review only - http://bmjopen.bmj.com/site/about/guidelines.xhtml

Main results	16	(*a*) Give unadjusted estimates and, if applicable, confounder-adjusted estimates and their precision (eg, 95% confidence interval). Make clear which confounders were adjusted for and why they were included	11-13	**Responses to warnings seen on tobacco packs** As shown in Table 2, there was evidence of a decrease in negative affect, thinking about warning messages and cognitive elaboration between Time 1 and 2. These findings did not differ when including only participants defined as 'exposed' at Time 2.<br><br>**Attitudes to smoking** There was strong evidence of an increase in perceived severity between Time 1 and 2 (Table 2), although this difference was not evident after including only participants defined as exposed to the warnings at Time 2. The analysis including only participants exposed at Time 2 also indicated strong evidence of a decrease in self-efficacy and quit intention between times. There was no evidence for any other differences.<br><br>**Knowledge of health risks** There was strong evidence of an increase in mean knowledge of health risks

For peer review only

between times across all warnings, when including only participants defined as exposed at Time 2 (see Table 2). For individual warnings, there was evidence of a small, but statistically meaningful increase in knowledge for the health risks “Smoking causes pancreatic cancer” and “Smoking causes anxiety” between times. The analysis including only participants exposed to the warnings at Time 2 indicated that there was also evidence of an increase between times in knowledge for the health risk “Smoking while pregnant could cause you to lose your baby”. These data were highly skewed, however, with high rates of participants giving scores of either 0 (strongly disagree) or 100 (strongly agree) for each knowledge question (See Table 3).

Responses to new warnings

Differences in responses to the six new health warnings are shown in Table 4.

For peer review only - http://bmjopen.bmj.com/site/about/guidelines.xhtml

For peer review only

	For the health warning "Miscarriage" there was strong evidence of a decrease in negative affect, cognitions related to harm, believability and perceived message effectiveness between times. For the health warning "Pancreatic cancer" there was strong evidence of a decrease in cognitions related to harm and reactance and an increase in believability between times. For the health warning "Anxiety" there was no evidence of any differences in response to the warning. For the health warning "Heart disease" there was strong evidence of a decrease in negative affect, cognitions related to harm and perceived message effectiveness between wavs. For the health warning "Second-hand smoke" there was strong evidence of a decrease in negative affect, cognitions related to harm, believability and perceived message effectiveness between times. Finally, for the health warning "Death" there was strong evidence of a decrease

For peer review only - http://bmjopen.bmj.com/site/about/guidelines.xhtml

		in negative affect, cognitions related to harm and perceived message effectiveness between times. There was no evidence for any other differences.
	(*b*) Report category boundaries when continuous variables were categorized	
	(*c*) If relevant, consider translating estimates of relative risk into absolute risk for a meaningful time period	

Continued on next page

For peer review only

For peer review only - http://bmjopen.bmj.com/site/about/guidelines.xhtml

Other analyses	17	Report other analyses done—eg analyses of subgroups and interactions, and sensitivity analyses		N/A
**Discussion**				
Key results	18	Summarise key results with reference to study objectives	14.	Using a cross-sectional design, we examined how smokers responded to the new set of warnings introduced in 2018 and find that they had relatively limited impact on knowledge of risk and attitudes towards smoking. We do however find some interesting differences between the warnings themselves, and together these findings have important implications for health warning design and implementation in Colombia and worldwide.
Limitations	19	Discuss limitations of the study, taking into account sources of potential bias or imprecision. Discuss both direction and magnitude of any potential bias	17-18	Our findings should also be considered in light of the following limitations. Our study is limited by the use of self-reported data on the perceived effectiveness of health warnings, where it is difficult to determine whether these responses accurately reflect behaviour. We were unable to make a direct comparison between participants' responses

For peer review only - http://bmjopen.bmj.com/site/about/guidelines.xhtml
For peer review only

	between study times, as different samples of smokers were recruited. We also observed that many participants failed our attention check, highlighting the limitation of using online self-report data. Our participants may not be representative of the general population in Colombia and in particular smokers. Internet penetration is estimated at 65% in Colombia [29], where individuals without internet access would not have been able to participate in our survey. Further, our sample of smokers were on average younger, more educated and more likely to be male compared with Colombian smokers reported in previous research [30]. Therefore, our sample may limit the generalisability of our findings to all smokers in Colombia, where further research on the effectiveness of tobacco health warnings for all Colombian smokers is warranted.

For peer review only

Interpretation	20	Give a cautious overall interpretation of results considering objectives, limitations, multiplicity of analyses, results from similar studies, and other relevant evidence	18.	As hypothesised, we found that awareness of the warnings was low. Across the six warnings, the average percentage of participants reporting having seen them at Time 2 was 59%. This is particularly low, given that even at Time 1, 33% of participants incorrectly reported having seen the warnings. Given this low level of awareness, it is unsurprising that we see either no changes, or relatively small changes in our measures of responses to warnings on packs, overall knowledge of health risks and attitudes to smoking between Times 1 and 2 – these are outcomes we would expect to improve, had the new warnings made an impact on smokers. Although the differences were small, contrary to our hypothesis, participants reported *lower* negative affect, thinking about the warnings and cognitive elaboration of the warnings on their cigarette packs at Time 2. Even when we only include those

For peer review only - http://bmjopen.bmj.com/site/about/guidelines.xhtml

participants we defined as 'exposed' to the new warnings at Time 2, these unexpected effects persisted. We saw little to no change in attitudes to smoking between times, although there was some evidence that participants at Time 2 thought that the severity of smoking harms were greater, and they had greater knowledge of the health risks of smoking. However, again, contrary to expectations, when we only examined these effects among participants who we defined as exposed to the new warnings at Time 2, self-efficacy and quit intentions were lower at Time 2.

Together, these findings suggest that the new warnings had relatively little impact on smokers. We consider there to be a number of complementary explanations for this. First, although Time 2 was conducted 12 months after the new warnings were permitted for sale, it is likely that they did not become commonplace for some time after that, as there is no legal

For peer review only - http://bmjopen.bmj.com/site/about/guidelines.xhtml

requirement for retailers to stop selling packs with older tobacco warnings until 12 months after the implementation period. It is possible that this slow phase-in diminished warnings' impact and meant that even a year after they (in theory) started being available on packs for sale, few participants recalled having seen them. Second, low awareness of warnings is also likely related to the extent to which smokers engage with cigarette packs. Despite being prohibited by law, many Colombian smokers purchase single cigarettes, [15] and therefore may not be exposed to health warnings at all. Indeed, we find that awareness of the new warnings is greatest for those who smoke daily and those who buy packs.

We do however find some interesting differences between the warnings themselves. As might be expected, we see the highest levels of participants incorrectly reporting at Time

For peer review only - http://bmjopen.bmj.com/site/about/guidelines.xhtml

1 that they had seen the new warnings for those with themes used in previous years ('miscarriage', 'heart disease', 'secondhand smoke' were used most recently in 2017 and 'death' was used in 2015; see Supplementary Materials -Table 3). For these four 'familiar' warnings (similar theme, but different wording and pictorial), we see high levels of believability and knowledge of these health risks at both times and only a small proportion of participants reporting that they 'strongly disagreed' that smoking caused these health risks.

However, for all four of these familiar warnings, we observe reductions between times in almost all specific responses to them (e.g., perceived message effectiveness and negative affect). These data may provide support for previous work which has observed a declining impact of warnings (i.e., "wear-out") over time. [7,16-19]. However, wear-out assumes that the warnings

For peer review only - http://bmjopen.bmj.com/site/about/guidelines.xhtml

were initially effective, but become less effective with time. The six warnings tested here received relatively low scores on all measures at Time 1 and although statistically significant, the observed declining impact of warnings at Time 2 was small and may not reflect a meaningful change. These low scores for the warnings may suggest that they are not effective at eliciting responses such as negative affect, cognitions related to harm, believability, and perceived message effectiveness and it is possible that their effectiveness may be limited by the theme or disease depicted in the warning.

There are two warning themes which were new on Colombian cigarette packs in 2018, ‘pancreatic cancer’ and ‘anxiety’. As expected, for these novel warnings, we saw a much smaller percentage of participants incorrectly reporting that they had seen them at Time 1 than the four familiar warnings. However, interestingly, at Time 2, we

For peer review only - http://bmjopen.bmj.com/site/about/guidelines.xhtml
For peer review only

saw divergent levels of awareness for these two warnings. The largest percentage of participants reported having seen the pancreatic cancer warning of all warnings at Time 2, but the lowest percentage of all participants reported having seen the anxiety warning. We suggest this may be due to the pictorial used, with the pancreatic cancer warning displaying a close-up of a yellow eye, which is arguably novel and memorable, while the anxiety warning depicted a man pulling at his hair. Previous research has suggested that more “severe” or grotesque warnings increase smokers’ intentions to quit and are rated as more believable and effective than less severe warnings. [20,21]

Knowledge of the risk of smoking for both pancreatic cancer and anxiety was low at Time 1 and at this time-point we saw the highest percentages of participants reporting that they ‘strongly disagreed’ that smoking caused these conditions. The

For peer review only - http://bmjopen.bmj.com/site/about/guidelines.xhtml

inclusion of these new warning messages reflects the priority for the Colombian Ministry of Health to inform the public about "lesser known" smoking health harms. We saw increased knowledge of these two health risks at Time 2, and fewer participants selecting 'strongly disagree' particularly for anxiety, although knowledge was still lower than for most other health risks. Despite this, these increases suggest that novel warnings can impact smokers' knowledge of smoking risks. We also saw that the anxiety warning had the lowest response scores at both times compared with all other warnings (particularly negative affect, believability, perceived message effectiveness and cognitions related to harm). We suggest that these low scores, combined with low 'knowledge' scores, are in line with the inconsistent cross-sectional evidence regarding the relationship between smoking and

For peer review only - http://bmjopen.bmj.com/site/about/guidelines.xhtml

anxiety,[22] and evidence from Mendelian randomisation studies which find no causal relationship between smoking and the onset of anxiety.[23] We suggest that it is important to consider the veracity of the health risk claims depicted in health warnings and we raise the important question of whether including health risks which are not supported by the evidence might reduce the overall impact of warnings.

Warnings have repeatedly been shown to be a cost-effective tool for communicating the health risks of smoking to smokers' and non-smokers.[4] For low- and middle-income countries, warnings are proposed to be even more effective.[4] However, for this to occur, smokers must be exposed to warnings, where increased noticeability may improve warning effectiveness. Our data suggest that strategies for increasing exposure and noticeability of warnings are required in Colombia. Currently, warnings in

For peer review only

For peer review only - http://bmjopen.bmj.com/site/about/guidelines.xhtml

				Colombia cover 30% of the pack, with the FCTC recommending that warnings should cover at least 50%. Noticeability and subsequent effectiveness of warnings may be improved by the introduction of larger pictorial health warnings. [24,25]. Additionally, as a large proportion of smokers purchase single cigarettes rather than packs, including health warning messages on individual cigarette sticks represents a novel and effective method for reducing tobacco use.[26-28]
Generalisability	21	Discuss the generalisability (external validity) of the study results	18.	Health warnings represent a cost-effective tool for communicating the health risks of smoking in low- and middle-income countries. However, our data suggest that current warnings have a limited impact on knowledge of risk and attitudes towards smoking.
**Other information**				
Funding	22	Give the source of funding and the role of the funders for the present study and, if applicable, for the original study on which the present article is based	19.	This research was funded by an ESRC New Investigator's Award, awarded to Dr Olivia

For peer review only - http://bmjopen.bmj.com/site/about/guidelines.xhtml

For peer review only

	Maynard (ES/R003424/1) and a University of Bristol Global Challenges Research Ad Hoc Fund.

*Give information separately for cases and controls in case-control studies and, if applicable, for exposed and unexposed groups in cohort and cross-sectional studies.

Note: An Explanation and Elaboration article discusses each checklist item and gives methodological background and published examples of transparent reporting. The STROBE checklist is best used in conjunction with this article (freely available on the Web sites of PLoS Medicine at http://www.plosmedicine.org/, Annals of Internal Medicine at http://www.annals.org/, and Epidemiology at http://www.epidem.com/). Information on the STROBE Initiative is available at www.strobe-statement.org.

For peer review only

For peer review only - http://bmjopen.bmj.com/site/about/guidelines.xhtml
